# The practice of historical ecology: What, when, where, how and what for

**DOI:** 10.1007/s13280-024-01981-1

**Published:** 2024-03-05

**Authors:** Aarón Moisés Santana-Cordero, Péter Szabó, Matthias Bürgi, Chelsey Geralda Armstrong

**Affiliations:** 1 Departamento de Geografía, Universidad de Salamanca, Calle Cervantes s/n, 37001 Salamanca, Spain; 2 Grupo Geografía, Medio Ambiente y Tecnologías de la Información Geográfica, Instituto de Oceanografía y Cambio Global, IOCAG, Universidad de Las Palmas de Gran Canaria, ULPGC, Parque Científico Tecnológico, Taliarte, 35214 Telde, Spain; 3 Department of Vegetation Ecology, Institute of Botany of the Czech Academy of Sciences, Lidická 25/27, 60200 Brno, Czech Republic; 4 Department of Environmental Studies, Faculty of Social Studies, Masaryk University, Joštova 10, 60200 Brno, Czech Republic; 5 Research Unit Land Change Science, Swiss Federal Research Institute WSL, 8903 Birmensdorf, Switzerland; 6 Institute of Geography, University of Bern, 3012 Bern, Switzerland; 7 Indigenous Studies, Simon Fraser University, 8888 University Drive, Burnaby, BC V5A 1S6 Canada; 8 Resource and Environmental Management, Simon Fraser University, 8888 University Drive, Burnaby, BC V5A 1S6 Canada

**Keywords:** Historical ecology, Interdisciplinarity, Long-term studies, Mixed methods approaches

## Abstract

In recent decades, there has been a growing number of studies exploring the historical dimensions of the interconnectedness of human societies and the environment. A core approach in this field is historical ecology. We analyzed 544 historical-ecological papers to assess patterns and trends in the field. We found a high degree of interdisciplinarity with a focus on local case studies, of periods of fewer than 500 years, analyzing archival sources through quantitative approaches. The proportion of papers containing management recommendations has increased over time. To make historical ecology globally relevant, more effort should be made to utilize studies across languages, borders and worldviews. We call for high standards regarding the use of social scientific methodologies. Lastly, we argue that fostering longer-term studies and assessing the real-life impact of policy recommendations emerging from historical ecology can help the discipline better contribute solutions to the challenges facing humanity in an uncertain future.

**Supplementary Information:**

The online version contains supplementary material available at 10.1007/s13280-024-01981-1.

## Introduction

Even though the study of human societies and environmental phenomena have been traditionally separated in scholarly contexts, research that more fully considers people and ecosystems as complex and closely interlinked entities has been growing in recent decades. One such integrative approach is historical ecology, which includes sources and methods derived from history, biology, ecology, geography, and archaeology/anthropology with special focus on environmental conservation, restoration and management (Balée 2006; Higgs et al. 2014; Szabó 2015). Despite the increasing awareness and appreciation for the field, a commonly shared and widely accepted notion of what historical ecology encompasses is lacking. Szabó (2015) argued that historical ecology does not have a unified methodology, a phenomenon generally acknowledged among practitioners of the field. For example, Rick and Lockwood (2013) called for standardized methodologies in historical ecology, Armstrong et al. (2017) advocated for more consilience with anthropological/archaeological methods, Beller et al. (2017) invited researchers to work towards core principles of historical ecology, and Bürgi et al. (2017) suggested forming interdisciplinary teams to optimize research agendas. Whereas this diversity in approaches might cause occasional confusion and misunderstanding, it can also be seen as an advantage, reflecting how widely the call to address the historical dimension of ecosystems and landscapes has been taken up (Crumley 1994; Balée 2006; Tappeiner et al. 2021). These disparate approaches to historical ecology are further exemplified by the pluridisciplinary discourses on how to bridge the natural sciences, social sciences, and humanities (Crumley 1994, 2017; Szabó and Hédl 2011) or why history matters in ecology (Szabó 2010) and in landscape ecology (Rhemtulla and Mladenoff 2007).

The multiplicity of approaches used to study the historical dimensions of societies and their lived landscapes makes historical ecology a powerhouse for interdisciplinarity. Consequently, one of the core features of historical-ecological studies is that they are based on combining qualitative and quantitative sources as well as methods from the humanities and the social and natural sciences (Bürgi and Gimmi 2007; Beller et al. 2017). Historical-ecological research therefore fosters the application of mixed method approaches. This is a strength and a challenge at the same time. Undoubtedly, only inter- and multidisciplinary approaches will enable researchers to address the complexity of development pathways of cultural landscapes or social-ecological systems, but, at the same time, this blurs the boundaries of what kind of studies should be included in the field and maintains internal division. Thus, further investigations into the variability of theoretical and methodological approaches are necessary for the advancement of historical ecology, where a concise scrutiny of methodological approaches and data sources is lacking. Previous studies have focused on the use of historical sources (Rymer 1979; Forman and Russell 1983, Santana-Cordero et al. 2014), the characteristics of such sources (Edmonds 2001; Vellend et al. 2013; Pooley 2018) and qualitative methods (Santana-Cordero and Szabó 2019). However, beyond generalities, there is no comprehensive synthesis on the practice of historical ecology—what kind of sources and methods are actually used and which sites, bioregions, and ecosystems have garnered more attention than others.

There is general consensus that historical ecology can have a strong applied aspect (Swetnam et al. 1999). With the deepening of the environmental crisis in the past decades, calls to utilize this potential have become more numerous (Higgs et al. 2014; Beller et al. 2020). However, it is not known how many historical-ecological works have in fact engaged with the potential application of their results in environmental conservation/restoration (but see Beller et al. 2020), and whether the proportion of such works in the overall production of historical-ecological research has increased with growing environmental concerns. A synthesis of the above issues would be useful in advancing historical ecology as an independent discipline but also in identifying research gaps and promising avenues for future research.

To assess the global variability and applicability of methodological approaches in historical ecology, to explore underlying features and structures that drive this variability, and to analyse how much historical-ecological work is geared towards applying the results in environmental conservation/restoration, we conducted a review of peer-reviewed papers in the field. Specifically, this research explores the many integrations of historical and ecological sources and methods published between 1981 (date of the first indexed publication on historical ecology in the SCOPUS database) and 2019. We provide a general review of historical-ecological research, including what, where, when, how and what for it has been practiced. We ask (i) where and what types of historical-ecological studies have been conducted and published, (ii) what sources and methods have been used, and (iii) whether papers have contained management recommendations. Based on these queries, we discuss the spatial, temporal, methodological and application strengths and weaknesses of the field and identify areas requiring further attention from the historical ecology community—namely, where more rigorous and incisive research is required in an increasingly uncertain environmental and climate future.

## Methods

Scientific literature on historical ecology has grown rapidly in recent years. A simple Google Scholar search using “historical ecology” for the 5 years between 2015 and 2019 returns as many as 7200 entries. To this number one could add many more works that would qualify as historical ecology but were defined by their authors as, for example, forest history or palaeoecology. While reviews based on automated data extraction can cope with any number of papers (Marshall and Wallace 2019), we had to conduct a typical literature review (sensu Grant and Booth 2009), which involves manual extraction of information from each paper. We therefore had to reduce the number of papers analyzed to a manageable size while trying to keep the representativeness of the dataset. To achieve this, we gathered and analyzed papers where authors described their research using the term historical ecology. Initial searches were sourced from the SCOPUS database (Elsevier), using the term “historical ecology” as author keyword (AUTHKEY: keywords established by the authors in their publications). The query covered the period between 1981 and 2019 and initially resulted in 593 documents. Contributions with the status “in press” were excluded. Results showed that the term “historical ecology” is also used in the field of phylogeny/speciation, however, it has a fundamentally different meaning in this context (compared with more mainstream approaches, see Szabó 2015). Consequently, such papers were removed from the dataset resulting in a total of 544 papers (for a complete list of papers, see Supplementary material). Almost all research papers were written in English, with a few exceptions in French, German, Portuguese, Russian and Spanish. For all 544 documents included in this study, we evaluated document type, study area, study period, sources, methods/techniques, other observations, and management recommendations, which were then coded, classified and analysed. As practitioners of historical ecology typically use a multitude of methods and data sources, we further evaluated how authors systematically combined sources and methods, which we expected to differ along disciplinary approaches and regional expertise. Because we were interested in the application aspect of particular research results, we have excluded editorials and theoretical-methodological papers from the analysis of management recommendations. The variables and categories analyzed are summarized in Table 1.

**Table 1:** Variables and categories considered in this review

Variable	Categories
Document type	Case-study; theoretical-methodological; review; editorial; meta-analysis
Study type	Local; comparative 2 regions; comparative > 2 regions
Study area (for local studies)	0–100 ha; 101–500 ha; 501–1000 ha; 1001–10 000 ha; 10 001–100 000 ha; 100 001–1 000 000 ha; > 1,000,000 ha
Country	Name of country/ies
Study period	Absolute and length: 0–100 years; 101–500 years; 501–1000 years; > 1000 years
Sources	Historical written documents (HWD); historical maps; pictures; aerial photographs/ortho-photos; satellite imagery; Lidar; scientific literature; current geographical information; historical statistical data; database; current statistical data; other documentary evidence; interviews with lay persons; interviews with experts; current vegetation; human archaeological data; zooarchaeological data; pollen; plant macrofossils; charcoal; other biological data; sediment properties; other physical data
Methods/techniques	Statistical analysis; lab analysis; qualitative analysis/description; geographic information systems/spatial analysis; modelling; quantitative procedure; geo-statistics; photo-interpretation; repeated photography
Other observations	
Management and policy recommendations	Yes/no

Given the recent proliferation of historical-ecological research, we acknowledge that the 2019 cutoff for our data has excluded important research published since then (e.g. Stuessy 2020; Thurstan 2022; Armstrong et al. 2022; Decocq 2022; Rivera-Collazo 2022; Whitaker et al. 2023; Wood et al. 2023). Given the impacts of the COVID-19 pandemic, this research, conducted before its onset, omits newer works. However, a scan of SCOPUS literature published between 2020 and April 2023 indicates that the trends outlined below are consistent with recent published studies. Furthermore, we recognize that our queries do not include historical-ecological grey literature, published outside of academic contexts (e.g., Baumgarten et al. 2018), and many other significant works that were not defined as historical ecology by their author keywords. Our results are ultimately valid for self-identifying historical-ecological research between 1981 and 2019; we nonetheless believe that these 544 papers constitute a sample that sufficiently represents the whole discipline and that adding more works is unlikely to change the overall results. In addition, in the Discussion section we include important works beyond our database to expand the relevance of our conclusions.

## Results

**Fig. 1:**
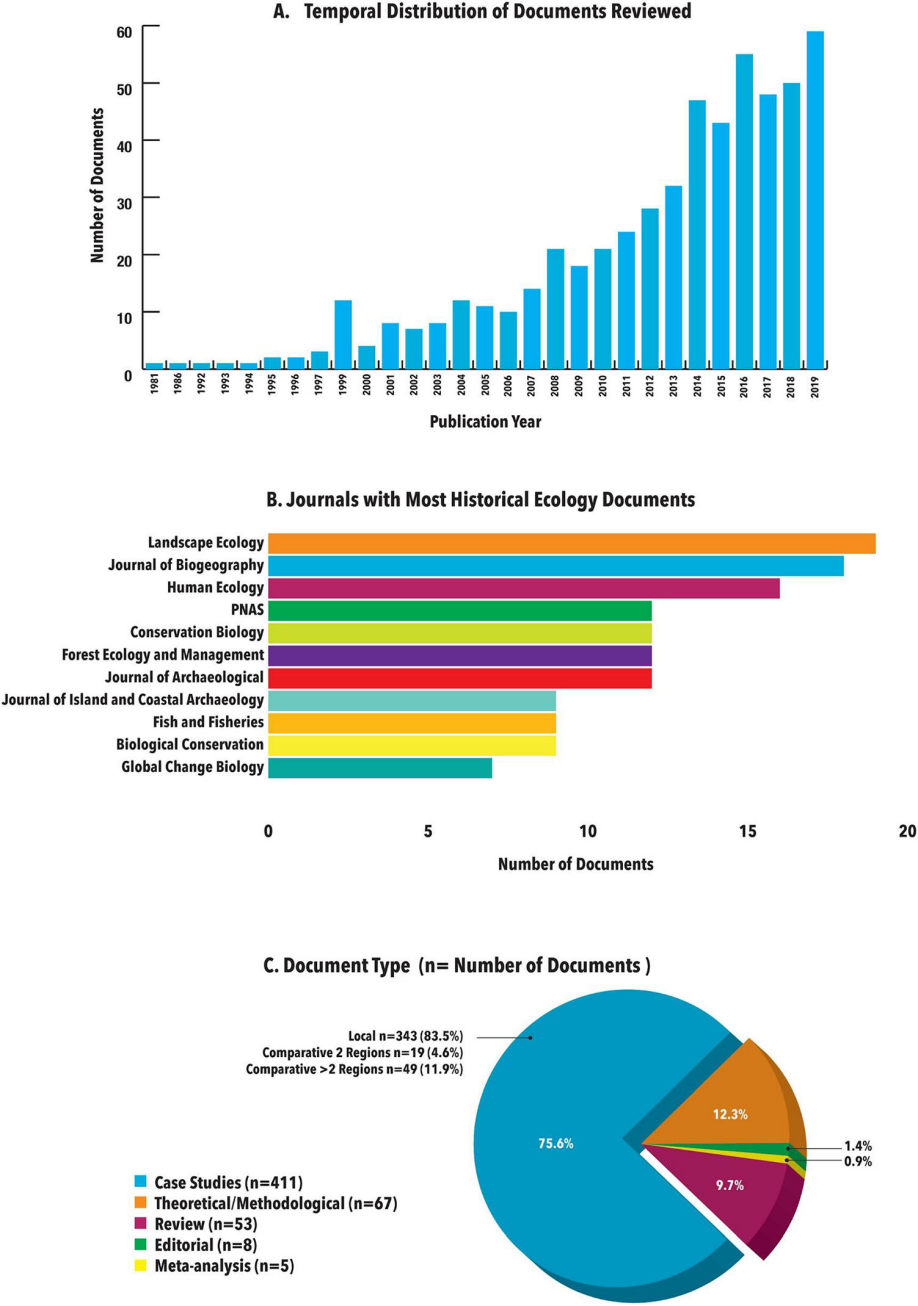
**A** Temporal distribution of the papers reviewed in this study. **B** Journals in which at least nine papers on historical ecology appeared in 1981–2019. **C** Documents in our review coded according to type

The temporal distribution of the 544 documents showed a continuous increase from the year 1999 to 2019, with very few studies published prior to 1998, illustrating the scientific community’s steadily growing interest in historical ecology (Fig. 1). Results indicate that the journals in which historical-ecological research was published reflected, as expected, a diversity of disciplinary contexts. Studies included in our survey appeared in an impressively diverse list of outlets, totaling 152 different peer-reviewed journals. The most common were *Landscape Ecology* (19 papers), the *Journal of Biogeography* (18 papers), and *Human Ecology* (16 papers). Journals hosting the highest number of historical-ecological papers were decidedly ecologically oriented (Fig. 1). The majority of research papers evaluated (411, i.e., 76%) were case studies, whereas theoretical-methodological papers (12%), and review papers (10%) accounted for fewer contributions, but were still well-represented. The majority of case studies (63%) addressed single regions, but comparative studies including two or more regions (13%) were also relatively frequent (Fig. 1).

### Study area and period

**Fig. 2:**
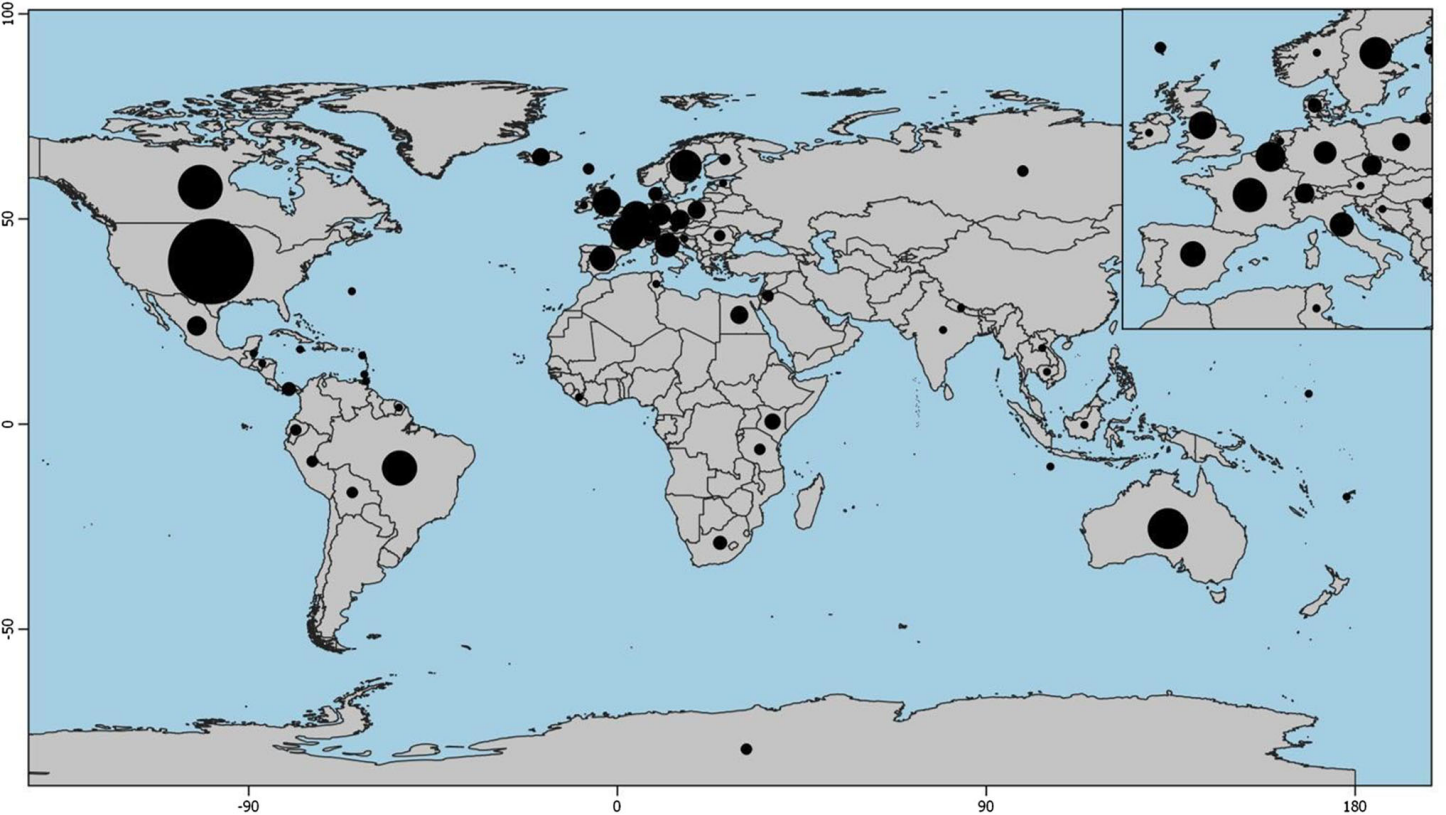
Location of study areas addressed in the case studies included in this review

Regarding the geographical distribution of the research papers analyzed, there was a dominance of studies focusing in the United States and Europe (Fig. 2). When broken down by country, settler colonial nations like Canada (31 research papers) and Australia (25 research papers) were well-represented alongside the United States (113 research papers). Those studies conducted in Europe (126 research papers) were spread out over many smaller countries.

**Fig. 3:**
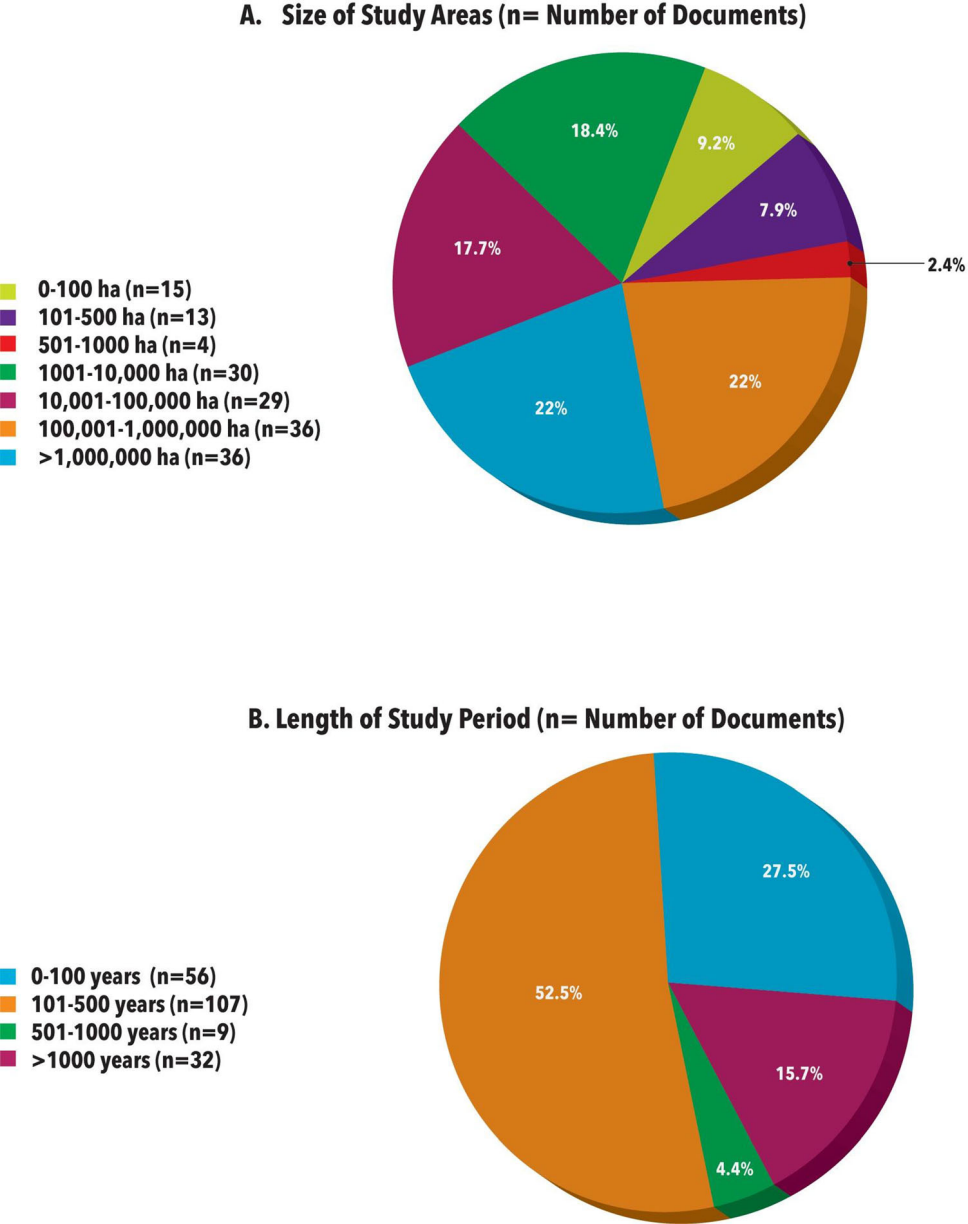
**A** Size of the study area covered in the studies reviewed. **B** Length of study period in the studies reviewed

Surprisingly, only 36% of the papers (148 of 544) included information regarding the exact size of the study area. Of these, 26 papers reported on relatively small-scale study areas (</= 500 ha), and 122 papers had study areas of > 500 ha (Fig. 3). Information on the temporal range of historical-ecological papers was similarly patchy. Of the 544 papers, only 204 papers (38%) specified the length of study period they focused on. Results revealed a focus on periods of 101 to 500 years before present (52.5%), followed by shorter study periods (less than 100 years). 16% of all studies included long-term investigations, spanning more than 1000 years (Fig. 3). We detected no clear trends in study area or period over the four decades covered by the papers we analyzed.

### Sources and methods

**Fig. 4:**
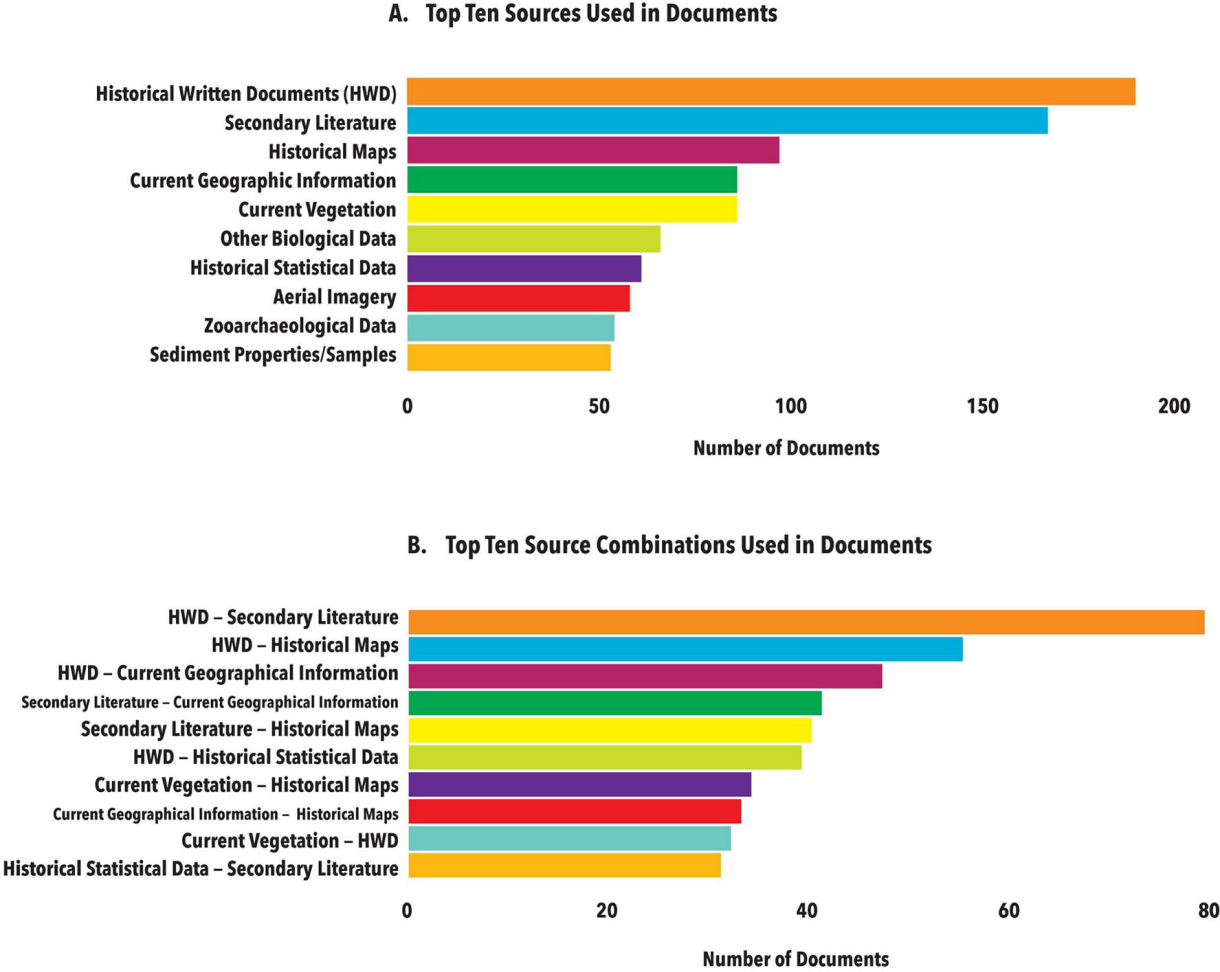
**A** Top-10 sources used in the studies reviewed. **B** Top-10 combinations of sources

Results confirmed the expected depth and breadth of sources and methods applied in historical ecology. The overwhelming majority of studies (79.2%) used several types of historical, ecological and archaeological/anthropological sources—on average 2.31 source types per document. 190 studies reported the use of historical written documents as one of the source types, followed by secondary literature (167 studies) and historical maps (97 studies) (Fig. 4). The most prevalent source combinations included historical written documents with secondary literature and historical maps (Fig. 4).

**Fig. 5:**
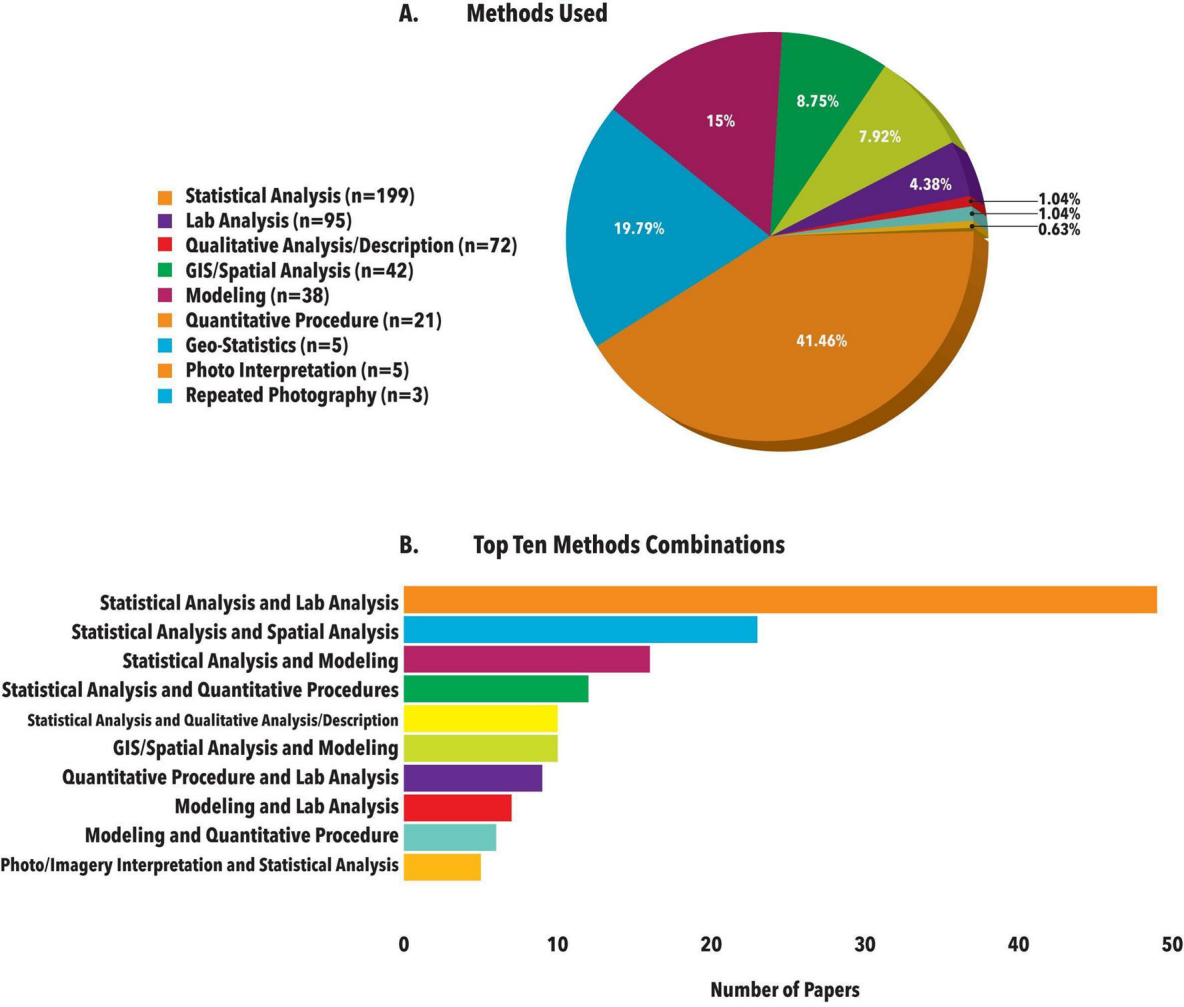
**A** Methods used in the studies reviewed. **B** Top-10 combinations of methods

Regarding the methods applied in each study (1.41 methods per study), there was a widespread application of statistical methods. Just under half (199 of 480 research papers) of the papers utilized quantitative analyses (e.g., statistics or models) (Fig. 5). Laboratory analyses (19.79%) and qualitative analysis/description (15%) were also common, representing very different strands of scientific traditions and therefore illustrating the methodological diversity within the field. The widespread use of statistical analyses and modelling was also reflected in the list of the most prevalent combinations of methodologies (Fig. 5).

### Management recommendations

**Fig. 6:**
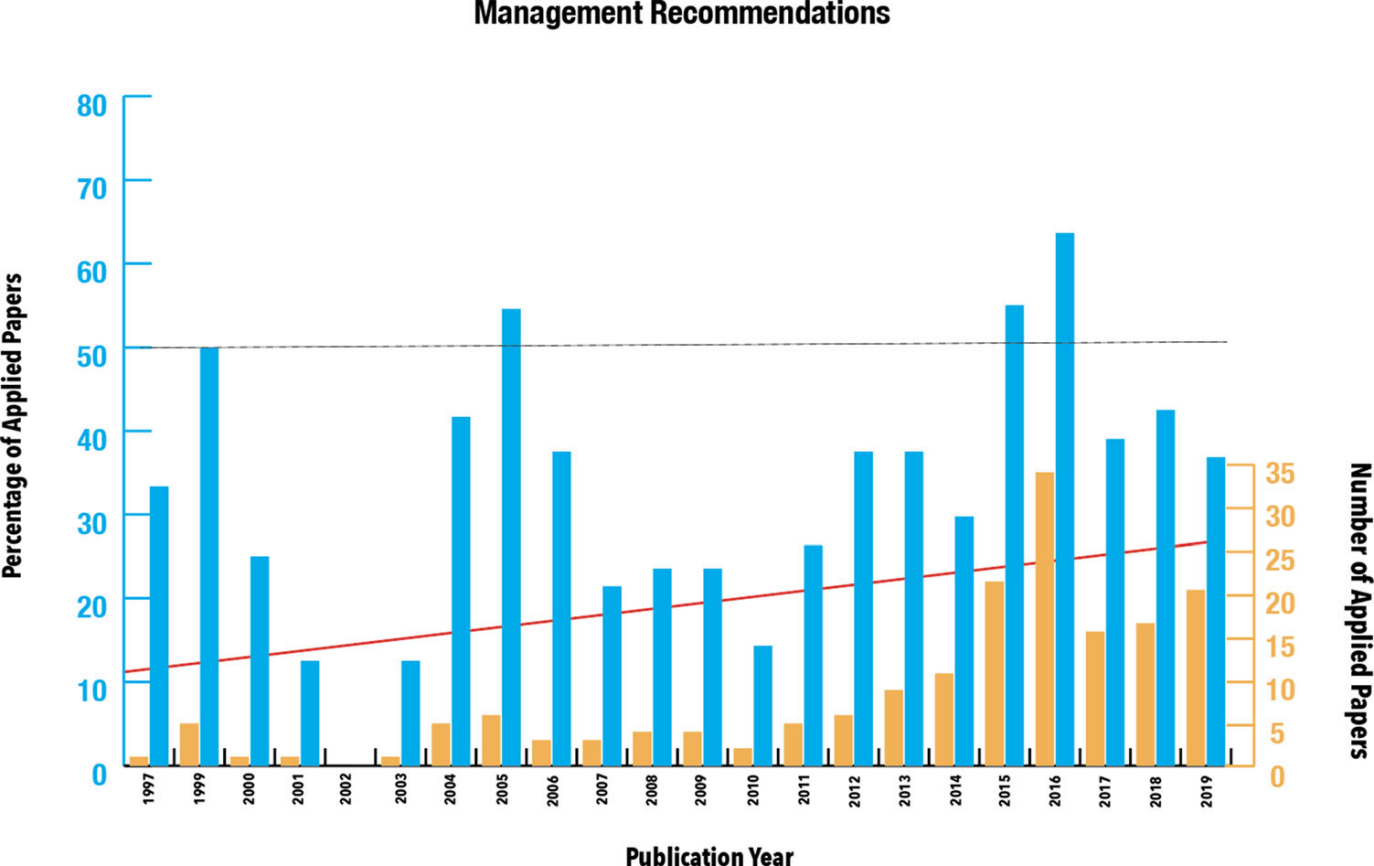
Number and percentage of papers with management recommendations per year with a linear trendline. Only years with more than two papers (after 1997) are shown

Leaving aside papers that did not mention the application of their results as well as those papers that included only a brief remark that the results could be useful, the number of papers that meaningfully engaged with the applied aspect of their data was 178 (37.8%). The number of such papers per year steadily increased, and, allowing for some fluctuations, so did their proportion in the overall production of historical-ecological works, hovering around 40% after 2012 with maximum values of over 60% (Fig. 6).

## Discussion

The widespread development of historical-ecological research in recent decades is an exciting but still poorly understood trend in contemporary scholarship. Our evaluation here confirmed that self-identifying historical ecologists use a wide variety of sources and methods and increasingly engage in the applied aspects of their research. This result is in line with our experience of an observable and palpable desire for researchers, students, and policy makers to make greater use of deep-time social and natural scientific datasets. In the following, we discuss the main research gaps and challenges for the future of historical ecology that emerge from our analysis of 544 scientific studies.

### Expanding geographical and linguistic reach

The historical-ecological research we analyzed focuses mainly on Europe and the USA. However, if historical ecology is to achieve global relevance and open up beyond the dominant Anglo-European perspective (cf. Kienast et al. 2021), efforts should be made to include more study regions and also to produce more cohesive global overviews. Related disciplines have made significant advances in this respect, which can serve as inspiration for historical ecology. For example, environmental archaeologists have long published cross-referential data and methods spanning multiple spatial and temporal scales (Dincauze 2000). Environmental history has recently become a global field with significant research activities in Asia, Latin America, Africa and even the polar regions (McNeill 2010; Howkins 2015). Furthermore, global environmental history (i.e., the environmental history of the entire Earth system) is also flourishing (McNeill and Mauldin 2015). One way to achieve better regional connectivity is with a more concerted application and analyses of comparative datasets and studies. A good example of such a study is Whitlock et al (2018), where the authors compared the land-use history at eight sites across four continents, along a gradient of landscape conditions from nearly pristine to highly altered, informing forest conservation strategies. However, our results show that comparative studies comprise only a small fraction of historical-ecological investigations. Sources used in historical ecology are rarely easily compatible through different temporal and spatial scales, especially among anthropologists/archaeologists where the focus for some researchers is on the interpretation of culture and environment, rather than on highly scalable syntheses like human adaptations or systems approaches (Armstrong et al. 2017). Nonetheless, comparative studies in environmental history (Hall 2005; Henderson et al. 2005) demonstrate that highly localized historical sources do not constitute an insurmountable obstacle to comparative research. It may well be that historical ecology, like environmental history, will move towards more comparative approaches, including global syntheses and narratives, as it matures over time. Recent years have indicated such a trend with increasing numbers of comparative studies published since 2011, culminating with 13 such works in 2019.

We acknowledge that our monolingual SCOPUS search likely produced specific and interrelated biases in the results (cf. Nuñez and Amano 2021). Peer-reviewed papers in English-language journals currently dominate scientific communication. Because of language and socioeconomic barriers (Clavero 2010), most scientific research is produced in the global north. For example, analyzing papers submitted to and published in the *Journal of Applied Ecology* in 2015–2017, Nuñez et al. (2019) arrived at a geographical pattern highly similar to our results. However, when efforts are made to avoid monolingual products and searches go beyond peer-reviewed journals, different patterns can emerge. For example, using Google Scholar, Amano et al. (2016) found that 35.6% of over 70 000 scientific documents on biodiversity conservation published in 2014 were written in languages other than English. A specificity of historical-ecological research is that authors belonging to the anthropological branch of historical ecology (Balée 2006; Armstrong et al. 2017) often publish their results (whether in English or not) in books, which, with the exception of book chapters that matched our keywords, were not included in our database (e.g., Odonne and Molino 2020; Whitaker et al. 2023). Exacerbating the geographical bias in our study, many of these same anthropological or non-English works tend to have broader regional foci, for example, representing research in Central America, South America, and Africa (Fairhead and Leach 1996; Ross and Rangel 2011; Rostain 2012; see also the handbook Isendahl and Stump 2019).

While we cannot possibly do justice to non-English historical-ecological literature in this paper, it is worth pointing out the rich legacy and the geographical diversity of studies in at least some major languages. French language contributions to historical ecology have been longstanding, with some researchers highlighting the role of people in shaping the structure of ecosystems in Central Africa (Letouzey 1968) or dealing explicitly with the stochastic character of ecosystems and calling for more concerted interdisciplinary research that would combine history and ecology (Drouin 1994). Almost 50 years ago, Barrau (1977) called for broader epistemological tinkering that would see the development of multi-disciplinary research concerning human–environment interactions through time. French language contributions to the field of historical ecology have done just that—there is global coverage of such research in Amazonia (Odonne and Molino 2018), Central Africa (Bogaert et al. 2020), Canada (Danneyrolles et al. 2020) and beyond. Spanish-language contributions include theoretical works (Garrido-Pérez et al. 2021) and research on diverse topics, such as transhumance pastoralism in Spain (López Sáez et al. 2009). As expected, many studies cover areas outside Europe and North-America. For example, López and Ospina (2008) edited a collection of 21 essays devoted to the historical ecology of South-America, especially Columbia. Other works focused on the intersection of biology, ecology, and Indigenous land-use across the Amazon (Cangussu et al. 2021) or historical water management in Mexico (Jaramillo Monroy et al. 2021). Tiapa (2010) studied the impacts of European agriculture and cattle grazing in the sixteenth-eighteenth centuries in the Orinoco delta in Venezuela, and Gassón and Heinen (2012) described ecological degradation and its effect on the distribution of local populations in the past century in the same area. Non-English contributions to the field of historical ecology are rich and diverse—biologists, historians, ecologists, and anthropologists should continue to make more effort to cross-reference and better utilize studies across languages, borders and worldviews.

### Maintaining methodological rigour

Our data confirm that historical ecology is fundamentally an interdisciplinary field with almost all studies using methodological approaches spanning the life sciences, social sciences, and humanities. This lateral integration of source data and methods, despite their possibly disparate audiences and backgrounds, greatly benefits our collective desire to better understand human–environment interactions through time (Rick and Lockwood 2013; Santana-Cordero et al. 2014). However, to ensure the rigorous use of data, source diversity should continue to develop in a framework where methodological diversity is also highly scrutinized (Coughlan and Nelson 2019; Monsarrat et al. 2019). Quantitative analyses (e.g., use of statistical and modelling approaches) dominated the combinations of methods in our review, which signals that historical ecology—at least as understood in the papers we analyzed—is firmly embedded in natural scientific frameworks with a critical scrutiny of how the data are gathered and inferences are made. At the same time, social scientific (including historical) methodologies, while a significant player in the papers reviewed, are not always scrutinized to the same extent. For historical ecology to reach its full potential as a truly integrative discipline, these disparities need to be addressed. Here we showcase a few examples of how researchers from the natural sciences can uphold the same high standards they adhere to in the application of social scientific data and inferences.

The basic principle historians apply to their sources is a formal critical approach to the information contained in them. This “source criticism” has many meanings (Dobson and Ziemann 2009), but in essence it orders that information from historical sources must not be simply accepted at face value and must examined with reference to how it was produced (e.g., social, political, gendered, and racial contexts). In historical ecology, the use of historical sources without the application of a critical approach may lead to biased or false conclusions. For example, using a geographical lexicon, Nores and López-Bao (2022) estimated that the current distribution of the Iberian wolf (*Canis lupus signatus*) still covers 70% of its 19th-century distribution. In contrast, Clavero et al. (2022) used the same dataset but applied predictive modelling to compensate for gaps and biases in the data and arrived at a radically different result (30% of 19th-century distribution still intact). Corti and Díaz (2022) criticized Flueck et al. (2022) for uncritically using historical sources thereby arriving at faulty conclusions regarding the former distribution, abundance, and habitat preferences of the huemul deer (*Hippocamelus bisulcus*) in Chile and Argentina. Such differences can have crucial implications for species protection and restoration. In extreme cases, a lack of understanding of history as an inveterate and iterative methodology has also led to some researchers dismissing it altogether. Clavero and Centeno-Cuadros (2016) criticized Matallanas et al. (2016) for asserting that historical documents are unreliable and therefore inferior to genetic information concerning the history of the crayfish *Austropotamobius italicus* in Spain.

Some authors have already addressed the issue of critically assessing historical sources in ecological and biological studies (e.g., Bürgi et al. 2010), but, in general, there seems to be little awareness of this issue. This may stem from the fact that many of the authors contributing to historical ecology are natural scientists with little formal training in historical methods. Pooley (2018) advised historical ecologists “not to be naive about the challenges of historical data” and provided practical guidelines as to how to proceed carefully and critically with their application. Similar works have been published by Rymer (1979) and Forman and Russell (1983). We encourage our colleagues from the life sciences to engage with the issues raised here. At the same time, we recognize that specialization is increasing in all disciplines, and “scientists should not be expected to become historians” (Pooley 2013). A stronger and more lateral integration of interdisciplinary teams, with emphasis on the inclusion of social scientists (historians, anthropologists) will undoubtedly lead to more rigorous, diverse, and interesting research processes and outcomes (Szabó and Hédl 2013; Armstrong et al. 2017).

### Integrating knowledge at different spatial and temporal scales

There was a relative lack of explicit information regarding the spatial and temporal scales in the papers we analyzed—only 148 and 204 studies, respectively, included explicit information on the time and region of study. We see this as a deficiency of historical ecology and encourage authors to be more explicit in defining their study areas and periods. No significant trends for a typical spatial scale of investigation emerged, despite some practitioners advocating for a landscape-scale approach (Crumley 2017). Papers reviewed in this study indicated that the spatial scale of inquiry was evenly distributed, ranging from small scale (< 100 ha) case studies to larger (> 1 000 000 ha) syntheses. As to the temporal range of studies, we observed that study periods of up to a few centuries were the most common. Given that written documents dominated among the sources used in the papers, we conclude that the preference for relatively short time-frames (a few centuries) was probably determined by the fact that written documents are usually not available for longer periods.

Of the papers reviewed, anthropological and archaeological studies typically favoured longer temporal ranges (centennial and millennial scales). However, source materials are typically more scant and less reliable the further we go back in time, and the resolution of data risks being more skewed when analyses cover broad (millennial) time scales. We argue that despite these limitations, the appropriate temporal scale for any phenomenon is determined by that phenomenon itself. As Lane (2019) noted, “the rates at which different processes operate, and hence the temporal span required for their effective analysis, vary.” The purpose of the analysis also matters. For example, to analyze changes in forest vegetation following the cessation of traditional management in the twentieth century, a decadal scale is suitable (Müllerová et al. 2015; Armstrong et al. 2022). However, if the aim of the analysis is to understand the evolution and functioning of the same forest as a socio-ecological system (for example to mitigate the negative effects of management cessation), centennial or rather millennial scale inquiry may be better suited (Fitzpatrick and Keegan 2007). Furthermore, in many parts of the world, especially in settler colonial nations, historical written sources are relatively scant, or are produced through problematic colonial gazes (e.g. Geniusz 2022). In such cases, ecological studies which focus on cultural drivers as well as biophysical processes over longer time periods represent an important pathway for critically interpreting environmental change and long-term human management legacies, often applied to mitigating climate change impacts and managing natural resources (Hoffman et al. 2021). Finally, centennial and millennial scale research provides for more general patterns to be uncovered, patterns that can be flagged, replicated, or rejected across other geographic spheres and cultures (Brondízio et al. 2021). This would also foster more effective comparative research, which, as pointed out above, is one of the areas with the greatest potential for future historical-ecological studies.

Emphasizing the need for synergetic research across disciplines is of course not new. Indeed, there have been repeated calls to integrate paleobiology and archaeology into historical ecology (e.g., Rick and Lockwood 2013), and to “develop a unified framework for understanding temporal change in complex social-environmental systems” (Beller et al. 2017). There are a number of initiatives that tackle such issues. For example, the Integrated History and Future of People on Earth (IHOPE) network brings together researchers to study past human impacts on the Earth system. The Oceans Past Initiative is a global research network for historical research investigating humankind’s interaction with global marine life over millennia. Although clearly not all research can cover extensive spatial and temporal scales, we see such integrative frameworks as the most exciting opportunity to further develop historical-ecological research.

### Applying results in environmental conservation/restoration

Management and policy recommendations have been perceived as an integral part of historical ecology since the beginnings of the discipline in 1960s Europe (Szabó 2015). Later examples followed, with more targeted efforts to understand the role of historical ecology in contemporary conservation and restoration. For example, Swetnam et al. (1999) described the advantages and constraints of applying historical-ecological knowledge in landscape management and restoration; and Egan and Howell (2001) subtitled their *Historical Ecology Handbook*: A Restorationist’s guide to reference ecosystems. To find out what policy recommendations have emerged from historical-ecological research, Beller et al. (2020) analysed 217 papers published between 1994 and 2017. According to their results, the most prevalent recommendations concerned the conservation/restoration of native species and ecosystems, active management practices, and increasing connectivity. Although they did not explicitly address the temporal distribution of the papers analysed, there was an increasing trend with many papers, especially after 2012, purported to contribute to these applied aims. Our dataset showed a similar overall picture, with a marked increase in the percentage of papers containing applied recommendations. This clearly signals the extent to which historical ecologists have become concerned with the potential impact of their research in real-life situations. Pertinent to this trend are growing requirements among funding agencies that researchers make explicit the applicability of their research programme. For example, large federal agencies in North America require explicit knowledge mobilization strategies for all research projects.

Perhaps as a reaction to the increase in the applied research scope of historical-ecological practitioners, Stockdale et al. (2019a) emphasized that focusing on the core goals of historical ecology still has intrinsic value and that “ecological restoration does not need to be the end goal to make historical analyses worthwhile”. At the other end of the spectrum, Pape (2022) argued that restoration ecology should look towards the future instead of the past and focus on environmental justice instead of reference conditions. While we agree that environmental justice needs to be a part of all restoration and management projects, we are also convinced that historical ecology can do much more than establishing reference conditions, and, especially its anthropological branch, is very well placed to tackle exactly such issues (cf. Armstrong and Veteto 2015).

As our results show, management and policy recommendations are now a relatively integral part of standard historical-ecological practice and we see the application of these recommendations as the most important challenge for the future. First, to assess whether historical ecology has any real-life impact (whether recommendations are in fact implemented), more knowledge-mobilization is likely needed. A good example of this is the long-term monitoring of the reintroduction of traditional management into European forests, where the results for biodiversity conservation are positive and in line with expectations, but important differences between management types as well as the effects of recent global change need to be factored in (e.g. Vild et al. 2013). Second, innovative research directions should focus on testing the future effect-scenarios from management strategies based on historical knowledge. For example, Stockdale et al. (2019b) modelled whether restoring historical vegetation conditions would change the fire dynamics in the Rocky Mountains of Alberta, Canada.

## Conclusions

This paper provides an assessment of the sources and methods used, and types of studies practiced in historical ecology as well as of the integration of management recommendations into the discipline. We analyzed papers that identified their research as historical ecology. While this introduced a level of objectivity into our selection and kept the database at a feasible size, it excluded non-self-identifying historical ecology, and we note that the results should be interpreted against this background. Our results confirm previous assumptions about the methodological diversity of historical ecology and highlight areas where advances could be made. Although historical written documents dominated the sources used in our review, the methods were predominantly quantitative. This may illustrate why there continues to be apprehension (and misuse) of historical and ethnographic records among practitioners in biology, ecology, and other life science disciplines. Applying statistical analyses and modelling with historical sources that have not been critically sourced and analyzed is a challenge that must be overcome. We encourage ecologists and biologists to engage with these issues and we also encourage interdisciplinary cooperation through the “great divide” between the natural and social sciences.

Historical ecology is dominated by European and North American scholars, and our results underline the biases inherent in monolingual searches of commercial abstract and citation databases, such as SCOPUS—it is clear that including non-English language studies, grey literature, and books would reduce the geographical imbalances apparent in our results. However, these data also highlight the potential in (and need for) more diverse regional foci and more inclusive recognition of researchers outside English-speaking contexts. In doing so, more comparative and critical approaches will emerge, increasing the global reach of this growing field.

Our data showed a general lack of explicit and comprehensive information or critical scrutiny of the spatial and temporal scales within which research is conducted. More concerted and reflective efforts should be made to grapple with and explore how we apply and define scalar boundaries in our works. Historical-ecological studies span many spatial and temporal scales, and we found a tendency for existing research to cluster around periods of < 500 years. Although this pattern is likely determined by the availability of historical written documents, we argue that fostering longer-term studies covering millennial scales can help historical ecology realize its full potential.

Lastly, according to our results, management and policy recommendations are now a relatively standard part of historical ecology. We suggest that as the next step for increasing real-world impacts and knowledge mobilization, practitioners ought to examine whether and to what extent their research recommendations have been followed or implemented. At the same time, researchers should make concerted efforts to predict the effects of management changes based on recommendations by historical ecologists. This way, the discipline can better contribute solutions to the existential challenges facing humanity in an uncertain future.

### Supplementary Information

Below is the link to the electronic supplementary material.

Supplementary file1 (PDF 866 KB)
