# Supplementary material for: The practice of historical ecology: What, when, where, how and what for
Source: Ambio. 2024 Mar 5;53(5):664–77. doi: 10.1007/s13280-024-01981-1 (PMC10992833; doi:10.1007/s13280-024-01981-1)
Supplement: Supplementary file 1 — Supplementary file1 (PDF 866 KB) [file 13280_2024_1981_MOESM1_ESM.pdf]

Ambio

Electronic Supplementary Material
This supplementary material has not been peer reviewed.

Title: **The practice of historical ecology: What, when, where, how and what for**

Authors: Aarón M. Santana-Cordero, Péter Szabó, Matthias Bürgi, Chelsey Geralda Armstrong

Scopus
EXPORT DATE: 03 Aug 2020

2019

1. Monsarrat, S., Novellie, P., Rushworth, I., Kerley, G.
Shifted distribution baselines: Neglecting long-term biodiversity records risks overlooking potentially suitable habitat for conservation management
(2019) Philosophical Transactions of the Royal Society B: Biological Sciences, 374 (1788), art. no. 20190215, . Cited 3 times.

DOI: 10.1098/rstb.2019.0215

2. Johnson LB, Margolis EQ (2019) Surface fire to crown fire: Fire history in the taos valley watersheds, new mexico, USA. Fire Volume 2, Issue 1, Pages 1 - 21.

DOI: 10.3390/fire2010014

3. McMahon, K.W., Michelson, C.I., Hart, T., McCarthy, M.D., Patterson, W.P., Polito, M.J.
Divergent trophic responses of sympatric penguin species to historic anthropogenic exploitation and recent climate change
(2019) Proceedings of the National Academy of Sciences of the United States of America, 116 (51), pp. 25721-25727.

DOI: 10.1073/pnas.1913093116

4. Rick, T., Harvey, V.L., Buckley, M.
Collagen fingerprinting and the Chumash billfish fishery, Santa Barbara Channel, California, USA
(2019) Archaeological and Anthropological Sciences, 11 (12), pp. 6639-6648.

DOI: 10.1007/s12520-019-00930-4

5. Hentati-Sundberg, J., Hellquist, K.F., Duit, A.
Iron triangles and subsidies: Understanding the long-term role of the government on Swedish commercial fisheries
(2019) Ecology and Society, 24 (4), art. no. 18, .

DOI: 10.5751/ES-11259-240418

6. Berg, S., Zhu, J.U.N., Clayton, M.K., Shea, M.E., Mladenoff, D.J.
A latent discrete markov random field approach to identifying and classifying historical forest communities based on spatial multivariate tree species counts
(2019) Annals of Applied Statistics, 13 (4), pp. 2312-2340. Cited 1 time.

DOI: 10.1214/19-AOAS1259

7. Dawson, A., Paciorek, C.J., Goring, S.J., Jackson, S.T., McLachlan, J.S., Williams, J.W.
Quantifying trends and uncertainty in prehistoric forest composition in the upper Midwestern United States

(2019) Ecology, 100 (12), art. no. e02856, .

DOI: 10.1002/ecy.2856

8. Auffret, A.G., Thomas, C.D.
Synergistic and antagonistic effects of land use and non-native species on community responses to climate change
(2019) Global Change Biology, 25 (12), pp. 4303-4314. Cited 2 times.

DOI: 10.1111/gcb.14765

9. Zapelini, C., Bender, M.G., Giglio, V.J., Schiavetti, A.
Tracking interactions: Shifting baseline and fisheries networks in the largest Southwestern Atlantic reef system
(2019) Aquatic Conservation: Marine and Freshwater Ecosystems, 29 (12), pp. 2092-2106. Cited 4 times.

DOI: 10.1002/aqc.3224

10. Clavero, M.
Beyond one bone: Interdisciplinarity to assess nativeness of the tench (Tinca tinca) in Spain
(2019) Aquatic Conservation: Marine and Freshwater Ecosystems, 29 (11), pp. 1863-1869. Cited 1 time.

DOI: 10.1002/aqc.3184

11. McClenachan, L., Grabowski, J.H., Marra, M., McKeon, C.S., Neal, B.P., Record, N.R., Scyphers, S.B.
Shifting perceptions of rapid temperature changes’ effects on marine fisheries, 1945–2017
(2019) Fish and Fisheries, 20 (6), pp. 1111-1123.

DOI: 10.1111/faf.12400

12. Price, M.H.H., Connors, B.M., Candy, J.R., McIntosh, B., Beacham, T.D., Moore, J.W., Reynolds, J.D.
Genetics of century-old fish scales reveal population patterns of decline
(2019) Conservation Letters, 12 (6), art. no. e12669, . Cited 2 times.

DOI: 10.1111/conl.12669

13. Toniello, G., Lepofsky, D., Lertzman-Lepofsky, G., Salomon, A.K., Rowell, K.
11,500 y of human-clam relationships provide longterm context for intertidal management in the Salish Sea, British Columbia
(2019) Proceedings of the National Academy of Sciences of the United States of America, 116 (44), pp. 22106-22114. Cited 3 times.

DOI: 10.1073/pnas.1905921116

14. Cavanaugh, K.C., Dangremond, E.M., Doughty, C.L., Park Williams, A., Parker, J.D., Hayes, M.A., Rodriguez, W., Feller, I.C.
Climate-driven regime shifts in a mangrove–salt marsh ecotone over the past 250 years
(2019) Proceedings of the National Academy of Sciences of the United States of America, 116 (43), pp. 21602-21608. Cited 10 times.

DOI: 10.1073/pnas.1902181116

15. McCain, J., Braje, T.J., Hernández Estrada, R.L., Michelini, A.P., Aguilar, J., Rick, T.C.
The Four Crowns of the Sea: Archaeological Reconnaissance of the Coronados Islands, Baja California, México
(2019) Journal of Island and Coastal Archaeology, 14 (4), pp. 541-559. Cited 1 time.

DOI: 10.1080/15564894.2018.1536901

16. Odonne, G., van den Bel, M., Burst, M., Brunaux, O., Bruno, M., Dambrine, E., Davy, D., Desprez, M., Engel, J., Ferry, B., Freycon, V., Grenand, P., Jérémie, S., Mestre, M., Molino, J.-F., Petronelli, P., Sabatier, D., Hérault, B.
Long-term influence of early human occupations on current forests of the Guiana Shield
(2019) Ecology, 100 (10), art. no. e02806, . Cited 7 times.

DOI: 10.1002/ecy.2806

17. Samojlik, T., Fedotova, A., Borowik, T., Kowalczyk, R.
Historical data on European bison management in Białowieża Primeval Forest can contribute to a better contemporary conservation of the species
(2019) Mammal Research, 64 (4), pp. 543-557. Cited 2 times.

DOI: 10.1007/s13364-019-00437-2

18. Finderup Nielsen, T., Sand-Jensen, K., Dornelas, M., Bruun, H.H.
More is less: net gain in species richness, but biotic homogenization over 140 years
(2019) Ecology Letters, 22 (10), pp. 1650-1657. Cited 8 times.

DOI: 10.1111/ele.13361

19. Maher, L.A.
Persistent Place-Making in Prehistory: the Creation, Maintenance, and Transformation of an Epipalaeolithic Landscape
(2019) Journal of Archaeological Method and Theory, 26 (3), pp. 998-1083. Cited 2 times.

DOI: 10.1007/s10816-018-9403-1

20. Crees, J.J., Turvey, S.T., Freeman, R., Carbone, C.
Mammalian tolerance to humans is predicted by body mass: evidence from long-term archives
(2019) Ecology, 100 (9), art. no. e02783, .

DOI: 10.1002/ecy.2783

21. Wright, D.K.
Long-term dynamics of pastoral ecology in northern Kenya: An old model for new resilience
(2019) Journal of Anthropological Archaeology, 55, art. no. 101068, . Cited 2 times.

DOI: 10.1016/j.jaa.2019.101068

22. Santana-Cordero, A.M., Szabó, P.
Exploring Qualitative Methods of Historical Ecology and Their Links With Qualitative Research
(2019) International Journal of Qualitative Methods, 18, .

DOI: 10.1177/1609406919872112

23. Rick, T., Braje, T., Wake, T., Sanchez, G., DeLong, R., Lightfoot, K.
Seventy Years of Archaeological Research on California’s Farallon Islands
(2019) California Archaeology, 11 (2), pp. 183-203. Cited 1 time.

DOI: 10.1080/1947461X.2019.1652043

24. Jarman, R., Chambers, F.M., Webb, J.
Landscapes of sweet chestnut (Castanea sativa) in Britain — their ancient origins
(2019) Landscape History, 40 (2), pp. 5-40.

DOI: 10.1080/01433768.2020.1676040

25. Stark, S., Egelkraut, D., Aronsson, K.-Å., Olofsson, J.
Contrasting vegetation states do not diverge in soil organic matter storage: evidence from historical sites in tundra
(2019) Ecology, 100 (7), art. no. e02731, . Cited 3 times.

DOI: 10.1002/ecy.2731

26. Coughlan, M.R., Nelson, D.R.
Geostatistical analysis of historical contingency and land use footprints in the prehistoric settlement dynamics of the South Carolina Piedmont, North America
(2019) Journal of Archaeological Science, 107, pp. 1-9.

DOI: 10.1016/j.jas.2019.04.003

27. Earnshaw, J.K.
Cultural Forests in Cross Section: Clear-Cuts Reveal 1,100 Years of Bark Harvesting on Vancouver Island, British Columbia
(2019) American Antiquity, 84 (3), pp. 516-530. Cited 1 time.

DOI: 10.1017/aaq.2019.29

28. Boles, O.J.C., Shoemaker, A., Courtney Mustaphi, C.J., Petek, N., Ekblom, A., Lane, P.J.
Historical Ecologies of Pastoralist Overgrazing in Kenya: Long-Term Perspectives on Cause and Effect
(2019) Human Ecology, 47 (3), pp. 419-434. Cited 2 times.

DOI: 10.1007/s10745-019-0072-9

29. Rick, T.C., Braje, T.J., Erlandson, J.M.
Early red abalone shell middens, human subsistence, and environmental change on California's Northern channel Islands
(2019c) Journal of Ethnobiology, 39 (2), pp. 204-222.

DOI: 10.2993/0278-0771-39.2.204

30. Barrett, J.H.

An environmental (pre)history of European fishing: past and future archaeological contributions to sustainable fisheries
(2019) Journal of Fish Biology, 94 (6), pp. 1033-1044. Cited 4 times.

DOI: 10.1111/jfb.13929

31. Stockdale, C.A., Macdonald, S.E., Higgs, E.
Forest closure and encroachment at the grassland interface: a century-scale analysis using oblique repeat photography
(2019) Ecosphere, 10 (6), art. no. e02774, . Cited 1 time.

DOI: 10.1002/ecs2.2774

32. Gayo, E.M., McRostie, V.B., Campbell, R., Flores, C., Maldonado, A., Uribe-Rodriguez, M., Moreno, P.I., Santoro, C.M., Christie, D.A., Muñoz, A.A., Gallardo, L.
Geohistorical records of the Anthropocene in Chile
(2019) Elementa, 7 (1), art. no. 15, . Cited 3 times.

DOI: 10.1525/elementa.353

33. Carnell, P.E., Keough, M.J.
Reconstructing Historical Marine Populations Reveals Major Decline of a Kelp Forest Ecosystem in Australia
(2019) Estuaries and Coasts, 42 (3), pp. 765-778. Cited 13 times.

DOI: 10.1007/s12237-019-00525-1

34. Gray, M., Ermgassen, P., Gair, J., Langdon, C., Lemagie, E., Lerczak, J.
Spatially Explicit Estimates of In Situ Filtration by Native Oysters to Augment Ecosystem Services during Restoration
(2019) Estuaries and Coasts, 42 (3), pp. 792-805. Cited 2 times.

DOI: 10.1007/s12237-019-00515-3

35. Litza, K., Diekmann, M.
Hedgerow age affects the species richness of herbaceous forest plants
(2019) Journal of Vegetation Science, 30 (3), pp. 553-563. Cited 4 times.

DOI: 10.1111/jvs.12744

36. Becker-Scarpitta, A., Vissault, S., Vellend, M.
Four decades of plant community change along a continental gradient of warming
(2019) Global Change Biology, 25 (5), pp. 1629-1641. Cited 4 times.

DOI: 10.1111/gcb.14568

37. Giovas, C.M.
The Beasts at Large–Perennial Questions and New Paradigms for Caribbean Translocation Research. Part I: Ethnozoogeography of Mammals
(2019) Environmental Archaeology, 24 (2), pp. 182-198. Cited 12 times.

DOI: 10.1080/14614103.2017.1315208

38. Haas, H., Braje, T.J., Edwards, M.S., Erlandson, J.M., Whitaker, S.G.

Black abalone (Haliotis cracherodii) population structure shifts through deep time: Management implications for southern California's northern Channel Islands
(2019) Ecology and Evolution, 9 (8), pp. 4720-4732. Cited 1 time.

DOI: 10.1002/ece3.5075

39. Douglass, K., Walz, J., Quintana Morales, E., Marcus, R., Myers, G., Pollini, J.
Historical perspectives on contemporary human–environment dynamics in southeast Africa
(2019) Conservation Biology, 33 (2), pp. 260-274. Cited 7 times.

DOI: 10.1111/cobi.13244

40. Guerrero-Gatica, M., Aliste, E., Simonetti, J.A.
Shifting gears for the use of the shifting baseline syndrome in ecological restoration
(2019) Sustainability (Switzerland), 11 (5), art. no. 1458, . Cited 3 times.

DOI: 10.3390/su11051458

41. dos Reis-Neto, A.S., Meireles, A.J.A., Cunha-Lignon, M.
Natural regeneration of the mangrove vegetation on abandoned salt ponds in Ceará, in the semi-arid region of northeastern Brazil
(2019) Diversity, 11 (2), art. no. 27, . Cited 2 times.

DOI: 10.3390/d11020027

42. Vokhshoori, N.L., McCarthy, M.D., Collins, P.W., Etnier, M.A., Rick, T., Eda, M., Beck, J., Newsome, S.D.
Broader foraging range of ancient short-tailed albatross populations into California coastal waters based on bulk tissue and amino acid isotope analysis
(2019) Marine Ecology Progress Series, 610, pp. 1-13. Cited 4 times.

DOI: 10.3354/meps12839

43. Fitzhugh, B., Butler, V.L., Bovy, K.M., Etnier, M.A.
Human ecodynamics: A perspective for the study of long-term change in socioecological systems
(2019) Journal of Archaeological Science: Reports, 23, pp. 1077-1094. Cited 17 times.

DOI: 10.1016/j.jasrep.2018.03.016

44. Cammen, K.M., Rasher, D.B., Steneck, R.S.
Predator recovery, shifting baselines, and the adaptive management challenges they create
(2019) Ecosphere, 10 (2), art. no. e02579, .

DOI: 10.1002/ecs2.2579

45. Stockdale, C.A., McLoughlin, N., Flannigan, M., Macdonald, S.E.
Could restoration of a landscape to a pre-European historical vegetation condition reduce burn probability?
(2019) Ecosphere, 10 (2), art. no. e02584, . Cited 4 times.

DOI: 10.1002/ecs2.2584

46. Seeley, M., Goring, S., Williams, J.W.
Assessing the environmental and dispersal controls on Fagus grandifolia distributions in the Great Lakes region
(2019) Journal of Biogeography, 46 (2), pp. 405-419. Cited 1 time.

DOI: 10.1111/jbi.13491

47. Bond, A.L., Carlson, C.J., Burgio, K.R.
Local extinctions of insular avifauna on the most remote inhabited island in the world
(2019) Journal of Ornithology, 160 (1), pp. 49-60. Cited 1 time.

DOI: 10.1007/s10336-018-1590-8

48. da Silva, T.C., Campos, L.Z.O., Balée, W., Medeiros, M.F.T., Peroni, N., Albuquerque, U.P.
Human impact on the abundance of useful species in a protected area of the Brazilian Cerrado by people perception and biological data
(2019) Landscape Research, 44 (1), pp. 75-87. Cited 4 times.

DOI: 10.1080/01426397.2017.1396304

49. Troufflard, J., Alves, D.T.
An interdisciplinary approach to the Cedro archaeological site, Lower Amazon
(2019) Boletim do Museu Paraense Emilio Goeldi:Ciencias Humanas, 14 (2), pp. 553-580.

DOI: 10.1590/1981.81222019000200015

50. Moret, P., Muriel, P., Jaramillo, R., Dangles, O.
Humboldt’s Tableau Physique revisited
(2019) Proceedings of the National Academy of Sciences of the United States of America, 116 (26), pp. 12889-12894. Cited 8 times.

DOI: 10.1073/pnas.1904585116

51. Bedford, J., Johns, D., McQuatters-Gollop, A.
A century of change in North Sea plankton communities explored through integrating historical datasets
(2019) ICES Journal of Marine Science, 76 (1), pp. 104-112. Cited 1 time.

DOI: 10.1093/icesjms/fsy148

52. Monsarrat, S., Boshoff, A.F., Kerley, G.I.H.
Accessibility maps as a tool to predict sampling bias in historical biodiversity occurrence records
(2019b) Ecography, 42 (1), pp. 125-136. Cited 6 times.

DOI: 10.1111/ecog.03944

53. Giovas, C.M., Kappers, M., Lowe, K.M., Termes, L.
The Carriacou Ecodynamics Archaeology Project: First Results of Geophysical Survey and Landscape Archaeology at the Sabazan Site, The Grenadines
(2019) Journal of Island and Coastal Archaeology, .

DOI: 10.1080/15564894.2019.1642969

54. Pescini, V.
Which origin for charcoal in soils? Case-studies of Environmental Resources Archaeology (ERA) From the Ligurian Apennines, seventh to the Twentieth century
(2019) Frontiers in Environmental Science, 7 (JUN), art. no. 77, .

DOI: 10.3389/fenvs.2019.00077

55. McKey, D.
Pre-Columbian human occupation of Amazonia and its influence on current landscapes and biodiversity
(2019) Anais da Academia Brasileira de Ciencias, 91, art. no. e20190087, .

DOI: 10.1590/0001-3765201920190087

56. Pickart, A.J., Hesp, P.A.
Spatio-temporal geomorphological and ecological evolution of a transgressive dunefield system, Northern California, USA
(2019) Global and Planetary Change, 172, pp. 88-103. Cited 3 times.

DOI: 10.1016/j.gloplacha.2018.09.012

57. Maes, S.L., Perring, M.P., Vanhellemont, M., Depauw, L., Van den Bulcke, J., Brūmelis, G., Brunet, J., Decocq, G., den Ouden, J., Härdtle, W., Hédl, R., Heinken, T., Heinrichs, S., Jaroszewicz, B., Kopecký, M., Máliš, F., Wulf, M., Verheyen, K.
Environmental drivers interactively affect individual tree growth across temperate European forests
(2019) Global Change Biology, 25 (1), pp. 201-217. Cited 11 times.

DOI: 10.1111/gcb.14493

58. Howard, I., Davis, E., Lippert, G., Quinn, T.P., Wood, C.L.
Abundance of an economically important nematode parasite increased in Puget Sound between 1930 and 2016: Evidence from museum specimens confirms historical data
(2019) Journal of Applied Ecology, 56 (1), pp. 190-200. Cited 3 times.

DOI: 10.1111/1365-2664.13264

59. Mackenzie, C.M., Mittelhauser, G., Miller-Rushing, A.J., Primack, R.B.
Floristic change in New England and New York: Regional patterns of plant species loss and decline
(2019) Rhodora, 121 (985), pp. 1-36. Cited 1 time.

DOI: 10.3119/18-04

2018

60. Coughlan MR, Magi BI, Derr KM (2018) A global analysis of hunter-gatherers, broadcast fire use, and lightning-fire-prone landscapes. Fire 1, Issue 3, Pages 1 - 13. Art. number 41.

DOI: 10.3390/fire1030041

61. Fisher, C.
Towards a dialogue of sustainable agriculture and end-times theology in the United States: insights from the historical ecology of nineteenth century millennial communes
(2018) Agriculture and Human Values, 35 (4), pp. 791-807. Cited 2 times.

DOI: 10.1007/s10460-018-9874-4

62. Farahani, A.
A 2500-year historical ecology of agricultural production under empire in Dhiban, Jordan
(2018) Journal of Anthropological Archaeology, 52, pp. 137-155. Cited 1 time.

DOI: 10.1016/j.jaa.2018.09.006

63. Iles, L., Stump, D., Heckmann, M., Lang, C., Lane, P.J.
Iron Production in North Pare, Tanzania: Archaeometallurgical and Geoarchaeological Perspectives on Landscape Change
(2018) African Archaeological Review, 35 (4), pp. 507-530. Cited 2 times.

DOI: 10.1007/s10437-018-9312-4

64. Schnedl, S.-M., Haselmair, A., Gallmetzer, I., Mautner, A.-K., Tomašových, A., Zuschin, M.
Molluscan benthic communities at Brijuni Islands (northern Adriatic Sea) shaped by Holocene sea-level rise and recent human eutrophication and pollution
(2018) Holocene, 28 (11), pp. 1801-1817. Cited 2 times.

DOI: 10.1177/0959683618788651

65. Abadie, J., Avon, C., Dupouey, J.-L., Lopez, J.-M., Tatoni, T., Bergès, L.
Land use legacies on forest understory vegetation and soils in the Mediterranean region: Should we use historical maps or in situ land use remnants?
(2018) Forest Ecology and Management, 427, pp. 17-25. Cited 3 times.

DOI: 10.1016/j.foreco.2018.05.050

66. Lambrides, A.B.J., Weisler, M.I.
Late Holocene Marshall Islands Archaeological Tuna Records Provide Proxy Evidence for ENSO Variability in the Western and Central Pacific Ocean
(2018) Journal of Island and Coastal Archaeology, 13 (4), pp. 531-562. Cited 6 times.

DOI: 10.1080/15564894.2017.1315350

67. Riede, F., Høye, T.T., Tejsner, P., Veldhuis, D., Willerslev, R.
Special Section Introduction: Socioecological Disequilibrium in the Circumpolar North
(2018) Human Ecology, 46 (5), pp. 615-620. Cited 2 times.

DOI: 10.1007/s10745-018-0009-8

68. Schaeffer-Novelli, Y., Cintrón-Molero, G., Reis-Neto, A.S., Abuchahla, G.M.O., Neta, L.C.P., Lira-Medeiros, C.F.
The mangroves of Araçá Bay through time: An interdisciplinary approach for conservation of spatial diversity at large scale
(2018) Ocean and Coastal Management, 164, pp. 60-67. Cited 3 times.

DOI: 10.1016/j.ocecoaman.2017.12.024

69. Cosentino, B.J., Brubaker, K.M.
Effects of land use legacies and habitat fragmentation on salamander abundance
(2018) Landscape Ecology, 33 (9), pp. 1573-1584. Cited 3 times.

DOI: 10.1007/s10980-018-0686-0

70. Thurstan, R.H., Buckley, S.M., Pandolfi, J.M.
Trends and transitions observed in an iconic recreational fishery across 140 years
(2018) Global Environmental Change, 52, pp. 22-36. Cited 1 time.

DOI: 10.1016/j.gloenvcha.2018.06.002

71. Keane, R.E., Loehman, R.A., Holsinger, L.M., Falk, D.A., Higuera, P., Hood, S.M., Hessburg, P.F.
Use of landscape simulation modeling to quantify resilience for ecological applications
(2018) Ecosphere, 9 (9), art. no. e02414, . Cited 11 times.

DOI: 10.1002/ecs2.2414

72. Garcia-Lozano, C., Pintó, J., Daunis-i-Estadella, P.
Changes in coastal dune systems on the Catalan shoreline (Spain, NW Mediterranean Sea). Comparing dune landscapes between 1890 and 1960 with their current status
(2018) Estuarine, Coastal and Shelf Science, 208, pp. 235-247. Cited 10 times.

DOI: 10.1016/j.ecss.2018.05.004

73. Brewer, J., Riede, F.
Cultural heritage and climate adaptation: a cultural evolutionary perspective for the Anthropocene
(2018) World Archaeology, 50 (4), pp. 554-569. Cited 3 times.

DOI: 10.1080/00438243.2018.1527246

74. Armstrong, C.G., Dixon, W.M., Turner, N.J.
Management and Traditional Production of Beaked Hazelnut (k'áp'xw-az', Corylus cornuta; Betulaceae) in British Columbia
(2018) Human Ecology, 46 (4), pp. 547-559. Cited 4 times.

DOI: 10.1007/s10745-018-0015-x

75. Belliard, J., Beslagic, S., Delaigue, O., Tales, E.
Reconstructing long-term trajectories of fish assemblages using historical data: the Seine River basin (France) during the last two centuries
(2018) Environmental Science and Pollution Research, 25 (24), pp. 23430-23450. Cited 3 times.

DOI: 10.1007/s11356-016-7095-1

76. Szabó, P., Suchánková, S., Křížová, L., Kotačka, M., Kvardová, M., Macek, M., Müllerová, J., Brázdil, R.
More than trees: The challenges of creating a geodatabase to capture the complexity of forest history
(2018) Historical Methods, 51 (3), pp. 175-189.

DOI: 10.1080/01615440.2018.1444523

77. Pooley, S.
Descent with modification: Critical use of historical evidence for conservation
(2018) Conservation Letters, 11 (4), art. no. e12437, . Cited 5 times.

DOI: 10.1111/conl.12437

78. Bugnot, A.B., Lyons, M.B., Scanes, P., Clark, G.F., Fyfe, S.K., Lewis, A., Johnston, E.L.
A novel framework for the use of remote sensing for monitoring catchments at continental scales
(2018) Journal of Environmental Management, 217, pp. 939-950. Cited 5 times.

DOI: 10.1016/j.jenvman.2018.03.058

79. Wellman, H.P.
Applied zooarchaeology and Oregon Coast sea otters (Enhydra lutris)
(2018) Marine Mammal Science, 34 (3), pp. 806-822.

DOI: 10.1111/mms.12484

80. Vild, O., Šipoš, J., Szabó, P., Macek, M., Chudomelová, M., Kopecký, M., Suchánková, S., Houška, J., Kotačka, M., Hédl, R.
Legacy of historical litter raking in temperate forest plant communities
(2018) Journal of Vegetation Science, 29 (4), pp. 596-606. Cited 4 times.

DOI: 10.1111/jvs.12642

81. Watling, J., Mayle, F.E., Schaan, D.
Historical ecology, human niche construction and landscape in pre-Columbian Amazonia: A case study of the geoglyph builders of Acre, Brazil
(2018) Journal of Anthropological Archaeology, 50, pp. 128-139. Cited 6 times.

DOI: 10.1016/j.jaa.2018.05.001

82. Wolsak, S., Wingate, D., Cronk, Q.
Environmental change in the terrestrial vegetation of Bermuda: Revisiting Harshberger (1905)
(2018) Brittonia, 70 (2), pp. 257-275. Cited 1 time.

DOI: 10.1007/s12228-017-9509-x

83. Thurstan, R.H., Brittain, Z., Jones, D.S., Cameron, E., Dearnaley, J., Bellgrove, A.
Aboriginal uses of seaweeds in temperate Australia: an archival assessment

(2018) Journal of Applied Phycology, 30 (3), pp. 1821-1832. Cited 7 times.

DOI: 10.1007/s10811-017-1384-z

84. Chernova, O.V., Ryzhova, I.M., Podvezennaya, M.A.
The Effect of Historical and Regional Features of Land Use on the Size and Structure of Carbon Pools in the Southern Taiga and Forest-Steppe Zones of European Russia
(2018) Eurasian Soil Science, 51 (6), pp. 709-719. Cited 2 times.

DOI: 10.1134/S106422931804004X

85. Tulowiecki, S.J.
Information retrieval in physical geography: A method to recover geographical information from digitized historical documents
(2018) Progress in Physical Geography, 42 (3), pp. 369-390. Cited 3 times.

DOI: 10.1177/0309133318770972

86. Piovesan, G., Mercuri, A.M., Mensing, S.A.
The potential of paleoecology for functional forest restoration planning: lessons from Late Holocene Italian pollen records
(2018) Plant Biosystems, 152 (3), pp. 508-514. Cited 8 times.

DOI: 10.1080/11263504.2018.1435582

87. Schurman, J.S., Trotsiuk, V., Bače, R., Čada, V., Fraver, S., Janda, P., Kulakowski, D., Labusova, J., Mikoláš, M., Nagel, T.A., Seidl, R., Synek, M., Svobodová, K., Chaskovskyy, O., Teodosiu, M., Svoboda, M.
Large-scale disturbance legacies and the climate sensitivity of primary Picea abies forests
(2018) Global Change Biology, 24 (5), pp. 2169-2181. Cited 29 times.

DOI: 10.1111/gcb.14041

88. Quintus, S.
Historicizing Food Production in Polynesia: A Case Study of 2700 years of Land Use on Ofu Island, American Samoa
(2018) Journal of Field Archaeology, 43 (3), pp. 222-235. Cited 4 times.

DOI: 10.1080/00934690.2018.1437316

89. Fitzpatrick, S.M., Erlandson, J.M.
Island Archaeology, Model Systems, the Anthropocene, and How the Past Informs the Future
(2018) Journal of Island and Coastal Archaeology, 13 (2), pp. 279-295. Cited 9 times.

DOI: 10.1080/15564894.2018.1447051

90. Ross, P.M., Beentjes, M.P., Cope, J., de Lange, W.P., McFadgen, B.G., Redfearn, P., Searle, B., Skerrett, M., Smith, H., Smith, S., Te Tuhi, J., Tamihana, J., Williams, J.R.
The biology, ecology and history of toheroa (Paphies ventricosa): a review of scientific, local and customary knowledge
(2018) New Zealand Journal of Marine and Freshwater Research, 52 (2), pp. 196-231. Cited 1 time.

DOI: 10.1080/00288330.2017.1383279

91. Newsome, S.D., Chivers, S.J., Berman Kowalewski, M.
The influence of lipid-extraction and long-term DMSO preservation on carbon (δ13C) and nitrogen (δ15N) isotope values in cetacean skin
(2018) Marine Mammal Science, 34 (2), pp. 277-293. Cited 4 times.

DOI: 10.1111/mms.12454

92. Roman, L.A., Pearsall, H., Eisenman, T.S., Conway, T.M., Fahey, R.T., Landry, S., Vogt, J., van Doorn, N.S., Grove, J.M., Locke, D.H., Bardekjian, A.C., Battles, J.J., Cadenasso, M.L., van den Bosch, C.C.K., Avolio, M., Berland, A., Jenerette, G.D., Mincey, S.K., Pataki, D.E., Staudhammer, C.
Human and biophysical legacies shape contemporary urban forests: A literature synthesis
(2018) Urban Forestry and Urban Greening, 31, pp. 157-168. Cited 27 times.

DOI: 10.1016/j.ufug.2018.03.004

93. Hanberry, B.B., Coursey, K., Kush, J.S.
Structure and Composition of Historical Longleaf Pine Ecosystems in Mississippi, USA
(2018) Human Ecology, 46 (2), pp. 241-248. Cited 11 times.

DOI: 10.1007/s10745-018-9982-1

94. Imlay, T.L., Mills Flemming, J., Saldanha, S., Wheelwright, N.T., Leonard, M.L.
Breeding phenology and performance for four swallows over 57 years: relationships with temperature and precipitation
(2018) Ecosphere, 9 (4), art. no. e02166, . Cited 9 times.

DOI: 10.1002/ecs2.2166

95. Ziter, C., Turner, M.G.
Current and historical land use influence soil-based ecosystem services in an urban landscape
(2018) Ecological Applications, 28 (3), pp. 643-654. Cited 13 times.

DOI: 10.1002/eap.1689

96. Hofman, C.A., Rick, T.C.
Ancient Biological Invasions and Island Ecosystems: Tracking Translocations of Wild Plants and Animals
(2018) Journal of Archaeological Research, 26 (1), pp. 65-115. Cited 12 times.

DOI: 10.1007/s10814-017-9105-3

97. Whitlock, C., Colombaroli, D., Conedera, M., Tinner, W.
Land-use history as a guide for forest conservation and management
(2018) Conservation Biology, 32 (1), pp. 84-97. Cited 23 times.

DOI: 10.1111/cobi.12960

98. Manton, M., Angelstam, P.

Defining benchmarks for restoration of green infrastructure: A case study combining the historical range of variability of habitat and species' requirements
(2018) Sustainability (Switzerland), 10 (2), art. no. 326, . Cited 7 times.

DOI: 10.3390/su10020326

99. Pilø, L., Finstad, E., Ramsey, C.B., Martinsen, J.R.P., Nesje, A., Solli, B., Wangen, V., Callanan, M., Barrett, J.H.
The chronology of reindeer hunting on Norway's highest ice patches
(2018) Royal Society Open Science, 5 (1), art. no. 171738, . Cited 6 times.

DOI: 10.1098/rsos.171738

100. Astudillo, F.J.
Soil phytoliths as indicators of initial human impact on San Cristóbal Island, Galápagos
(2018) Palaeogeography, Palaeoclimatology, Palaeoecology, 490, pp. 522-532. Cited 1 time.

DOI: 10.1016/j.palaeo.2017.11.038

101. Da Silva Sales, G.P., Guedes-Bruni, R.R.
A green puzzle "mountig the pieces" of the reforestation undertaken in Tijuca Forest [Um Quebra-Cabeça Verde: "montando as Peças" do Reflorestamento Empreendido na Floresta da Tijuca]
(2018) Fronteiras, 7 (3), pp. 58-77.

DOI: 10.21664/2238-8869.2018v7i3

102. Ashmore, W.
10 Why the Archaeology of Political Ecology Matters
(2018) Archeological Papers of the American Anthropological Association, 29 (1), pp. 175-184. Cited 1 time.

DOI: 10.1111/apaa.12105

103. van Erp, M., de Does, J., Depuydt, K., Lenders, R., van Goethem, T.
Slicing and dicing a newspaper corpus for historical ecology research
(2018) Lecture Notes in Computer Science (including subseries Lecture Notes in Artificial Intelligence and Lecture Notes in Bioinformatics), 11313, pp. 470-484. Cited 2 times.

DOI: 10.1007/978-3-030-03667-6_30

104. Szymura, T.H., Murak, S., Szymura, M., Raduła, M.W.
Changes in forest cover in Sudety Mountains during the last 250 years: Patterns, drivers, and landscape-scale implications for nature conservation
(2018) Acta Societatis Botanicorum Poloniae, 87 (1), art. no. 3576, .
DOI: 10.5586/asbp.3576

105. Siqueira, M.V.B.M., De Camargo E Timo, T.P., De Paula-Souza, J., Balée, W.
Traditional knowledge of trees and cultivated plants in a coastal municipallity in Sao Paulo State, Brazil: A cognitive experience
(2018) Fronteiras, 7 (1), pp. 249-264.

DOI: 10.21664/2238-8869.2018v7i1.249-264

106. Hody, J.W., Kays, R.
Mapping the expansion of coyotes (Canis latrans) across North and central America
(2018) ZooKeys, 2018 (759), pp. 81-97. Cited 34 times.

DOI: 10.3897/ZOOKEYS.759.15149

107. Eddy, T.D., Cheung, W.W.L., Bruno, J.F.
Historical baselines of coral cover on tropical reefs as estimated by expert opinion
(2018) PeerJ, 2018 (1), art. no. e4308, . Cited 4 times.

DOI: 10.7717/peerj.4308

108. Pawlik, J.R., Loh, T.-L., McMurray, S.E.
A review of bottom-up vs. top-down control of sponges on Caribbean fore-reefs: What's old, what's new, and future directions
(2018) PeerJ, 2018 (1), art. no. e4343, . Cited 16 times.

DOI: 10.7717/peerj.4343

109. Vampilova, L.B., Kostovska, S.K., Krylova, A.N., Lobkovsky, V.A., Lobkovskaya, L.G.
The analysis of the existing techniques of the ecological and geographical assessment of the regions of russia
(2018) International Multidisciplinary Scientific GeoConference Surveying Geology and Mining Ecology Management, SGEM, 18 (5.2), pp. 913-920.

DOI: 10.5593/sgem2018/5.2/S20.118

2017

110. Boucher Y, Auger I, Noel J, Grondin P, Arseneault D
Fire is a stronger driver of forest composition than logging in the boreal forest of eastern Canada.
(2017) Journal of Vegetation Science 28, Issue 1, Pages 57 - 68.

DOI: 10.1111/jvs.12466

111. Sorte CJB, Davidson VE, Franklin MC, Benes KM, Doellman MM, Etter RJ, Hannigan RE, Lubchenco J, Menge BA
Long-term declines in an intertidal foundation species parallel shifts in community composition.
(2017) Global Change Biology 23, Issue 1, Pages 341 – 352

DOI: 10.1111/gcb.13425

112. Fritts AK, Fritts MW, Haag WR, DeBoer JA, Casper AF
Freshwater mussel shells (Unionidae) chronicle changes in a North American river over the past 1000 years.
(2017) Science of the total environment 575, Pages 199 - 206.

DOI: 10.1016/j.scitotenv.2016.09.225

113. Plue J, Vandepitte K, Honnay O, Cousins SAO
Does the seed bank contribute to the build-up of a genetic extinction debt in the grassland perennial Campanula rotundifolia?

(2017) Annals of Botany 120, Issue 3, Pages 373 - 385.

DOI: 10.1093/aob/mcx057

114. Oreska MPJ, Truitt B, Orth RJ, Luckenbach MW
The bay scallop (Argopecten irradians) industry collapse in Virginia and its implications for the successful management of scallop-seagrass habitats.
(2017) Marine Policy 75, Pages 116 - 124.

DOI: 10.1016/j.marpol.2016.10.021

115. Poderoso, R.A., Peroni, N., Hanazaki, N.
Gender Influences in the Perception and Use of the Landscape in a Rural Community of German Immigrant Descendants in Brazil
(2017) Journal of Ethnobiology, 37 (4), pp. 779-797. Cited 2 times.

DOI: 10.2993/0278-0771-37.4.779

116. Crumley, C.L.
Historical ecology and the study of landscape
(2017) Landscape Research, 42, pp. S65-S73. Cited 3 times.

DOI: 10.1080/01426397.2017.1399994

117. Adams, A.J., Pessier, A.P., Briggs, C.J.
Rapid extirpation of a North American frog coincides with an increase in fungal pathogen prevalence: Historical analysis and implications for reintroduction
(2017) Ecology and Evolution, 7 (23), pp. 10216-10232. Cited 9 times.

DOI: 10.1002/ece3.3468

118. Buckley, S.M., Thurstan, R.H., Tobin, A., Pandolfi, J.M.
Historical spatial reconstruction of a spawning-aggregation fishery
(2017) Conservation Biology, 31 (6), pp. 1322-1332. Cited 5 times.

DOI: 10.1111/cobi.12940

119. Moreira, P.A., Aguirre-Dugua, X., Mariac, C., Zekraoui, L., Couderc, M., Rodrigues, D.P., Casas, A., Clement, C.R., Vigouroux, Y.
Diversity of Treegourd (Crescentia cujete) suggests introduction and prehistoric dispersal routes into Amazonia
(2017) Frontiers in Ecology and Evolution, 5 (NOV), art. no. 150, . Cited 2 times.

DOI: 10.3389/fevo.2017.00150

120. Ziter, C., Graves, R.A., Turner, M.G.
How do land-use legacies affect ecosystem services in United States cultural landscapes?
(2017) Landscape Ecology, 32 (11), pp. 2205-2218. Cited 14 times.

DOI: 10.1007/s10980-017-0545-4

121. Auffret, A.G., Kimberley, A., Plue, J., Skånes, H., Jakobsson, S., Waldén, E., Wennbom, M., Wood, H., Bullock, J.M., Cousins, S.A.O., Gartz, M., Hooftman, D.A.P., Tränk, L.

HistMapR: Rapid digitization of historical land-use maps in R
(2017) Methods in Ecology and Evolution, 8 (11), pp. 1453-1457. Cited 9 times.

DOI: 10.1111/2041-210X.12788

122. Danneyrolles, V., Dupuis, S., Arseneault, D., Terrail, R., Leroyer, M., de Römer, A., Fortin, G., Boucher, Y., Ruel, J.-C.
Eastern white cedar long-term dynamics in eastern Canada: Implications for restoration in the context of ecosystem-based management
(2017) Forest Ecology and Management, 400, pp. 502-510. Cited 10 times.

DOI: 10.1016/j.foreco.2017.06.024

123. Thurstan, R.H., Pandolfi, J.M., zu Ermgassen, P.S.E.
Animal forests through time: Historical data to understand present changes in marine ecosystems
(2017) Marine Animal Forests: The Ecology of Benthic Biodiversity Hotspots, pp. 947-963. Cited 3 times.

DOI: 10.1007/978-3-319-21012-4_31

124. Compton, T.J., Holthuijsen, S., Mulder, M., van Arkel, M., Schaars, L.K., Koolhaas, A., Dekinga, A., ten Horn, J., Luttikhuizen, P.C., van der Meer, J., Piersma, T., van der Veer, H.W.
Shifting baselines in the Ems Dollard estuary: A comparison across three decades reveals changing benthic communities
(2017) Journal of Sea Research, 127, pp. 119-132. Cited 2 times.

DOI: 10.1016/j.seares.2017.06.014

125. Lepofsky, D., Armstrong, C.G., Greening, S., Jackley, J., Carpenter, J., Guernsey, B., Mathews, D., Turner, N.J.
Historical Ecology of Cultural Keystone Places of the Northwest Coast
(2017) American Anthropologist, 119 (3), pp. 448-463. Cited 12 times.

DOI: 10.1111/aman.12893

126. Hartman, S., Ogilvie, A.E.J., Ingimundarson, J.H., Dugmore, A.J., Hambrecht, G., McGovern, T.H.
Medieval Iceland, Greenland, and the New Human Condition: A case study in integrated environmental humanities
(2017) Global and Planetary Change, 156, pp. 123-139. Cited 12 times.

DOI: 10.1016/j.gloplacha.2017.04.007

127. Hückstädt, L.A., McCarthy, M.D., Koch, P.L., Costa, D.P.
What difference does a century make? Shifts in the ecosystem structure of the Ross Sea, Antarctica, as evidenced from a sentinel species, the weddell seal
(2017) Proceedings of the Royal Society B: Biological Sciences, 284 (1861), art. no. 20170927, . Cited 5 times.

DOI: 10.1098/rspb.2017.0927

128. Stagno, A.M.
Archaeology and history of an uncertain space: Temporary cultivation between collective practices and private appropriation (Basque

Country,15th-20th c.) [Archeologia e storia di uno spazio precario: Le colture temporanee tra pratiche collettive e appropriazione privata (Paesi Baschi, XV-XX secolo)]
(2017) Quaderni Storici, 52 (2), pp. 499-533. Cited 1 time.

DOI: 10.1408/89384

129. Morrison, S.A., Sillett, T.S., Funk, W.C., Ghalambor, C.K., Rick, T.C.
Equipping the 22nd-Century Historical Ecologist
(2017) Trends in Ecology and Evolution, 32 (8), pp. 578-588. Cited 18 times.

DOI: 10.1016/j.tree.2017.05.006

130. Koungoulos, L.
Canis dingo and the Australian smaller-fauna trend: A new explanatory model integrating ecological data
(2017) Journal of Archaeological Science: Reports, 14, pp. 38-45. Cited 1 time.

DOI: 10.1016/j.jasrep.2017.05.035

131. Mattocks, S., Hall, C.J., Jordaan, A.
Damming, lost connectivity, and the historical role of anadromous fish in freshwater ecosystem dynamics
(2017) BioScience, 67 (8), pp. 713-728. Cited 9 times.

DOI: 10.1093/biosci/bix069

132. da Silva, T.C., Medeiros, M.F.T., Peroni, N., Paulino Albuquerque, U.
Folk classification as evidence of transformed landscapes and adaptative strategies: a case study in the semiarid region of northeastern Brazil
(2017) Landscape Research, 42 (5), pp. 521-532. Cited 5 times.

DOI: 10.1080/01426397.2016.1258047

133. Levin, M.J., Shelton, M., Ayres, W.S.
Swiddening Practices in the Micronesian Past: Macrocharcoal Quantification From a Gardening Site in Pohnpei
(2017) Journal of Island and Coastal Archaeology, 12 (3), pp. 451-457. Cited 3 times.

DOI: 10.1080/15564894.2016.1271066

134. Tomašových, A., Kidwell, S.M.
Nineteenth-century collapse of a benthic marine ecosystem on the open continental shelf
(2017) Proceedings of the Royal Society B: Biological Sciences, 284 (1856), art. no. 20170328, . Cited 22 times.

DOI: 10.1098/rspb.2017.0328

135. Coughlan, M.R., Nelson, D.R., Lonneman, M., Block, A.E.
Historical land use dynamics in the highly degraded landscape of the Calhoun critical zone Observatory
(2017) Land, 6 (2), art. no. 32, . Cited 11 times.

DOI: 10.3390/land6020032

136. Copes-Gerbitz, K., Arabas, K., Larson, E., Gildehaus, S.
A Multi-Proxy Environmental Narrative of Oregon White Oak (Quercus garryana) Habitat in the Willamette Valley, Oregon
(2017) Northwest Science, 91 (2), pp. 160-185. Cited 3 times.

DOI: 10.3955/046.091.0207

137. Pasanen-Mortensen, M., Elmhagen, B., Lindén, H., Bergström, R., Wallgren, M., van der Velde, Y., Cousins, S.A.O.
The changing contribution of top-down and bottom-up limitation of mesopredators during 220 years of land use and climate change
(2017) Journal of Animal Ecology, 86 (3), pp. 566-576. Cited 8 times.

DOI: 10.1111/1365-2656.12633

138. Al-Abdulrazzak, D., Pauly, D.
Reconstructing historical baselines for the Persian/Arabian Gulf Dugong, Dugong dugon (Mammalia: Sirena)
(2017) Zoology in the Middle East, 63 (2), pp. 95-102. Cited 4 times.

DOI: 10.1080/09397140.2017.1315853

139. Quintus, S., Clark, J.T.
Historical ecology of Ofu Island revisited: new data for an island-wide model
(2017) Archaeology in Oceania, 52 (1), pp. 62-69. Cited 1 time.

DOI: 10.1002/arco.5116

140. Paal, T., Kütt, L., Lõhmus, K., Liira, J.
Both spatiotemporal connectivity and habitat quality limit the immigration of forest plants into wooded corridors
(2017) Plant Ecology, 218 (4), pp. 417-431. Cited 13 times.

DOI: 10.1007/s11258-017-0700-7

141. Bernards, S.J., Morris, L.R.
Influence of topography on long-term successional trajectories in canyon grasslands
(2017) Applied Vegetation Science, 20 (2), pp. 236-246. Cited 8 times.

DOI: 10.1111/avsc.12272

142. Goring, S.J., Williams, J.W.
Effect of historical land-use and climate change on tree-climate relationships in the upper Midwestern United States
(2017) Ecology Letters, 20 (4), pp. 461-470. Cited 9 times.

DOI: 10.1111/ele.12747

143. Ramenzoni, V.C.
Reconstructing the history and the effects of mechanization in a small-scale fishery of Flores, Eastern Indonesia (1917-2014)
(2017) Frontiers in Marine Science, 4 (MAR), art. no. 65, .

DOI: 10.3389/fmars.2017.00065

144. Fortibuoni, T., Giovanardi, O., Pranovi, F., Raicevich, S., Solidoro, C., Libralato, S.
Analysis of long-term changes in a mediterranean marine ecosystem based on fishery landings
(2017) Frontiers in Marine Science, 4 (FEB), art. no. 33, . Cited 12 times.

DOI: 10.3389/FMARS.2017.00033

145. Sibert, E. C., Cramer, K. L., Hastings, P. A., & Norris, R. D. (2017). Methods for isolation and quantification of microfossil fish teeth and elasmobranch dermal denticles (ichthyoliths) from marine sediments. Palaeontologia Electronica, 20(1), 1-14.

DOI: 10.26879/677

146. Vales, D.G., Cardona, L., Zangrando, A.F., Borella, F., Saporiti, F., Goodall, R.N.P., de Oliveira, L.R., Crespo, E.A.
Holocene changes in the trophic ecology of an apex marine predator in the South Atlantic Ocean
(2017) Oecologia, 183 (2), pp. 555-570. Cited 7 times.

DOI: 10.1007/s00442-016-3781-4

147. Culbert, P.D., Dorresteijn, I., Loos, J., Clayton, M.K., Fischer, J., Kuemmerle, T.
Legacy effects of past land use on current biodiversity in a low-intensity farming landscape in Transylvania (Romania)
(2017) Landscape Ecology, 32 (2), pp. 429-444. Cited 6 times.

DOI: 10.1007/s10980-016-0441-3

148. Auffret, A.G., Aggemyr, E., Plue, J., Cousins, S.A.O.
Spatial scale and specialization affect how biogeography and functional traits predict long-term patterns of community turnover
(2017) Functional Ecology, 31 (2), pp. 436-443. Cited 11 times.

DOI: 10.1111/1365-2435.12716

149. Szabó, P., Kuneš, P., Svobodová-Svitavská, H., Švarcová, M.G., Křížová, L., Suchánková, S., Müllerová, J., Hédl, R.
Using historical ecology to reassess the conservation status of coniferous forests in Central Europe
(2017) Conservation Biology, 31 (1), pp. 150-160. Cited 17 times.

DOI: 10.1111/cobi.12763

150. Fortuny, X., Chauchard, S., Carcaillet, C.
Confounding legacies of land uses and land-form pattern on the regional vegetation structure and diversity of Mediterranean montane forests
(2017) Forest Ecology and Management, 384, pp. 268-278. Cited 4 times.

DOI: 10.1016/j.foreco.2016.11.002

151. Clavero, M., Ninyerola, M., Hermoso, V., Filipe, A.F., Pla, M., Villero, D., Brotons, L., Delibes, M.
Historical citizen science to understand and predict climate-driven trout decline

(2017) Proceedings of the Royal Society B: Biological Sciences, 284 (1846), art. no. 20161979, . Cited 9 times.

DOI: 10.1098/rspb.2016.1979

152. Eriksson, O., Arnell, M.
Niche construction, entanglement and landscape domestication in Scandinavian infield systems
(2017) Landscape Research, 42 (1), pp. 78-88. Cited 5 times.

DOI: 10.1080/01426397.2016.1255316

153. Van Erp, M., Van Goethem, T., Depuydt, K., De Does, J.
Towards semantic enrichment of newspapers: A historical ecology use case
(2017) CEUR Workshop Proceedings, 2014, pp. 39-44.

PENDIENTE 154. Braje, T.J., Campbell, B., Haas, H.
Assessing California mussel (Mytilus Californianus) size changes through deep time: A methodological case study from San Miguel Island, California
(2017) Zooarchaeology in Practice: Case Studies in Methodology and Interpretation in Archaeofaunal Analysis, pp. 291-307.

DOI: 10.1007/978-3-319-64763-0_15

155. Bao, K., Drew, J.
Traditional ecological knowledge, shifting baselines, and conservation of Fijian molluscs
(2017) Pacific Conservation Biology, 23 (1), pp. 81-87. Cited 4 times.

DOI: 10.1071/PC16016

156. White, S.M., Justice, C., Kelsey, D.A., McCullough, D.A., Smith, T.
Legacies of stream channel modification revealed using General Land Office surveys, with implications for water temperature and aquatic life
(2017) Elementa, 5, art. no. 3, . Cited 8 times.

DOI: 10.1525/elementa.192

157. Bürgi M, Östlund L, Mladenoff
Legacy Effects of Human Land Use: Ecosystems as Time-Lagged Systems
(2017) Ecosystems 20:94–103

DOI: 10.1007/s10021-016-0051-6

2016

158. McKey, D.B., Durécu, M., Pouilly, M., Béarez, P., Ovando, A., Kalebe, M., Huchzermeyer, C.F.
Present-day African analogue of a pre-European Amazonian floodplain fishery shows convergence in cultural niche construction
(2016) Proceedings of the National Academy of Sciences of the United States of America, 113 (52), pp. 14938-14943. Cited 10 times.

DOI: 10.1073/pnas.1613169114

159. Samojlik, T., Fedotova, A., Kuijper, D.P.J.

Transition from traditional to modern forest management shaped the spatial extent of cattle pasturing in Białowieża Primeval Forest in the nineteenth and twentieth centuries
(2016) Ambio, 45 (8), pp. 904-918. Cited 13 times.

DOI: 10.1007/s13280-016-0795-4

160. Schmidt, M., Mölder, A., Schönfelder, E., Engel, F., Fortmann-Valtink, W.
Charcoal kiln sites, associated landscape attributes and historic forest conditions: DTM-based investigations in Hesse (Germany)
(2016) Forest Ecosystems, 3 (1), art. no. 8, . Cited 15 times.

DOI: 10.1186/s40663-016-0067-6

161. Drew, J., López, E.H., Gill, L., McKeon, M., Miller, N., Steinberg, M., Shen, C., McClenachan, L.
Collateral damage to marine and terrestrial ecosystems from Yankee whaling in the 19th century
(2016) Ecology and Evolution, 6 (22), pp. 8181-8192. Cited 3 times.

DOI: 10.1002/ece3.2542

162. Clavero, M., Nores, C., Kubersky-Piredda, S., Centeno-Cuadros, A.
Interdisciplinarity to reconstruct historical introductions: solving the status of cryptogenic crayfish
(2016) Biological Reviews, 91 (4), pp. 1036-1049. Cited 17 times.

DOI: 10.1111/brv.12205

163. Fernandes, M., Adams, J.
Quantifying the loss of and changes in estuary habitats in the uMkhomazi and Mvoti estuaries, South Africa
(2016) South African Journal of Botany, 107, pp. 179-187. Cited 8 times.

DOI: 10.1016/j.sajb.2016.04.009

164. Boles, O.J.C., Lane, P.J.
The Green, Green Grass of Home: an archaeo-ecological approach to pastoralist settlement in central Kenya
(2016) Azania, 51 (4), pp. 507-530. Cited 12 times.

DOI: 10.1080/0067270X.2016.1249587

165. Reeder-Myers, L., Rick, T., Lowery, D., Wah, J., Henkes, G.
Human Ecology and Coastal Foraging at Fishing Bay, Maryland, USA
(2016) Journal of Ethnobiology, 36 (3), pp. 595-616. Cited 4 times.

DOI: 10.2993/0278-0771-36.3.595

166. Frans, V.F., Augé, A.A.
Use of local ecological knowledge to investigate endangered baleen whale recovery in the Falkland Islands
(2016) Biological Conservation, 202, pp. 127-137. Cited 15 times.

DOI: 10.1016/j.biocon.2016.08.017

167. Isendahl, C.

Historical ecology coming of age
(2016) Reviews in Anthropology, 45 (3-4), pp. 127-147. Cited 4 times.

DOI: 10.1080/00938157.2016.1210962

168. Morris, L.R., Monaco, T.A., Blank, R., Sheley, R.L.
Cultivation legacies in soils after rehabilitation seeding in the Great Basin, USA
(2016) Arid Land Research and Management, 30 (4), pp. 362-374. Cited 4 times.

DOI: 10.1080/15324982.2015.1129651

169. Klein, E.S., Glaser, S.M., Jordaan, A., Kaufman, L., Rosenberg, A.A.
A complex past: Historical and contemporary fisheries demonstrate nonlinear dynamics and a loss of determinism
(2016) Marine Ecology Progress Series, 557, pp. 237-246. Cited 5 times.

DOI: 10.3354/meps11886

170. Lambrides, A.B.J., Weisler, M.I.
Pacific Islands Ichthyoarchaeology: Implications for the Development of Prehistoric Fishing Studies and Global Sustainability
(2016) Journal of Archaeological Research, 24 (3), pp. 275-324. Cited 20 times.

DOI: 10.1007/s10814-016-9090-y

171. Kujawa, E.R., Goring, S., Dawson, A., Calcote, R., Grimm, E.C., Hotchkiss, S.C., Jackson, S.T., Lynch, E.A., McLachlan, J., St-Jacques, J.-M., Umbanhowar Jr., C., Williams, J.W.
The effects of anthropogenic land cover change on pollen-vegetation relationships in the American Midwest
(2016) Anthropocene, 15, pp. 60-71. Cited 10 times.

DOI: 10.1016/j.ancene.2016.09.005

172. Sáenz-Arroyo, A., Revollo-Fernández, D.
Local ecological knowledge concurs with fishing statistics: An example from the abalone fishery in Baja California, Mexico
(2016) Marine Policy, 71, pp. 217-221. Cited 15 times.

DOI: 10.1016/j.marpol.2016.06.006

173. Izzo, C., Doubleday, Z.A., Grammer, G.L., Gilmore, K.L., Alleway, H.K., Barnes, T.C., Disspain, M.C.F., Giraldo, A.J., Mazloumi, N., Gillanders, B.M.
Fish as proxies of ecological and environmental change
(2016) Reviews in Fish Biology and Fisheries, 26 (3), pp. 265-286. Cited 24 times.

DOI: 10.1007/s11160-016-9424-3

174. Ferretti, F., Morey Verd, G., Seret, B., Sulić Šprem, J., Micheli, F.
Falling through the cracks: the fading history of a large iconic predator
(2016) Fish and Fisheries, 17 (3), pp. 875-889. Cited 14 times.

DOI: 10.1111/faf.12108

175. Santana-Cordero, A.M., Monteiro-Quintana, M.L., Hernández-Calvento, L.
Reconstruction of the land uses that led to the termination of an arid coastal dune system: The case of the Guanarteme dune system (Canary Islands, Spain), 1834-2012
(2016) Land Use Policy, 55, pp. 73-85. Cited 28 times.

DOI: 10.1016/j.landusepol.2016.02.021

176. Schmidt, M., Meyer, P., Mölder, A., Hondong, H.
New or re-colonization? The late 19th century range expansion of the black woodpecker (Dryocopus martius) in northwest Germany and its underlying causes [Neu-Oder Wiederausbreitung? Die Arealausweitung des Schwarzspechts in Nordwestdeutschland am Ende des 19. Jahrhunderts und ihre Ursachen]
(2016) Forstarchiv, 87 (5), pp. 135-151. Cited 5 times.

DOI: 10.4432/0300-4112-87-135

177. McKechnie, I., Moss, M.L.
Meta-analysis in zooarchaeology expands perspectives on Indigenous fisheries of the Northwest Coast of North America
(2016) Journal of Archaeological Science: Reports, 8, pp. 470-485. Cited 25 times.

DOI: 10.1016/j.jasrep.2016.04.006

178. Sguotti, C., Lynam, C.P., García-Carreras, B., Ellis, J.R., Engelhard, G.H.
Distribution of skates and sharks in the North Sea: 112 years of change
(2016) Global change biology, 22 (8), pp. 2729-2743. Cited 25 times.

DOI: 10.1111/gcb.13316

179. Mölder, A.
Small forest parcels, management diversity and valuable coppice habitats: An 18th century political compromise in the Osnabrück region (NW Germany) and its long-lasting legacy
(2016) IForest, 9 (4), pp. 518-528. Cited 6 times.

DOI: 10.3832/ifor1834-009

180. Fuchs, R., Schulp, C.J., Hengeveld, G.M., Verburg, P.H., Clevers, J.G., Schelhaas, M.-J., Herold, M.
Assessing the influence of historic net and gross land changes on the carbon fluxes of Europe
(2016) Global change biology, 22 (7), pp. 2526-2539. Cited 27 times.

DOI: 10.1111/gcb.13191

181. Fortibuoni, T., Borme, D., Franceschini, G., Giovanardi, O., Raicevich, S.
Common, rare or extirpated? Shifting baselines for common angelshark, Squatina squatina (Elasmobranchii: Squatinidae), in the Northern Adriatic Sea (Mediterranean Sea)
(2016) Hydrobiologia, 772 (1), pp. 247-259. Cited 21 times.

DOI: 10.1007/s10750-016-2671-4

182. Danneyrolles, V., Arseneault, D., Bergeron, Y.
Long-term compositional changes following partial disturbance revealed by the resurvey of logging concession limits in the northern temperate forest of eastern Canada
(2016) Canadian Journal of Forest Research, 46 (7), pp. 943-949. Cited 9 times.

DOI: 10.1139/cjfr-2016-0047

183. López-Angarita, J., Roberts, C.M., Tilley, A., Hawkins, J.P., Cooke, R.G.
Mangroves and people: Lessons from a history of use and abuse in four Latin American countries
(2016) Forest Ecology and Management, 368, pp. 151-162. Cited 27 times.

DOI: 10.1016/j.foreco.2016.03.020

184. Jones, P., Cathcart, A., Speirs, D.C.
Early evidence of the impact of preindustrial fishing on fish stocks from the mid-west and southeast coastal fisheries of Scotland in the 19th century
(2016) ICES Journal of Marine Science, 73 (5), pp. 1404-1414. Cited 3 times.

DOI: 10.1093/icesjms/fsv189

185. MacDiarmid, A., Mackenzie, B., Ojaveer, H.
Multidisciplinary perspectives on the history of human interactions with life in the ocean
(2016) ICES Journal of Marine Science, 73 (5), pp. 1382-1385. Cited 2 times.

DOI: 10.1093/icesjms/fsw059

186. Engelhard, G.H., Thurstan, R.H., MacKenzie, B.R., Alleway, H.K., Bannister, R.C.A., Cardinale, M., Clarke, M.W., Currie, J.C., Fortibuoni, T., Holm, P., Holt, S.J., Mazzoldi, C., Pinnegar, J.K., Raicevich, S., Volckaert, F.A.M., Klein, E.S., Lescrauwaet, A.-K.
ICES meets marine historical ecology: Placing the history of fish and fisheries in current policy context
(2016) ICES Journal of Marine Science, 73 (5), pp. 1386-1403. Cited 35 times.

DOI: 10.1093/icesjms/fsv219

187. Coughlan, M.R.
Wildland Arson as Clandestine Resource Management: A Space-Time Permutation Analysis and Classification of Informal Fire Management Regimes in Georgia, USA
(2016) Environmental Management, 57 (5), pp. 1077-1087. Cited 6 times.

DOI: 10.1007/s00267-016-0669-3

188. Bellquist, L., Semmens, B.X.
23481412500;6507755514;
Temporal and spatial dynamics of 'trophy'-sized demersal fishes off the California (USA) coast, 1966 to 2013
(2016) Marine Ecology Progress Series, 547, pp. 1-18. Cited 9 times.

DOI: 10.3354/meps11667

189. Sluyter, A., Duvall, C.
6603563118;22933981100;
African Fire Cultures, Cattle Ranching, and Colonial Landscape Transformations in the Neotropics
(2016) Geographical Review, 106 (2), pp. 294-311. Cited 4 times.

DOI: 10.1111/j.1931-0846.2015.12138.x

190. Santana Cordero, A., Monteiro Quintana, M.L., Hernández Calvento, L., Pérez-Chacón Espino, E., García Romero, L.
Long-term Human Impacts on the Coast of La Graciosa, Canary Islands
(2016) Land Degradation and Development, 27 (3), pp. 479-489. Cited 19 times.

DOI: 10.1002/ldr.2369

191. Morris, L.R., Leger, E.A.
Secondary Succession in the Sagebrush Semidesert 66 Years after Fire in the Great Basin, USA
(2016) Natural Areas Journal, 36 (2), pp. 187-193. Cited 11 times.

DOI: 10.3375/043.036.0211

192. Perring, M.P., De Frenne, P., Baeten, L., Maes, S.L., Depauw, L., Blondeel, H., Carón, M.M., Verheyen, K.
Global environmental change effects on ecosystems: The importance of land-use legacies
(2016) Global Change Biology, 22 (4), pp. 1361-1371. Cited 68 times.

DOI: 10.1111/gcb.13146

193. Thurstan, R.H., Buckley, S.M., Ortiz, J.C., Pandolfi, J.M.
Setting the Record Straight: Assessing the Reliability of Retrospective Accounts of Change
(2016) Conservation Letters, 9 (2), pp. 98-105. Cited 23 times.

DOI: 10.1111/conl.12184

194. Trott, T.J.
Century-scale species incidence, rareness and turnover in a high-diversity Northwest Atlantic coastal embayment
(2016) Marine Biodiversity, 46 (1), pp. 33-49. Cited 5 times.

DOI: 10.1007/s12526-015-0313-0

195. Srinath, I., Millington, A.C.
Evaluating the potential of the original texas land survey for mapping historical land and vegetation cover
(2016) Land, 5 (1), art. no. 4, . Cited 4 times.

DOI: 10.3390/land5010004

196. Young, M.A.L., Foale, S., Bellwood, D.R.
The last marine wilderness: Spearfishing for trophy fishes in the Coral Sea

(2016) Environmental Conservation, 43 (1), pp. 90-95. Cited 1 time.

DOI: 10.1017/S0376892915000272

197. Beller, E.E., Downs, P.W., Grossinger, R.M., Orr, B.K., Salomon, M.N.
From past patterns to future potential: using historical ecology to inform river restoration on an intermittent California river
(2016) Landscape Ecology, 31 (3), pp. 581-600. Cited 6 times.

DOI: 10.1007/s10980-015-0264-7

198. Selim, S.A., Blanchard, J.L., Bedford, J., Webb, T.J.
Direct and indirect effects of climate and fishing on changes in coastal ecosystem services: a historical perspective from the North Sea
(2016) Regional Environmental Change, 16 (2), pp. 341-351. Cited 8 times.

DOI: 10.1007/s10113-014-0635-7

199. Coughlan, M.R., Gragson, T.L.
An event history analysis of parcel extensification and household abandonment in Pays Basque, French Pyrenees, 1830-1958 AD
(2016) Human Ecology, 44 (1), pp. 65-80. Cited 3 times.

DOI: 10.1007/s10745-016-9808-y

200. Matthes, J.H., Goring, S., Williams, J.W., Dietze, M.C.
Benchmarking historical CMIP5 plant functional types across the Upper Midwest and Northeastern United States
(2016) Journal of Geophysical Research: Biogeosciences, 121 (2), pp. 523-535. Cited 13 times.

DOI: 10.1002/2015JG003175

201. Molinari, C., Montanari, C.
Interdisciplinary approach for reconstructing an alder-based historical agricultural practice of the Eastern Ligurian Apennines (NW Italy)
(2016) Environmental Archaeology, 21 (1), pp. 31-44. Cited 7 times.

DOI: 10.1179/1749631414Y.0000000056

202. Hawkins, S.J., Mieszkowska, N., Firth, L.B., Bohn, K., Burrows, M.T., Maclean, M.A., Thompson, R.C., Chan, B.K.K., Little, C., Williams, G.A.
Looking backwards to look forwards: The role of natural history in temperate reef ecology
(2016) Marine and Freshwater Research, 67 (1), pp. 1-13. Cited 8 times.

DOI: 10.1071/MF14413

203. Kolář, J., Tkáč, P., Macek, M., Szabó, P.
Archaeology and historical ecology: The archaeological database of the LONGWOOD ERC project
(2016) Archaologisches Korrespondenzblatt, 46 (4), pp. 539-554. Cited 2 times.

204. Kurek, P., Steppa, R., Grzywaczewski, G., Tryjanowski, P.
The silence of the lambs? Plant diversity in abandoned sheep pens
(2016) Plant, Soil and Environment, 62 (1), pp. 1-8. Cited 2 times.

DOI: 10.17221/327/2015-PSE

205. Fagúndez, J., Izco, J.
Spatial analysis of heath toponymy in relation to present-day heathland distribution
(2016) International Journal of Geographical Information Science, 30 (1), pp. 51-60. Cited 7 times.

DOI: 10.1080/13658816.2015.1017729

206. Wiens, J., Grenier, L., Grossinger, R., Healey, M.
The delta as changing landscapes
(2016) San Francisco Estuary and Watershed Science, 14 (2), art. no. 9, . Cited 6 times.

DOI: 10.15447/sfews.2016v14iss2art9

207. Thurstan, R.H., Campbell, A.B., Pandolfi, J.M.
Nineteenth century narratives reveal historic catch rates for Australian Snapper (Pagrus auratus)
(2016) Fish and Fisheries, 17 (1), pp. 210-225. Cited 17 times.

DOI: 10.1111/faf.12103

208. Romanin, L.M., Hopf, F., Haberle, S.G., Bowman, D.M.J.S.
Fire regime and vegetation change in the transition from Aboriginal to European land management in a Tasmanian eucalypt savanna
(2016) Australian Journal of Botany, 64 (5), pp. 427-440. Cited 12 times.

DOI: 10.1071/BT16032

209. McGregor, H.W., Colloff, M.J., Lunt, I.D.
Did early logging or changes in disturbance regimes promote high tree densities in river red gum forests'
(2016) Australian Journal of Botany, 64 (6), pp. 530-538. Cited 6 times.

DOI: 10.1071/BT16025

210. Jakobsson, S., Fukamachi, K., Cousins, S.A.O.
Connectivity and management enables fast recovery of plant diversity in new linear grassland elements
(2016) Journal of Vegetation Science, 27 (1), pp. 19-28. Cited 8 times.

DOI: 10.1111/jvs.12344

211. Cloern, J.E., Robinson, A., Richey, A., Grenier, L., Grossinger, R., Boyer, K.E., Burau, J., Canuel, E.A., DeGeorge, J.F., Drexler, J.Z., Enright, C., Howe, E.R., Kneib, R., Mueller-Solger, A., Naiman, R.J., Pinckney, J.L., Safran, S.M., Schoellhamer, D., Simenstad, C.
Primary production in the delta: Then and now
(2016) San Francisco Estuary and Watershed Science, 14 (3), art. no. 1, . Cited 5 times.

DOI: 10.15447/sfews.2016v14iss3art1

212. Laffan, S.W., Skidmore, A.K., Franklin, J.
6603019813;7006518879;35725372300;

Space, time, connectivity and conflict in biological landscapes: the fourth special issue on spatial ecology
(2016) International Journal of Geographical Information Science, 30 (1), pp. 1-4. Cited 3 times.

DOI: 10.1080/13658816.2015.1090001

2015

213. Walters, G., Schleicher, J., Hymas, O, Coad, L.
Evolving hunting practices in Gabon: lessons for community-based conservation interventions
(2015) Ecology and Society 20(4):31.

DOI: 10.5751/ES-08047-200431

214. Bush, M.B., McMichael, C.H., Piperno, D.R., Silman, M.R., Barlow, J., Peres, C.A., Power, M., Palace, M.W.
Anthropogenic influence on Amazonian forests in pre-history: An ecological Perspective
(2015) Journal of Biogeography 42, 2277–2288

DOI: 10.1111/jbi.12638

215. Fuchs R, Verburg P.H., Clevers J.G.P.W., Herold, M.
The potential of old maps and encyclopaedias for reconstructing historic European land cover/use change
(2015) Applied Geography 59, 43e55

DOI: 10.1016/j.apgeog.2015.02.013

216. Singh, G.G., McKechnie, I.
Making the most of fragments: a method for estimating shell length from fragmentary mussels (Mytilus californianus and Mytilus trossulus) on the Pacific Coast of North America
(2015) Journal of Archaeological Science 58, 175e183

DOI: 10.1016/j.jas.2015.02.029

217. Haidvogl, G., Pont, D., Dolak, H., Hohensinner, S.
Long-term evolution of fish communities in European mountainous rivers: past log driving effects, river management and species introduction (Salzach River, Danube)
(2015) Aquatic Sciences (2015) 77:395–410

DOI: 10.1007/s00027-015-0398-3

218. Thomas E., Alcázar Caicedo, C., McMichael, C.H., Corvera R., Loo, J.
Uncovering spatial patterns in the natural and human history of Brazil nut Bertholletia excelsa) across the Amazon Basin
(2015) Journal of Biogeography, 42, 1367–1382

DOI:10.1111/jbi.12540

219. Wilmshurst J.M., McGlone1 MS, Turney C.S.M
Long-term ecology resolves the timing, region of origin and process of establishment for a disputed alien tree
(2015) AoB PLANTS 7: plv104

DOI:10.1093/aobpla/plv104

220. Stahl, P. W.
Interpreting interfluvial landscape transformations in the pre-Columbian Amazon.
(2015) The Holocene, 25(10), 1598-1603.

DOI: 10.1177/0959683615588372

221. Elmhagen, B., Destouni, G., Angerbjörn, A., Borgström, S., Boyd, E., Cousins, S.A., Dalén, L., Ehrlén, J., Ermold, M., Hambäck, P.A. and Hedlund, J.,
Interacting effects of change in climate, human population, land use, and water use on biodiversity and ecosystem services.
(2015) Ecology and Society, 20(1).

222. Machado Mello AJ, Peroni N
Cultural landscapes of the Araucaria Forests in the northern plateau of Santa Catarina, Brazil.
(2015) Journal of Ethnobiology and Ethnomedicine, 11:51.

DOI: 10.1186/s13002-015-0039-x

223. Closset-Kopp D, Decocq G
Remnant Artificial Habitats as Biodiversity Islets into Forest Oceans.
(2015) Ecosystems 18: 507–519.

DOI: 10.1007/s10021-015-9843-3

224. McIntyre PJ, Thorne JH, Dolanc CR, Flint AL, Flint LE, Kelly M, Ackerly DD
Twentieth-century shifts in forest structure in California: Denser forests, smaller trees, and increased dominance of oaks
(2015) Proceedings of the National Academy of Sciences of the United States of America, 112(5):1458–1463.

DOI: 10.1073/pnas.1410186112

225. Cousins SAO, Auffret AG, Lindgren J, Tränk L
Regional-scale land-cover change during the 20th century and its consequences for biodiversity.
(2015) AMBIO 44(Suppl. 1):S17–S27.

DOI 10.1007/s13280-014-0585-9

226. Clavero M, Hermoso V
Historical data to plan the recovery of the European eel.
(2015) Journal of Applied Ecology 52, 960–968

DOI: 10.1111/1365-2664.12446

227. Hutchinson WF, Culling M, Orton DC, Hänfling B, Handley, LL, Hamilton-Dyer S, O'Connell, TC, Richards MP, Barrett JH
The globalization of naval provisioning: Ancient DNA and stable isotope analyses of stored cod from the wreck of the Mary Rose, AD 1545.
(2015) Royal Society Open Science Volume 2, Issue 9, Article number 150199.

DOI: 10.1098/rsos.150199

228. Rogers TL, Kimbro DL
Causes and consequences of historical multi-trophic diversity change in an intertidal seagrass bed.
(2015) Marine Ecology Progress Series Volume 540, Pages 13 - 26.

DOI: 10.3354/meps11526

229. Coughlan, M.R.
Traditional fire-use, landscape transition, and the legacies of social theory past
(2015) Ambio, 44 (8), pp. 705-717.

DOI: 10.1007/s13280-015-0643-y

230. Thornton, T.F., Hebert, J.
Neoliberal and neo-communal herring fisheries in Southeast Alaska: Reframing sustainability in marine ecosystems
(2015) Marine Policy, 61, pp. 366-375. Cited 3 times.

DOI: 10.1016/j.marpol.2014.11.015

231. Singh, G.G., McKechnie, I., Braje, T.J., Campbell, B.
"All models are wrong but some are useful": A response to Campbell's comment on estimating Mytilus californianus shell size
(2015) Journal of Archaeological Science, 63, pp. 160-163.

DOI: 10.1016/j.jas.2015.08.021

232. Tulowiecki, S.J., Larsen, C.P.S.
Native American impact on past forest composition inferred from species distribution models, Chautauqua County, New York
(2015) Ecological Monographs, 85 (4), pp. 557-581.

DOI: 10.1890/14-2259.1.sm

233. Renard, D., Rhemtull, J.M., Bennett, E.M.
Historical dynamics in ecosystem service bundles
(2015) Proceedings of the National Academy of Sciences of the United States of America, 112 (43), pp. 13411-13416.

DOI: 10.1073/pnas.1502565112

234. Thurstan, R.H., McClenachan, L., Crowder, L.B., Drew, J.A., Kittinger, J.N., Levin, P.S., Roberts, C.M., Pandolfi, J.M.
Filling historical data gaps to foster solutions in marine conservation
(2015) Ocean and Coastal Management, 115, pp. 31-40. Cited 1 time.

DOI: 10.1016/j.ocecoaman.2015.04.019

235. Delibes, R., Delibes-Mateos, M.
Linking historical ecology and invasion biology: some lessons from European rabbit introductions into the new world before the nineteenth century
(2015) Biological Invasions, 17 (9), pp. 2505-2515.

DOI: 10.1007/s10530-015-0905-4

236. Cevasco, R., Moreno, D., Hearn, R.
Biodiversification as an historical process: an appeal for the application of historical ecology to bio-cultural diversity research
(2015) Biodiversity and Conservation, 24 (13), pp. 3167-3183. Cited 1 time.

DOI: 10.1007/s10531-015-0943-3

237. Blake, B., Ermgassen, P.S.E.Z.
The history and decline of ostrea lurida in Willapa Bay, Washington
(2015) Journal of Shellfish Research, 34 (2), pp. 273-280.

DOI: 10.2983/035.034.0208

238. Auffret, A.G., Plue, J., Cousins, S.A.O.
The spatial and temporal components of functional connectivity in fragmented landscapes
(2015) Ambio, 44 (1), pp. 51-59. Cited 1 time.

DOI: 10.1007/s13280-014-0588-6

239. Haidvogl, G., Lajus, D., Pont, D., Schmid, M., Jungwirth, M., Lajus, J.
Typology of historical sources and the reconstruction of long-term historical changes of riverine fish: A case study of the Austrian Danube and northern Russian rivers
(2015) Ecology of Freshwater Fish, 23 (4), pp. 498-515. Cited 6 times.

DOI: 10.1111/eff.12103

240. Bortolus, A., Carlton, J.T., Schwindt, E.
Reimagining South American coasts: Unveiling the hidden invasion history of an iconic ecological engineer
(2015) Diversity and Distributions, 21 (11), pp. 1267-1283.

DOI: 10.1111/ddi.12377

241. Beauséjour, R., Handa, I.T., Lechowicz, M.J., Gilbert, B., Vellend, M.
Historical anthropogenic disturbances influence patterns of non-native earthworm and plant invasions in a temperate primary forest
(2015) Biological Invasions, 17 (4), pp. 1267-1281.

DOI: 10.1007/s10530-014-0794-y

242. Parker, S.S.
Incorporating critical elements of city distinctiveness into urban biodiversity conservation
(2015) Biodiversity and Conservation, 24 (3), pp. 683-700. Cited 1 time.

DOI: 10.1007/s10531-014-0832-1

243. Lane, P.J.
Archaeology in the age of the anthropocene: A critical assessment of its scope and societal contributions
(2015) Journal of Field Archaeology, 40 (5), pp. 485-498.

DOI: 10.1179/2042458215Y.0000000022

244. Whittet, R., Hope, J., Ellis, C.J.
Open Structured Woodland and the Ecological Interpretation of Scotland’s Ancient Woodland Inventory
(2015) Scottish Geographical Journal, 131 (2), pp. 67-77.

DOI: 10.1080/14702541.2015.1007076

245. Masubelele, M.L., Hoffman, M.T., Bond, W.J.
A repeat photograph analysis of long-term vegetation change in semi-arid South Africa in response to land use and climate
(2015) Journal of Vegetation Science, 26 (5), pp. 1013-1023.

DOI: 10.1111/jvs.12303

246. Alleway, H.K., Connell, S.D.
Loss of an ecological baseline through the eradication of oyster reefs from coastal ecosystems and human memory
(2015) Conservation Biology, 29 (3), pp. 795-804. Cited 5 times.

DOI: 10.1111/cobi.12452

247. Szabó, P.
Historical ecology: Past, present and future
(2015) Biological Reviews, 90 (4), pp. 997-1014. Cited 1 time.

DOI: 10.1111/brv.12141

248. Cramer, K.L., Leonard-Pingel, J.S., Rodríguez, F., Jackson, J.B.C.
Molluscan subfossil assemblages reveal the long-term deterioration of coral reef environments in Caribbean Panama
(2015) Marine Pollution Bulletin, 96 (1-2), pp. 176-187.

DOI: 10.1016/j.marpolbul.2015.05.031

249. Hofman, C.A., Rick, T.C., Fleischer, R.C., Maldonado, J.E.
Conservation archaeogenomics: Ancient DNA and biodiversity in the Anthropocene
(2015) Trends in Ecology and Evolution, 30 (9), pp. 540-549.

DOI: 10.1016/j.tree.2015.06.008

250. Larsen, C.P.S., Tulowiecki, S.J., Wang, Y.-C., Trgovac, A.B.
Predicting historic forest composition using species lists in presettlement land survey records, western New York
(2015) Applied Vegetation Science, 18 (3), pp. 481-492.

DOI: 10.1111/avsc.12165

251. Steen-Adams, M.M., Langston, N., Adams, M.D.O., Mladenoff, D.J.

Historical framework to explain long-term coupled human and natural system feedbacks: Application to a multiple-ownership forest landscape in the Northern Great Lakes region, USA
(2015) Ecology and Society, 20 (1), art. no. 28, . Cited 1 time.

DOI: 10.5751/ES-06930-200128

252. McClenachan, L., Lovell, S., Keaveney, C.
Social benefits of restoring historical ecosystems and fisheries: Alewives in Maine
(2015) Ecology and Society, 20 (2), 11 p.

DOI: 10.5751/ES-07585-200231

253. Dallimer, M., Davies, Z.G., Diaz-Porras, D.F., Irvine, K.N., Maltby, L., Warren, P.H., Armsworth, P.R., Gaston, K.J.
Historical influences on the current provision of multiple ecosystem services
(2015) Global Environmental Change, 31, pp. 307-317. Cited 1 time.

DOI: 10.1016/j.gloenvcha.2015.01.015

254. Armstrong, CG, Veteto, JR
Historical Ecology and Ethnobiology: Applied Research for Environmental Conservation and Social Justice.
(2015) Ethnobiology Letters 6(1):5-7.

DOI: 10.14237/ebl.6.1.2015.313.

255. Tulowiecki, S.J., Larsen, C.P.S., Wang, Y.-C.
Effects of positional error on modeling species distributions: a perspective using presettlement land survey records
(2015) Plant Ecology, 216 (1), pp. 67-85. Cited 1 time.

DOI: 10.1007/s11258-014-0417-9

2014

256. Haidvogl, G., Lajus, D., Pont, D., Schmid, M., Jungwirth, M., Lajus, J.
Typology of historical sources and the reconstruction of long-term historical changes of riverine fish: a case study of the Austrian Danube and northern Russian rivers
(2014) Ecology of Freshwater Fish 2014: 23: 498–515

DOI: 10.1111/eff.12103

257. Umbanhowar, C. E. (2014). Charles A. Geyer Agricultural Botanical Survey of 1838–1839: a comparison of the composition of Minnesota prairies then and now. Ecological Restoration, 32(1), 16-27.

DOI: 10.3368/er.32.1.16

258. Eriksson, O., & Cousins, S. A.
Historical landscape perspectives on grasslands in Sweden and the Baltic region.
(2014) Land, 3(1), 300-321.

DOI: 10.3390/land3010300

259. Lajus, D., Glazkova, J., Sendek, D., Khaitov, V., Lajus, J.
Dynamics of fish catches in the eastern Gulf of Finland (Baltic Sea) and downstream of the Neva River during the 20th century
(2014) Aquatic Sciences, 77 (3), pp. 411-425.

DOI: 10.1007/s00027-014-0389-9

260. Tulowiecki, S.J.
Using vegetation data within presettlement land survey records for species distribution modeling: A tale of two datasets
(2014) Ecological Modelling, 291, pp. 109-120. Cited 3 times.

DOI: 10.1016/j.ecolmodel.2014.07.025

261. Marchant, R., Lane, P.
Past perspectives for the future: Foundations for sustainable development in East Africa
(2014) Journal of Archaeological Science, 51, pp. 12-21. Cited 2 times.

DOI: 10.1016/j.jas.2013.07.005

262. Yeakel, J.D., Pires, M.M., Rudolf, L., Dominy, N.J., Koch, P.L., Guimarães, P.R., Gross, T.
Collapse of an ecological network in Ancient Egypt
(2014) Proceedings of the National Academy of Sciences of the United States of America, 111 (40), pp. 14475-14477. Cited 8 times.

DOI: 10.1073/pnas.1408471111

263. Fortuny, X., Carcaillet, C., Chauchard, S.
Land use legacies and site variables control the understorey plant communities in Mediterranean broadleaved forests
(2014) Agriculture, Ecosystems and Environment, 189, pp. 53-59. Cited 1 time.

DOI: 10.1016/j.agee.2014.03.012

264. Maxwell, R.S., Taylor, A.H., Skinner, C.N., Safford, H.D., Isaacs, R.E., Airey, C., Young, A.B.
Landscape-scale modeling of reference period forest conditions and fire behavior on heavily logged lands
(2014) Ecosphere, 5 (3), art. no. 32, . Cited 2 times.

DOI: 10.1890/ES13-00294.1

265. Eddy, T.D., Pitcher, T.J., MacDiarmid, A.B., Byfield, T.T., Tam, J.C., Jones, T.T., Bell, J.J., Gardner, J.P.A.
Lobsters as keystone: Only in unfished ecosystems?
(2014) Ecological Modelling, 275, pp. 48-72. Cited 6 times.

DOI: 10.1016/j.ecolmodel.2013.12.006

266. McKechnie, I., Lepofsky, D., Moss, M.L., Butler, V.L., Orchard, T.J., Coupland, G., Foster, F., Caldwell, M., Lertzman, K.

Archaeological data provide alternative hypotheses on Pacific herring (Clupea pallasii) distribution, abundance, and variability
(2014) Proceedings of the National Academy of Sciences of the United States of America, 111 (9), pp. E807-E816. Cited 9 times.

DOI: 10.1073/pnas.1316072111

267. Boucher, Y., Grondin, P., Auger, I.
Land use history (1840-2005) and physiography as determinants of southern boreal forests
(2014) Landscape Ecology, 29 (3), pp. 437-450. Cited 2 times.

DOI: 10.1007/s10980-013-9974-x

268. Schmidt, M.J., Rapp Py-Daniel, A., de Paula Moraes, C., Valle, R.B.M., Caromano, C.F., Texeira, W.G., Barbosa, C.A., Fonseca, J.A., Magalhães, M.P., Silva do Carmo Santos, D., da Silva e Silva, R., Guapindaia, V.L., Moraes, B., Lima, H.P., Neves, E.G., Heckenberger, M.J.
Dark earths and the human built landscape in Amazonia: A widespread pattern of anthrosol formation
(2014) Journal of Archaeological Science, 42 (1), pp. 152-165. Cited 11 times.

DOI: 10.1016/j.jas.2013.11.002

269. Van Houtan, K.S., Kittinger, J.N.
Historical commercial exploitation and the current status of Hawaiian green turtles
(2014) Biological Conservation, 170, pp. 20-27. Cited 3 times.

DOI: 10.1016/j.biocon.2013.11.011

270. Coughlan, M.R.
Farmers, flames, and forests: Historical ecology of pastoral fire use and landscape change in the French Western Pyrenees, 1830-2011
(2014) Forest Ecology and Management, 312, pp. 55-66. Cited 2 times.

DOI: 10.1016/j.foreco.2013.10.021

271. Thomas, F.R.
Shellfish Gathering and Conservation on Low Coral Islands: Kiribati Perspectives
(2014) Journal of Island and Coastal Archaeology, 9 (2), pp. 203-218. Cited 5 times.

DOI: 10.1080/15564894.2014.921959

272. Fraser, J.A., Leach, M., Fairhead, J.
Anthropogenic Dark Earths in the Landscapes of Upper Guinea, West Africa: Intentional or Inevitable?
(2014) Annals of the Association of American Geographers, 104 (6), pp. 1222-1238. Cited 3 times.

DOI: 10.1080/00045608.2014.941735

273. Mölder, A., Gürlich, S., Engel, F.
The distribution of endangered saproxylic beetles in Schleswig-holstein (northern Germany) as influenced by forest history and land tenure [Die

Verbreitung von gefährdeten holz bewohnenden Käfern in Schleswig-Holstein unter dem Einfluss von Forstgeschichte und Besitzstruktur]
(2014) Forstarchiv, 85 (3), pp. 84-101. Cited 2 times.

DOI: 10.4432/0300-4112-85-84

274. Millar, C.I., Heckman, K., Swanston, C., Schmidt, K., Westfall, R.D., Delany, D.L.
Radiocarbon dating of American pika fecal pellets provides insights into population extirpations and climate refugia
(2014) Ecological Applications, 24 (7), pp. 1748-1768.

275. Garbarino, M., Sibona, E., Lingua, E., Motta, R.
Decline of traditional landscape in a protected area of the southwestern Alps: The fate of enclosed pasture patches in the land mosaic shift
(2014) Journal of Mountain Science, 11 (2), pp. 544-554. Cited 7 times.

DOI: 10.1007/s11629-013-2666-9

276. Morris, L.R., Rowe, R.J.
Historical land use and altered habitats in the Great Basin
(2014) Journal of Mammalogy, 95 (6), pp. 1144-1156. Cited 3 times.

DOI: 10.1644/13-MAMM-S-169

277. Buisson, E., Bravet, P., Mieusset, J., Baillot, N., Dubreucq, C., Sadonès, L., Chenot, J., Brun, L.
Plant communities of a coastal lagoon foredune: Definition of the reference and restoration after compaction
(2014) Acta Botanica Gallica, 161 (3), pp. 277-286. Cited 2 times.

DOI: 10.1080/12538078.2014.919875

278. Rowe, R.J., Matocq, M.D.
Great Basin mammalian diversity through time
(2014) Journal of Mammalogy, 95 (6), pp. 1087-1089.

DOI: 10.1644/14-MAMM-S-213

279. Mcmichael, C.H., Palace, M.W., Golightly, M.
Bamboo-dominated forests and pre-Columbian earthwork formations in south-western Amazonia
(2014) Journal of Biogeography, 41 (9), pp. 1733-1745. Cited 5 times.

DOI: 10.1111/jbi.12325

280. Rick, T.C., Sillett, T.S., Ghalambor, C.K., Hofman, C.A., Ralls, K., Anderson, R.S., Boser, C.L., Braje, T.J., Cayan, D.R., Chesser, R.T., Collins, P.W., Erlandson, J.M., Faulkner, K.R., Fleischer, R., Funk, W.C., Galipeau, R., Huston, A., King, J., Laughrin, L., Maldonado, J., McEachern, K., Muhs, D.R., Newsome, S.D., Reeder-Myers, L., Still, C., Morrison, S.A.
Ecological change on California's channel Islands from the pleistocene to the anthropocene
(2014) BioScience, 64 (8), pp. 680-692. Cited 4 times.

DOI: 10.1093/biosci/biu094

281. Fahey, R.T., Lorimer, C.G.
Habitat associations and 150 years of compositional change in white pine-hemlock-hardwood forests based on resurvey of public land survey corners
(2014) Journal of the Torrey Botanical Society, 141 (4), pp. 277-293.

DOI: 10.3159/TORREY-D-13-00059.1

282. Lindborg, R., Plue, J., Andersson, K., Cousins, S.A.O.
Function of small habitat elements for enhancing plant diversity in different agricultural landscapes
(2014) Biological Conservation, 169, pp. 206-213. Cited 7 times.

DOI: 10.1016/j.biocon.2013.11.015

283. Munoz, S.E., Mladenoff, D.J., Schroeder, S., Williams, J.W.
Defining the spatial patterns of historical land use associated with the indigenous societies of eastern North America
(2014) Journal of Biogeography, 41 (12), pp. 2195-2210. Cited 4 times.

DOI: 10.1111/jbi.12386

284. Smith, C.B., Ebert, C.E., Kennett, D.J.
Human Ecology of Shellfish Exploitation at a Prehistoric Fishing-Farming Village on the Pacific Coast of Mexico
(2014) Journal of Island and Coastal Archaeology, 9 (2), pp. 183-202. Cited 2 times.

DOI: 10.1080/15564894.2014.881935

285. Pawlowicz, M., Stoetzel, J., Macko, S.
Environmental archaeology at Mikindani, Tanzania: Towards a historical ecology of the southern Swahili coast
(2014) Journal of African Archaeology, 12 (2), pp. 119-139.

DOI: 10.3213/2191-5784-10260

286. Walshe, K.
Archaeological Evidence for a Sealer's and Wallaby Hunter's Skinning Site on Kangaroo Island, South Australia
(2014) Journal of Island and Coastal Archaeology, 9 (1), pp. 130-143. Cited 1 time.

DOI: 10.1080/15564894.2013.834010

287. Garbarino, M., Lingua, E., Marzano, R., Urbinati, C., Bhuju, D., Carrer, M.
Human interactions with forest landscape in the Khumbu valley, Nepal
(2014) Anthropocene, 6, pp. 39-47. Cited 3 times.

DOI: 10.1016/j.ancene.2014.05.004

288. Fahey, R.T., Maurer, D.A., Bowles, M.L., McBride, J.
Evaluating restoration baselines for historically fire-protected woodlands within a Northeastern Illinois prairie peninsula landscape
(2014) Natural Areas Journal, 34 (2), pp. 166-177. Cited 2 times.

DOI: 10.3375/043.034.0206

289. Dolanc, C.R., Safford, H.D., Dobrowski, S.Z., Thorne, J.H.
Twentieth century shifts in abundance and composition of vegetation types of the Sierra Nevada, CA, US
(2014) Applied Vegetation Science, 17 (3), pp. 442-455. Cited 8 times.

DOI: 10.1111/avsc.12079

290. Thurstan, R.H., Hawkins, J.P., Roberts, C.M.
Origins of the bottom trawling controversy in the British Isles: 19th century witness testimonies reveal evidence of early fishery declines
(2014) Fish and Fisheries, 15 (3), pp. 506-522. Cited 4 times.

DOI: 10.1111/faf.12034

291. Tomscha, S.A., Gergel, S.E.
Historic land surveys present opportunities for reconstructing frontier settlement patterns in North America
(2014) Landscape Ecology, 30 (2), pp. 203-213.

DOI: 10.1007/s10980-014-0124-x

292. Thurstan, R.H., Roberts, C.M.
The past and future of fish consumption: Can supplies meet healthy eating recommendations?
(2014) Marine Pollution Bulletin, 89 (1-2), pp. 5-11. Cited 1 time.

DOI: 10.1016/j.marpolbul.2014.09.016

293. De Keersmaeker, L., Onkelinx, T., De Vos, B., Rogiers, N., Vandekerkhove, K., Thomaes, A., De Schrijver, A., Hermy, M., Verheyen, K.
The analysis of spatio-temporal forest changes (1775–2000) in Flanders (northern Belgium) indicates habitat-specific levels of fragmentation and area loss
(2014) Landscape Ecology, 30 (2), pp. 247-259.

DOI: 10.1007/s10980-014-0119-7

294. Reitalu, T., Kuneš, P., Giesecke, T.
Closing the gap between plant ecology and Quaternary palaeoecology
(2014) Journal of Vegetation Science, 25 (5), pp. 1188-1194. Cited 3 times.

DOI: 10.1111/jvs.12187

295. Walsh, K., Attema, P., De Haas, T.
The Pontine Marshes (Central Italy): A case study in wetland historical ecology
(2014) Babesch, 89 (1), pp. 27-46. Cited 1 time.

DOI: 10.2143/BAB.89.0.3034668

296. de Keersmaeker, L., Onkelinx, T., Vandekerkhove, K., Thomaes, A., Hermy, M., Verheyen, K.
A spatially explicit empirical model on actual and potential ancient forest plant diversity in a fragmented landscape
(2014) Landscape and Urban Planning, 130 (1), pp. 149-158.

DOI: 10.1016/j.landurbplan.2014.07.006

297. Roos, C.I., Bowman, D.M.J.S., Balch, J.K., Artaxo, P., Bond, W.J., Cochrane, M., D'Antonio, C.M., Defries, R., Mack, M., Johnston, F.H., Krawchuk, M.A., Kull, C.A., Moritz, M.A., Pyne, S., Scott, A.C., Swetnam, T.W.
Pyrogeography, historical ecology, and the human dimensions of fire regimes
(2014) Journal of Biogeography, 41 (4), pp. 833-836. Cited 4 times.

DOI: 10.1111/jbi.12285

298. Lobanova, T.V., Kardash, O.V.
Subsistence and Ritual Practices At The Polui Fortified Settlement, Western Siberia (Based on Fauna Remains)
(2014) Archaeology, Ethnology and Anthropology of Eurasia, 42 (3), art. no. 496, pp. 66-79.

DOI: 10.1016/j.aeae.2015.04.008

299. Leblan, V.
The Impact of West African Trade on the Distribution of Chimpanzee and Elephant Populations (Guinea, Guinea-Bissau, Senegal, 19th-20th Century)
(2014) Human Ecology, 42 (3), pp. 455-465.

DOI: 10.1007/s10745-014-9654-8

300. Green, P.T.
Mammal extinction by introduced infectious disease on Christmas Island (Indian Ocean): The historical context
(2014) Australian Zoologist, 37 (1), pp. 1-14.

DOI: 10.7882/AZ.2013.011

301. Millar, C.I.
Historic Variability: Informing Restoration Strategies, Not Prescribing Targets
(2014) Journal of Sustainable Forestry, 33 (SUP1), pp. S28-S42. Cited 4 times.

DOI: 10.1080/10549811.2014.887474

302. Lawres, N. R.
Reconceptualizing the Landscape: Changing Patterns of Land Use in a Coalescent Culture.
(2014) Journal of Anthropological Research, 70(4), 543-572.

2013

303. Gomes, T. C.
Novel ecosystems in the restoration of cultural landscapes of Tl’chés, West Chatham Island, British Columbia, Canada.
(2013) Ecological Processes, 2(1), 1-13.

304. McCune, J.L., Pellatt, M.G., Vellend, M.
Multidisciplinary synthesis of long-term human-ecosystem interactions: A perspective from the Garry oak ecosystem of British Columbia
(2013) Biological Conservation, 166, pp. 293-300. Cited 7 times.

DOI: 10.1016/j.biocon.2013.08.004

305. Barthel, S., Crumley, C., Svedin, U.
Bio-cultural refugia-Safeguarding diversity of practices for food security and biodiversity
(2013) Global Environmental Change, 23 (5), pp. 1142-1152. Cited 15 times.

DOI: 10.1016/j.gloenvcha.2013.05.001

306. Thurstan, R.H., Hawkins, J.P., Raby, L., Roberts, C.M.
Oyster (Ostrea edulis) extirpation and ecosystem transformation in the Firth of Forth, Scotland
(2013) Journal for Nature Conservation, 21 (5), pp. 253-261. Cited 7 times.

DOI: 10.1016/j.jnc.2013.01.004

307. Coverdale, T.C., Bertness, M.D., Altieri, A.H.
Regional ontogeny of new england salt marsh die-off
(2013) Conservation Biology, 27 (5), pp. 1041-1048. Cited 6 times.

DOI: 10.1111/cobi.12052

308. Baisre, J.A.
Shifting baselines and the extinction of the caribbean monk seal
(2013) Conservation Biology, 27 (5), pp. 927-935. Cited 4 times.

DOI: 10.1111/cobi.12107

309. Mcclenachan, L., Kittinger, J.N.
Multicentury trends and the sustainability of coral reef fisheries in Hawai'i and Florida
(2013) Fish and Fisheries, 14 (3), pp. 239-255. Cited 12 times.

DOI: 10.1111/j.1467-2979.2012.00465.x

310. Vellend, M., Brown, C.D., Kharouba, H.M., Mccune, J.L., Myers-Smith, I.H.
Historical ecology: Using unconventional data sources to test for effects of global environmental change
(2013) American Journal of Botany, 100 (7), pp. 1294-1305. Cited 24 times.

DOI: 10.3732/ajb.1200503

311. Clavero, M., Delibes, M.
Using historical accounts to set conservation baselines: The case of Lynx species in Spain
(2013) Biodiversity and Conservation, 22 (8), pp. 1691-1702. Cited 4 times.

DOI: 10.1007/s10531-013-0506-4

312. Szpak, P., Orchard, T.J., Salomon, A.K., Gröcke, D.R.
Regional ecological variability and impact of the maritime fur trade on nearshore ecosystems in southern Haida Gwaii (British Columbia, Canada): Evidence from stable isotope analysis of rockfish (Sebastes spp.) bone collagen
(2013) Archaeological and Anthropological Sciences, 5 (2), pp. 159-182. Cited 4 times.

DOI: 10.1007/s12520-013-0122-y

313. zu Ermgassen, P.S.E., Gray, M.W., Langdon, C.J., Spalding, M.D., Brumbaugh, R.D.
Quantifying the historic contribution of Olympia oysters to filtration in Pacific Coast (USA) estuaries and the implications for restoration objectives
(2013) Aquatic Ecology, 47 (2), pp. 149-161. Cited 2 times.

DOI: 10.1007/s10452-013-9431-6

314. Miller, J.H., Druckenmiller, P., Bahn, V.
Antlers on the Arctic Refuge: Capturing multi-generational patterns of calving ground use from bones on the landscape
(2013) Proceedings of the Royal Society B: Biological Sciences, 280 (1759), . Cited 6 times.

DOI: 10.1098/rspb.2013.0275

315. Coughlan, M.R., Petty, A.M.
Fire as a dimension of historical ecology: A response to Bowman et al. (2011)
(2013) Journal of Biogeography, 40 (5), pp. 1010-1012. Cited 1 time.

DOI: 10.1111/j.1365-2699.2012.02767.x

316. Szabó, P., Hédl, R.
Socio-Economic Demands, Ecological Conditions and the Power of Tradition: Past Woodland Management Decisions in a Central European Landscape
(2013) Landscape Research, 38 (2), pp. 243-261. Cited 8 times.

DOI: 10.1080/01426397.2012.677022

317. Plue, J., Cousins, S.A.O.
Temporal dispersal in fragmented landscapes
(2013) Biological Conservation, 160, pp. 250-262. Cited 9 times.

DOI: 10.1016/j.biocon.2013.02.010

318. Coughlan, M.R.
Errakina: Pastoral fire use and landscape memory in the basque region of the French western pyrenees
(2013) Journal of Ethnobiology, 33 (1), pp. 86-104. Cited 1 time.

DOI: 10.2993/0278-0771-33.1.86

319. Nogué, S., de Nascimento, L., Fernández-Palacios, J.M., Whittaker, R.J., Willis, K.J.
The ancient forests of La Gomera, Canary Islands, and their sensitivity to environmental change
(2013) Journal of Ecology, 101 (2), pp. 368-377. Cited 10 times.

DOI: 10.1111/1365-2745.12051

320. Drew, J., Kaufman, L.
Functional endemism: Population connectivity, shifting baselines, and the scale of human experience
(2013) Ecology and Evolution, 3 (2), pp. 450-456. Cited 1 time.

DOI: 10.1002/ece3.446

321. Rick, T.C., Lockwood, R.
Integrating Paleobiology, Archeology, and History to Inform Biological Conservation
(2013) Conservation Biology, 27 (1), pp. 45-54. Cited 30 times.

DOI: 10.1111/j.1523-1739.2012.01920.x

322. Willis, T.V., Wilson, K.A., Alexander, K.E., Leavenworth, W.B.
Tracking cod diet preference over a century in the northern gulf of maine: Historic data and modern analysis
(2013) Marine Ecology Progress Series, 474, pp. 263-276. Cited 1 time.

DOI: 10.3354/meps10068

323. Reedy-Maschner, K.L., Maschner, H.D.G.
Sustaining Sanak Island, Alaska: A cultural land trust
(2013) Sustainability (Switzerland), 5 (10), pp. 4406-4427. Cited 1 time.

DOI: 10.3390/su5104406

324. Garbarino, M., Lingua, E., Weisberg, P.J., Bottero, A., Meloni, F., Motta, R.
Land-use history and topographic gradients as driving factors of subalpine Larix decidua forests
(2013) Landscape Ecology, 28 (5), pp. 805-817. Cited 10 times.

DOI: 10.1007/s10980-012-9792-6

325. Erlandson, J.M., Braje, T.J.
Archeology and the anthropocene
(2013) Anthropocene, 4, pp. 1-7. Cited 5 times.

DOI: 10.1016/j.ancene.2014.05.003

326. Jamrichová, E., Szabó, P., Hédl, R., Kuneš, P., Bobek, P., Pelánková, B.
Continuity and change in the vegetation of a Central European oakwood
(2013) Holocene, 23 (1), pp. 46-56. Cited 12 times.

DOI: 10.1177/0959683612450200

327. Labbe, T., Adams, A., Conrad, R.
Historical condition and change in riparian vegetation, hood canal and eastern strait of Juan de Fuca, Washington
(2013) Northwest Science, 87 (1), pp. 24-39.

DOI: 10.3955/046.087.0103

328. Gimmi, U., Poulter, B., Wolf, A., Portner, H., Weber, P., Bürgi, M.
Soil carbon pools in Swiss forests show legacy effects from historic forest litter raking
(2013) Landscape Ecology, 28 (5), pp. 835-846. Cited 11 times.

DOI: 10.1007/s10980-012-9778-4

329. zu Ermgassen, P.S.E., Spalding, M.D., Grizzle, R.E., Brumbaugh, R.D.

Quantifying the Loss of a Marine Ecosystem Service: Filtration by the Eastern Oyster in US Estuaries
(2013) Estuaries and Coasts, 36 (1), pp. 36-43. Cited 29 times.

DOI: 10.1007/s12237-012-9559-y

330. Dega, M., Latinis, D.K.
The social and ecological trajectory of prehistoric Cambodian earthworks
(2013) Asian Perspectives, 52 (2), pp. 327-346.

DOI: 10.1353/asi.2013.0008

331. Rick, T.C., Kirch, P.V., Erlandson, J.M., Fitzpatrick, S.M.
Archeology, deep history, and the human transformation of island ecosystems
(2013) Anthropocene, 4, pp. 33-45. Cited 13 times.

DOI: 10.1016/j.ancene.2013.08.002

332. Öllerer, K.
The vegetation of the breite wood-pasture (Sighişoara, Romania)-history, current status and prospects
(2013) Brukenthal. Acta Musei, 8 (3), pp. 547-566. Cited 2 times.

333. Murphy, S.A.
Buddhism and its relationship to Dvaravati period settlement patterns and material culture in Northeast Thailand and central laos c. Sixth-eleventh centuries A.D.: A historical ecology approach to the landscape of the Khorat plateau
(2013) Asian Perspectives, 52 (2), pp. 300-326.

DOI: 10.1353/asi.2013.0017

334. Schmidt, M.
Amazonian dark earths: Pathways to sustainable development in tropical rainforests?
(2013) Boletim do Museu Paraense Emilio Goeldi:Ciencias Humanas, 8 (1), pp. 11-38. Cited 4 times.

DOI: 10.1590/S1981-81222013000100002

2012

335. Szymura, T.H.
How does recent vegetation reflects previous systems of forest management?
(2012) Polish Journal of Ecology, 60 (4), pp. 859-862. Cited 7 times.

336. Szabó, P.
Sources and methods to reconstruct past masting patterns in European oak species
(2012) Arboricultural Journal, 34 (4), pp. 203-214. Cited 2 times.

DOI: 10.1080/03071375.2012.749117

337. Forey, E., Dutoit, T.
Vegetation, soils and seed banks of limestone grasslands are still impacted by former cultivation one century after abandonment
(2012) Community Ecology, 13 (2), pp. 194-202. Cited 6 times.

DOI: 10.1556/ComEc.13.2012.2.9

338. Leblan, V.
Contribution to landscape history in West Africa. Explorers' and European residents' northern rivers of the 1830-1910 period [Contribution à l'histoire des paysages en Afrique de l'Ouest : Les Rivières du Sud des explorateurs et des résidents européens de la période 1830-1910]
(2012) Cahiers d'Etudes Africaines, 52 (4), pp. 937-973. Cited 1 time.

339. Hanberry, B.B., Palik, B.J., He, H.S.
Comparison of historical and current forest surveys for detection of homogenization and mesophication of Minnesota forests
(2012) Landscape Ecology, 27 (10), pp. 1495-1512. Cited 12 times.

DOI: 10.1007/s10980-012-9805-5

340. Auffret, A.G., Schmucki, R., Reimark, J., Cousins, S.A.
Grazing networks provide useful functional connectivity for plants in fragmented systems
(2012) Journal of Vegetation Science, 23 (5), pp. 970-977. Cited 14 times.

DOI: 10.1111/j.1654-1103.2012.01413.x

341. Aggemyr, E., Cousins, S.A.O.
Landscape structure and land use history influence changes in island plant composition after 100years
(2012) Journal of Biogeography, 39 (9), pp. 1645-1656. Cited 11 times.

DOI: 10.1111/j.1365-2699.2012.02733.x

342. Thornton, T.F., Scheer, A.M.
Collaborative engagement of local and traditional knowledge and science in marine environments: A review
(2012) Ecology and Society, 17 (3), . Cited 29 times.

343. Coughlan, M.R., Petty, A.M.
Linking humans and fire: A proposal for a transdisciplinary fire ecology
(2012) International Journal of Wildland Fire, 21 (5), pp. 477-487. Cited 9 times.

DOI: 10.1071/WF11048

344. Cherry, J.F., Ryzewski, K., Leppard, T.P.
Multi-Period Landscape Survey and Site Risk Assessment on Montserrat, West Indies
(2012) Journal of Island and Coastal Archaeology, 7 (2), pp. 282-302. Cited 5 times.

DOI: 10.1080/15564894.2011.611857

345. Hayward, G.D., Veblen, T.T., Suring, L.H., Davis, B.
Challenges in the Application of Historical Range of Variation to Conservation and Land Management
(2012) Historical Environmental Variation in Conservation and Natural Resource Management, pp. 32-45. Cited 2 times.

DOI: 10.1002/9781118329726.ch3

346. Safford, H.D., Wiens, J.A., Hayward, G.D.
The Growing Importance of the Past in Managing Ecosystems of the Future
(2012) Historical Environmental Variation in Conservation and Natural Resource Management, pp. 319-327. Cited 6 times.

DOI: 10.1002/9781118329726.ch24

347. Veblen, T.T., Romme, W.H., Regan, C.
Regional Application of Historical Ecology at Ecologically Defined Scales: Forest Ecosystems in the Colorado Front Range
(2012) Historical Environmental Variation in Conservation and Natural Resource Management, pp. 149-165.

DOI: 10.1002/9781118329726.ch10

348. Romme, W.H., Wiens, J.A., Safford, H.D.
Setting the Stage: Theoretical and Conceptual Background of Historical Range of Variation
(2012) Historical Environmental Variation in Conservation and Natural Resource Management, pp. 3-18. Cited 5 times.

DOI: 10.1002/9781118329726.ch1

349. Hahn, B.A., Curnutt, J.L.
Using Historical Ecology to Inform Wildlife Conservation, Restoration, and Management
(2012) Historical Environmental Variation in Conservation and Natural Resource Management, pp. 205-217.

DOI: 10.1002/9781118329726.ch14

350. Padgett, W., Schrader, B., Manning, M., Tear, T.
Development of Historical Ecology Concepts and their Application to Resource Management and Conservation
(2012) Historical Environmental Variation in Conservation and Natural Resource Management, pp. 19-28.

DOI: 10.1002/9781118329726.ch2

351. Wiens, J.A., Safford, H.D., McGarigal, K., Romme, W.H., Manning, M.
What is the Scope of "History" in Historical Ecology? Issues of Scale in Management and Conservation
(2012) Historical Environmental Variation in Conservation and Natural Resource Management, pp. 63-75. Cited 3 times.

DOI: 10.1002/9781118329726.ch5

352. Safford, H.D., Hayward, G.D., Heller, N.E., Wiens, J.A.
Historical Ecology, Climate Change, and Resource Management: Can the Past Still Inform the Future?
(2012) Historical Environmental Variation in Conservation and Natural Resource Management, pp. 46-62. Cited 6 times.

DOI: 10.1002/9781118329726.ch4

353. DeMeo, T.E., Swanson, F.J., Smith, E.B., Buttrick, S.C., Kertis, J., Rice, J., Ringo, C.D., Waltz, A., Zanger, C., Friesen, C.A., Cissel, J.H.
Applying historical fire-regime concepts to forest management in the western United States: Three case studies
(2012) Historical Environmental Variation in Conservation and Natural Resource Management, pp. 194-204.

DOI: 10.1002/9781118329726.ch13

354. Kittinger, J.N., Bambico, T.M., Watson, T.K., Glazier, E.W.
Sociocultural significance of the endangered Hawaiian monk seal and the human dimensions of conservation planning
(2012) Endangered Species Research, 17 (2), pp. 139-156. Cited 1 time.

DOI: 10.3354/esr00423

355. Whitaker, A.R., Byrd, B.F.
Boat-based foraging and discontinuous prehistoric red abalone exploitation along the California coast
(2012) Journal of Anthropological Archaeology, 31 (2), pp. 196-214. Cited 11 times.

DOI: 10.1016/j.jaa.2011.12.001

356. Miller, D.D., Clarke, M., Mariani, S.
Mismatch between fish landings and market trends: A western European case study
(2012) Fisheries Research, 121-122, pp. 104-114. Cited 4 times.

DOI: 10.1016/j.fishres.2012.01.016

357. Huntley, J.W., Kaufman, D.S., Kowalewski, M., Romanek, C.S., Neves, R.J.
Sub-centennial resolution amino acid geochronology for the freshwater mussel Lampsilis for the last 2000 years
(2012) Quaternary Geochronology, 9, pp. 75-85. Cited 2 times.

DOI: 10.1016/j.quageo.2012.01.015

358. Swaney, D.P., Santoro, R.L., Howarth, R.W., Hong, B., Donaghy, K.P.
Historical changes in the food and water supply systems of the New York city metropolitan area
(2012) Regional Environmental Change, 12 (2), pp. 363-380. Cited 8 times.

DOI: 10.1007/s10113-011-0266-1

359. Cramer, K.L., Jackson, J.B.C., Angioletti, C.V., Leonard-Pingel, J., Guilderson, T.P.
Anthropogenic mortality on coral reefs in Caribbean Panama predates coral disease and bleaching
(2012) Ecology Letters, 15 (6), pp. 561-567. Cited 19 times.

DOI: 10.1111/j.1461-0248.2012.01768.x

360. Szpak, P., Orchard, T.J., McKechnie, I., Gröcke, D.R.
Historical ecology of late Holocene sea otters (Enhydra lutris) from northern British Columbia: Isotopic and zooarchaeological perspectives

(2012) Journal of Archaeological Science, 39 (5), pp. 1553-1571. Cited 14 times.

DOI: 10.1016/j.jas.2011.12.006

361. Pandolfi, J.M., Budd, A.F.
A festschrift for Jeremy B.C. Jackson and his integration of paleobiology, ecology, evolution, and conservation biology
(2012) Evolutionary Ecology, 26 (2), pp. 227-232.

DOI: 10.1007/s10682-012-9556-4

362. Boucher, Y., Grondin, P.
Impact of logging and natural stand-replacing disturbances on high-elevation boreal landscape dynamics (1950-2005) in eastern Canada
(2012) Forest Ecology and Management, 263, pp. 229-239. Cited 5 times.

DOI: 10.1016/j.foreco.2011.09.012

2011

363. Pezzi, G., Maresi, G., Conedera, M., Ferrari, C.
Woody species composition of chestnut stands in the Northern Apennines: The result of 200 years of changes in land use
(2011) Landscape Ecology, 26 (10), pp. 1463-1476. Cited 7 times.

DOI: 10.1007/s10980-011-9661-8

364. Rick, T.C., DeLong, R.L., Erlandson, J.M., Braje, T.J., Jones, T.L., Arnold, J.E., des Lauriers, M.R., Hildebrandt, W.R., Kennett, D.J., Vellanoweth, R.L., Wake, T.A.
Where were the northern elephant seals? holocene archaeology and biogeography of mirounga angustirostris
(2011) Holocene, 21 (7), pp. 1159-1166. Cited 6 times.

DOI: 10.1177/0959683611400463

365. Daoud-Bouattour, A., Muller, S.D., Jamaa, H.F.-B., Saad-Limam, S.B., Rhazi, L., Soulié-Märsche, I., Rouissi, M., Touati, B., Jilani, I.B.H., Gammar, A.M., Ghrabi-Gammar, Z.
Conservation of Mediterranean wetlands: Interest of historical approach
(2011) Comptes Rendus - Biologies, 334 (10), pp. 742-756. Cited 7 times.

DOI: 10.1016/j.crvi.2011.07.006

366. Mcclanahan, T.R., Omukoto, J.O.
Comparison of Modern and Historical Fish Catches (AD 750-1400) to Inform Goals for Marine Protected Areas and Sustainable Fisheries
(2011) Conservation Biology, 25 (5), pp. 945-955. Cited 11 times.

DOI: 10.1111/j.1523-1739.2011.01694.x

367. Steen-Adams, M.M., Mladenoff, D.J., Langston, N.E., Liu, F., Zhu, J.
Influence of biophysical factors and differences in Ojibwe reservation versus Euro-American social histories on forest landscape change in northern Wisconsin, USA
(2011) Landscape Ecology, 26 (8), pp. 1165-1178. Cited 8 times.

DOI: 10.1007/s10980-011-9630-2

368. Brister, E., Hane, E., Korfmacher, K.
Visualizing plant community change using historical records
(2011) International Journal of Applied Geospatial Research, 2 (4), pp. 1-18. Cited 3 times.

DOI: 10.4018/jagr.2011100101

369. Nuttall, M.A., Jordaan, A., Cerrato, R.M., Frisk, M.G.
Identifying 120 years of decline in ecosystem structure and maturity of Great South Bay, New York using the Ecopath modelling approach
(2011) Ecological Modelling, 222 (18), pp. 3335-3345. Cited 12 times.

DOI: 10.1016/j.ecolmodel.2011.07.004

370. Szabó, P., Hédl, R.
Advancing the Integration of History and Ecology for Conservation
(2011) Conservation Biology, 25 (4), pp. 680-687. Cited 27 times.

DOI: 10.1111/j.1523-1739.2011.01710.x

371. Fraser, J.A., Junqueira, A.B., Kawa, N.C., Moraes, C.P., Clement, C.R.
Crop Diversity on Anthropogenic Dark Earths in Central Amazonia
(2011) Human Ecology, 39 (4), pp. 395-406. Cited 7 times.

DOI: 10.1007/s10745-011-9405-z

372. Scoles, R., Gribel, R.
Population Structure of Brazil Nut (Bertholletia excelsa, Lecythidaceae) Stands in Two Areas with Different Occupation Histories in the Brazilian Amazon
(2011) Human Ecology, 39 (4), pp. 455-464. Cited 16 times.

DOI: 10.1007/s10745-011-9412-0

373. Ireland, A.W., Oswald, W.W., Foster, D.R.
An integrated reconstruction of recent forest dynamics in a New England cultural landscape
(2011) Vegetation History and Archaeobotany, 20 (4), pp. 245-252. Cited 4 times.

DOI: 10.1007/s00334-011-0287-1

374. Walker, J.H.
Amazonian Dark Earth and Ring Ditches in the Central Llanos de Mojos, Bolivia
(2011) Culture, Agriculture, Food and Environment, 33 (1), pp. 2-14. Cited 3 times.

DOI: 10.1111/j.2153-9561.2011.01043.x

375. Hoeksema, B.W., van der Land, J., van der Meij, S.E.T., van Ofwegen, L.P., Reijnen, B.T., van Soest, R.W.M., de Voogd, N.J.
Unforeseen importance of historical collections as baselines to determine biotic change of coral reefs: The Saba Bank case
(2011) Marine Ecology, 32 (2), pp. 135-141. Cited 31 times.

DOI: 10.1111/j.1439-0485.2011.00434.x

376. Filer, C.
Interdisciplinary perspectives on historical ecology and environmental policy in Papua New Guinea
(2011) Environmental Conservation, 38 (2), pp. 256-269. Cited 4 times.

DOI: 10.1017/S0376892910000913

377. Bourdages, M., Lavoie, C.
Plant introduction and extirpation in a small island park: Natural and anthropogenic rates
(2011) Ecoscience, 18 (2), pp. 89-97.

DOI: 10.2980/18-2-3388

378. Liu, F., Mladenoff, D.J., Keuler, N.S., Moore, L.S.
Broadscale variability in tree data of the historical Public Land Survey and its consequences for ecological studies
(2011) Ecological Monographs, 81 (2), pp. 259-275. Cited 16 times.

DOI: 10.1890/10-0232.1

379. Erlandson, J.M., Braje, T.J., Rick, T.C., Jew, N.P., Kennett, D.J., Dwyer, N., Ainis, A.F., Vellanoweth, R.L., Watts, J.
10,000 years of human predation and size changes in the owl limpet (Lottia gigantea) on San Miguel Island, California
(2011) Journal of Archaeological Science, 38 (5), pp. 1127-1134. Cited 21 times.

DOI: 10.1016/j.jas.2010.12.009

380. Sitzia, T., Trentanovi, G.
Maggengo meadow patches enclosed by forests in the Italian Alps: Evidence of landscape legacy on plant diversity
(2011) Biodiversity and Conservation, 20 (5), pp. 945-961. Cited 10 times.

DOI: 10.1007/s10531-011-0006-3

381. Shepard Jr., G.H., Ramirez, H.
"Made in Brazil": Human Dispersal of the Brazil Nut (Bertholletia excelsa, Lecythidaceae) in Ancient Amazonia
(2011) Economic Botany, 65 (1), pp. 44-65. Cited 28 times.

DOI: 10.1007/s12231-011-9151-6

382. Keane, R.E., Holsinger, L., Parsons, R.A.
Evaluating indices that measure departure of current landscape composition from historical conditions
(2011) USDA Forest Service - Research Paper RMRS-RP, (83 RP), pp. 1-28.

383. Ross, N.J.
Modern tree species composition reflects ancient Maya "forest gardens" in northwest Belize
(2011) Ecological Applications, 21 (1), pp. 75-84. Cited 17 times.

DOI: 10.1890/09-0662.1

384. Hall, C.J., Jordaan, A., Frisk, M.G.
The historic influence of dams on diadromous fish habitat with a focus on river herring and hydrologic longitudinal connectivity
(2011) Landscape Ecology, 26 (1), pp. 95-107. Cited 40 times.

DOI: 10.1007/s10980-010-9539-1

385. Wulf, M., Rujner, H.
A GIS-based method for the reconstruction of the late eighteenth century forest vegetation in the Prignitz region (NE Germany)
(2011) Landscape Ecology, 26 (2), pp. 153-168. Cited 7 times.

DOI: 10.1007/s10980-010-9555-1

386. Arce-Nazario, J. A. (2011). River and Human Legacies in Amazonian Floodplain Postagricultural Forests. The Amazon Várzea: The Decade Past and the Decade Ahead, 173-185.

2010

387. Carruthers, J., & Robin, L. (2010). Taxonomic imperialism in the battles for Acacia: Identity and science in South Africa and Australia. Transactions of the Royal Society of South Africa, 65(1), 48-64.

388. Bjorkman, A.D., Vellend, M.
Defining historical baselines for conservation: Ecological changes since European settlement on Vancouver Island, Canada
(2010) Conservation Biology, 24 (6), pp. 1559-1568. Cited 22 times.

DOI: 10.1111/j.1523-1739.2010.01550.x

389. Szabó, P.
Why history matters in ecology: An interdisciplinary perspective
(2010) Environmental Conservation, 37 (4), pp. 380-387. Cited 25 times.

DOI: 10.1017/S0376892910000718

390. Gimmi, U., Wohlgemuth, T., Rigling, A., Hoffmann, C.W., Bürgi, M.
Land-use and climate change effects in forest compositional trajectories in a dry central-alpine valley
(2010) Annals of Forest Science, 67 (7), pp. 701p1-701p9. Cited 24 times.

DOI: 10.1051/forest/2010026

391. Fraser, J.A.
Caboclo Horticulture and Amazonian Dark Earths along the Middle Madeira River, Brazil
(2010) Human Ecology, 38 (5), pp. 651-662. Cited 12 times.

DOI: 10.1007/s10745-010-9338-y

392. Clement, C.R., Junqueira, A.B.
Between a Pristine Myth and an Impoverished Future
(2010) Biotropica, 42 (5), pp. 534-536. Cited 23 times.

DOI: 10.1111/j.1744-7429.2010.00674.x

393. Stump, D.
"Ancient and backward or long-lived and sustainable?" The role of the past in debates concerning rural livelihoods and resource conservation in Eastern Africa
(2010) World Development, 38 (9), pp. 1251-1262. Cited 15 times.

DOI: 10.1016/j.worlddev.2010.02.007

394. Davies, M.
A view from the east: An interdisciplinary 'Historical Ecology' approach to a contemporary agricultural landscape in Northwest Kenya
(2010) African Studies, 69 (2), pp. 279-297. Cited 2 times.

DOI: 10.1080/00020184.2010.499202

395. McKey, D., Rostain, S., Iriarte, J., Glaser, B., Birk, J.J., Holst, I., Renard, D.
Pre-Columbian agricultural landscapes, ecosystem engineers, and self-organized patchiness in Amazonia
(2010) Proceedings of the National Academy of Sciences of the United States of America, 107 (17), pp. 7823-7828. Cited 46 times.

DOI: 10.1073/pnas.0908925107

396. Dyer, J.M.
Land-use legacies in a central Appalachian forest: Differential response of trees and herbs to historic agricultural practices
(2010) Applied Vegetation Science, 13 (2), pp. 195-206. Cited 12 times.

DOI: 10.1111/j.1654-109X.2009.01061.x

397. Fraser, J.A.
The diversity of bitter manioc (Manihot Esculenta Crantz) cultivation in a whitewater Amazonian landscape
(2010) Diversity, 2 (4), pp. 586-609. Cited 8 times.

DOI: 10.3390/d2040586

398. Szabó, P.
Ancient woodland boundaries in Europe
(2010) Journal of Historical Geography, 36 (2), pp. 205-214. Cited 8 times.

DOI: 10.1016/j.jhg.2009.10.005

399. Arroyo-Kalin, M.
The Amazonian formative: Crop domestication and anthropogenic soils
(2010) Diversity, 2 (4), pp. 473-504. Cited 27 times.

DOI: 10.3390/d2040473

400. Erickson, C.L.
The transformation of environment into landscape: The historical ecology of monumental earthwork construction in the Bolivian Amazon
(2010) Diversity, 2 (4), pp. 618-652. Cited 26 times.

DOI: 10.3390/d2040619

401. Morris, L.R., Ryel, R.J., West, N.E.
Can soil phytolith analysis and charcoal be used as indicators of historic fire in the pinyon-juniper and sagebrush steppe ecosystem types of the Great Basin Desert, USA?
(2010) Holocene, 20 (1), pp. 105-114. Cited 4 times.

DOI: 10.1177/0959683609348858

402. Cormier, L.A.
The historical ecology of human and wild primate malarias in the new world
(2010) Diversity, 2 (2), pp. 256-280. Cited 3 times.

DOI: 10.3390/d2020256

403. Bürgi, M., Straub, A., Gimmi, U., Salzmann, D.
The recent landscape history of Limpach valley, Switzerland: Considering three empirical hypotheses on driving forces of landscape change
(2010) Landscape Ecology, 25 (2), pp. 287-297. Cited 12 times.

DOI: 10.1007/s10980-009-9412-2

404. Szabó, P.
Driving forces of stability and change in woodland structure: A case-study from the Czech lowlands
(2010) Forest Ecology and Management, 259 (3), pp. 650-656. Cited 28 times.

DOI: 10.1016/j.foreco.2009.11.026

405. McNeil, C.L., Burney, D.A., Burney, L.P.
Evidence disputing deforestation as the cause for the collapse of the ancient Maya polity of Copan, Honduras
(2010) Proceedings of the National Academy of Sciences of the United States of America, 107 (3), pp. 1017-1022. Cited 23 times.

DOI: 10.1073/pnas.0904760107

406. Petty, A.M., Werner, P.A.
How many buffalo does it take to change a savanna?
(2010) Journal of Biogeography, 37 (1), pp. 193-195. Cited 4 times.

DOI: 10.1111/j.1365-2699.2009.02185.x

407. Bowman, D.M.J.S., Prior, L.D., Williamson, G.
The roles of statistical inference and historical sources in understanding landscape change: The case of feral buffalo in the freshwater floodplains of Kakadu National Park
(2010) Journal of Biogeography, 37 (1), pp. 195-199. Cited 5 times.

DOI: 10.1111/j.1365-2699.2009.02244.x

2009

408. Stahl, P.W.
Adventive vertebrates and historical ecology in the pre-Columbian neotropics
(2009) Diversity, 1 (2), pp. 151-165. Cited 9 times.

DOI: 10.3390/d1020151

409. Karlík, P., Poschlod, P.
History or abiotic filter: Which is more important in determining the species composition of calcareous grasslands?
(2009) Preslia, 81 (4), pp. 321-340. Cited 27 times.

410. Keane, R.E., Hessburg, P.F., Landres, P.B., Swanson, F.J.
The use of historical range and variability (HRV) in landscape management
(2009) Forest Ecology and Management, 258 (7), pp. 1025-1037. Cited 100 times.

DOI: 10.1016/j.foreco.2009.05.035

411. Lehmann, C.E.R., Prior, L.D., Bowman, D.M.J.S.
Decadal dynamics of tree cover in an australian tropical Savanna
(2009) Austral Ecology, 34 (6), pp. 601-612. Cited 21 times.

DOI: 10.1111/j.1442-9993.2009.01964.x

412. Aswani, S., Allen, M.S.
A Marquesan coral reef (French Polynesia) in historical context: An integrated socio-ecological approach
(2009) Aquatic Conservation: Marine and Freshwater Ecosystems, 19 (6), pp. 614-625. Cited 9 times.

DOI: 10.1002/aqc.1006

413. Robichaud, W.G., Sinclair, A.R.E., Odarkor-Lanquaye, N., Klinkenberg, B.
Stable forest cover under increasing populations of swidden cultivators in central laos: The roles of intrinsic culture and extrinsic wildlife trade
(2009) Ecology and Society, 14 (1), . Cited 18 times.

414. Sciama, D., Augusto, L., Dupouey, J.-L., Gonzalez, M., Moares Domínguez, C.
Floristic and ecological differences between recent and ancient forests growing on non-acidic soils
(2009) Forest Ecology and Management, 258 (5), pp. 600-608. Cited 16 times.

DOI: 10.1016/j.foreco.2009.04.027

415. McClenachan, L.
Historical declines of goliath grouper populations in South Florida, USA
(2009) Endangered Species Research, 7 (3), pp. 175-181. Cited 29 times.

DOI: 10.3354/esr00167

416. Rhemtulla, J.M., Mladenoff, D.J., Clayton, M.K.
Legacies of historical land use on regional forest composition and structure in Wisconsin, USA (mid-1800s-1930s-2000s)
(2009) Ecological Applications, 19 (4), pp. 1061-1078. Cited 49 times.

DOI: 10.1890/08-1453.1

417. Gimmi, U., Wolf, A., Bürgi, M., Scherstjanoi, M., Bugmann, H.

Quantifying disturbance effects on vegetation carbon pools in mountain forests based on historical data
(2009) Regional Environmental Change, 9 (2), pp. 121-130. Cited 16 times.

DOI: 10.1007/s10113-008-0071-7

418. McClenachan, L.
Documenting loss of large trophy fish from the florida keys with historical photographs
(2009) Conservation Biology, 23 (3), pp. 636-643. Cited 71 times.

DOI: 10.1111/j.1523-1739.2008.01152.x

419. Hardt, M.J.
Lessons from the past: The collapse of Jamaican coral reefs
(2009) Fish and Fisheries, 10 (2), pp. 143-158. Cited 15 times.

DOI: 10.1111/j.1467-2979.2008.00308.x

420. MacDonald, G.K., Bennett, E.M.
Phosphorus accumulation in saint lawrence river watershed soils: A century-long perspective
(2009) Ecosystems, 12 (4), pp. 621-635. Cited 20 times.

DOI: 10.1007/s10021-009-9246-4

421. Szabó, P.
Open woodland in Europe in the Mesolithic and in the Middle Ages: Can there be a connection?
(2009) Forest Ecology and Management, 257 (12), pp. 2327-2330. Cited 15 times.

DOI: 10.1016/j.foreco.2009.03.035

422. Tunin-Ley, A., Ibañez, F., Labat, J.-P., Zingone, A., Lemée, R.
Phytoplankton biodiversity and NW Mediterranean Sea warming: Changes in the dinoflagellate genus Ceratium in the 20th century
(2009) Marine Ecology Progress Series, 375, pp. 85-99. Cited 15 times.

DOI: 10.3354/meps07730

423. Marshall, P.
The problem of eastern white pine (Pinus strobus L.) in Southern New England: Ecophysiology, site restriction, and historical land-use change
(2009) Journal of Sustainable Forestry, 28 (1-2), pp. 108-131.

DOI: 10.1080/10549810802626423

424. López Sáez JA, López Merino L, Alba Sánchez F, Pérez Díaz S
Contribución paleoambiental al estudio de la trashumancia en el sector abulense de la Sierra de Gredos.
(2009) HISPANIA. Revista Española de Historia 69(231):9-38.

425. Dujovny, E.
The deepest cut: Political ecology in the dredging of a new sea mouth in Chilika Lake, Orissa, India.
(2009) Conservation and Society, 7(3), 192-204.

2008

426. Roosevelt, A.C.
AMERICAS, SOUTH | Amazon Basin
(2008) Encyclopedia of Archaeology, pp. 343-357

DOI: 10.1016/B978-012373962-9.00004-2

427. Ashmore, W., Blackmore, C.
LANDSCAPE ARCHAEOLOGY
(2008) Encyclopedia of Archaeology, pp. 1569-1578

DOI: 10.1016/B978-012373962-9.00170-9

428. Bürgi, M.
Historical ecology - An interdisciplinary approach, exemplified by historical use of forest litter [Historische ökologie - Ein interdisziplinärer forschungsansatz, illustriert am beispiel der waldstreunutzung]
(2008) GAIA, 17 (4), pp. 370-377. Cited 2 times.

429. Moreno, D., Montanari, C.
Beyond perception: Towards a historical ecology of rural landscape in Italy [Más alla de la percepción: hacia una ecología histórica del paisaje rural en italia]
(2008) Cuadernos Geograficos, (43), pp. 29-49. Cited 4 times.

430. Bürgi, M. (2008). Gedanken zum Weltagrarbericht aus historisch-ökologischer Sicht. GAIA-Ecological Perspectives for Science and Society, 17(3), 293-294.

431. Voelker, G., Outlaw, R.K.
Establishing a perimeter position: Speciation around the Indian Ocean Basin
(2008) Journal of Evolutionary Biology, 21 (6), pp. 1779-1788. Cited 11 times.

DOI: 10.1111/j.1420-9101.2008.01588.x

432. Waldron, J.L., Welch, S.M., Bennett, S.H.
Vegetation structure and the habitat specificity of a declining North American reptile: A remnant of former landscapes
(2008) Biological Conservation, 141 (10), pp. 2477-2482. Cited 18 times.

DOI: 10.1016/j.biocon.2008.07.008

433. Sáenz-Arroyo, A., Roberts, C.M.
Consilience in fisheries science
(2008) Fish and Fisheries, 9 (3), pp. 316-327. Cited 4 times.

DOI: 10.1111/j.1467-2979.2008.00276.x

434. Cipriani, R., Guzman, H.M., Lopez, M.
Harvest history and current densities of the pearl oyster Pinctada mazatlanica (Bivalvia: Pteriidae) in Las Perlas and Coiba Archipelagos, Panama

(2008) Journal of Shellfish Research, 27 (4), pp. 691-700. Cited 4 times.

DOI: 10.2983/0730-8000(2008)27[691:HHACDO]2.0.CO;2

435. Dahdouh-Guebas, F., Koedam, N.
Long-term retrospection on mangrove development using transdisciplinary approaches: A review
(2008) Aquatic Botany, 89 (2), pp. 80-92. Cited 35 times.

DOI: 10.1016/j.aquabot.2008.03.012

436. Erlandson, J.M., Rick, T.C., Braje, T.J., Steinberg, A., Vellanoweth, R.L.
Human impacts on ancient shellfish: a 10,000 year record from San Miguel Island, California
(2008) Journal of Archaeological Science, 35 (8), pp. 2144-2152. Cited 64 times.

DOI: 10.1016/j.jas.2008.01.014

437. Plue, J., Hermy, M., Verheyen, K., Thuillier, P., Saguez, R., Decocq, G.
Persistent changes in forest vegetation and seed bank 1,600 years after human occupation
(2008) Landscape Ecology, 23 (6), pp. 673-688. Cited 24 times.

DOI: 10.1007/s10980-008-9229-4

438. McClenachan, L., Cooper, A.B.
Extinction rate, historical population structure and ecological role of the Caribbean monk seal
(2008) Proceedings of the Royal Society B: Biological Sciences, 275 (1641), pp. 1351-1358. Cited 38 times.

DOI: 10.1098/rspb.2007.1757

439. Anderies, J.M., Nelson, B.A., Kinzig, A.P.
Analyzing the impact of agave cultivation on famine risk in arid pre-hispanic Northern Mexico
(2008) Human Ecology, 36 (3), pp. 409-422. Cited 11 times.

DOI: 10.1007/s10745-008-9162-9

440. McEwan, R.W., McCarthy, B.C.
Anthropogenic disturbance and the formation of oak savanna in central Kentucky, USA
(2008) Journal of Biogeography, 35 (5), pp. 965-975. Cited 17 times.

DOI: 10.1111/j.1365-2699.2007.01857.x

441. Gaynor, A., McLean, I.
Landscape histories: Mapping environmental and ecological change through the landscape art of the Swan River region of Western Australia
(2008) Environment and History, 14 (2), pp. 187-204.

DOI: 10.3197/096734008X303728

442. Fitzgerald, J.M., Loeb, R.E.

Historical ecology of Inwood hill park, Manhattan, New York
(2008) Journal of the Torrey Botanical Society, 135 (2), pp. 281-293. Cited 4 times.

DOI: 10.3159/07-RA-046.1

443. Bean, W.T., Sanderson, E.W.
Using a spatially explicit ecological model to test scenarios of fire use by Native Americans: An example from the Harlem Plains, New York, NY
(2008) Ecological Modelling, 211 (3-4), pp. 301-308. Cited 5 times.

DOI: 10.1016/j.ecolmodel.2007.09.014

444. Gimmi, U., Bürgi, M., Stuber, M.
Reconstructing anthropogenic disturbance regimes in forest ecosystems: A case study from the Swiss Rhone valley
(2008) Ecosystems, 11 (1), pp. 113-124. Cited 40 times.

DOI: 10.1007/s10021-007-9111-2

445. Bowman, D.M.J.S., Boggs, G.S., Prior, L.D.
Fire maintains an Acacia aneura shrubland-Triodia grassland mosaic in central Australia
(2008) Journal of Arid Environments, 72 (1), pp. 34-47. Cited 24 times.

DOI: 10.1016/j.jaridenv.2007.04.001

446. Lavoie, C., Saint-Louis, A.
Can a small park preserve its flora? A historical study of Bic National Park, Quebec
(2008) Botany, 86 (1), pp. 26-35. Cited 7 times.

DOI: 10.1139/B07-106

2007

447. Hermy, M., & Verheyen, K.
Legacies of the past in the present-day forest biodiversity: a review of past land-use effects on forest plant species composition and diversity.
(2007) Sustainability and diversity of forest ecosystems: An interdisciplinary approach, 361-371.

448. Poulsen, B., Holm, P., MacKenzie, B.R.
A long-term (1667-1860) perspective on impacts of fishing and environmental variability on fisheries for herring, eel, and whitefish in the Limfjord, Denmark
(2007) Fisheries Research, 87 (2-3), pp. 181-195. Cited 18 times.

DOI: 10.1016/j.fishres.2007.07.014

449. Bager, M., Karnøe Søndergaard, M., MacKenzie, B.R.
The development of fisheries at Bornholm, Denmark (Baltic Sea) during 1880s-1914
(2007) Fisheries Research, 87 (2-3), pp. 146-154. Cited 9 times.

DOI: 10.1016/j.fishres.2007.08.001

450. MacKenzie, B.R., Bager, M., Ojaveer, H., Awebro, K., Heino, U., Holm, P., Must, A.
Multi-decadal scale variability in the eastern Baltic cod fishery 1550-1860-Evidence and causes
(2007) Fisheries Research, 87 (2-3), pp. 106-119. Cited 17 times.

DOI: 10.1016/j.fishres.2007.07.003

451. Braje, T.J., Erlandson, J.M.
Measuring subsistence specialization: Comparing historic and prehistoric abalone middens on San Miguel Island, California
(2007) Journal of Anthropological Archaeology, 26 (3), pp. 474-485. Cited 8 times.

DOI: 10.1016/j.jaa.2006.11.005

452. Petty, A.M., Werner, P.A., Lehmann, C.E.R., Riley, J.E., Banfai, D.S., Elliott, L.P.
Savanna responses to feral buffalo in Kakadu National Park, Australia
(2007) Ecological Monographs, 77 (3), pp. 441-463. Cited 42 times.

DOI: 10.1890/06-1599.1

453. Asquith, P.J.
Sources for Imanishi Kinji's views of sociality and evolutionary outcomes
(2007) Journal of Biosciences, 32 (4), pp. 635-641. Cited 1 time.

DOI: 10.1007/s12038-007-0063-7

454. Cousins, S.A.O., Ohlson, H., Eriksson, O.
Effects of historical and present fragmentation on plant species diversity in semi-natural grasslands in Swedish rural landscapes
(2007) Landscape Ecology, 22 (5), pp. 723-730. Cited 73 times.

DOI: 10.1007/s10980-006-9067-1

455. Köster, D., Lichter, J., Lea, P.D., Nurse, A.
Historical eutrophication in a river-estuary complex in mid-coast Maine
(2007) Ecological Applications, 17 (3), pp. 765-778. Cited 15 times.

DOI: 10.1890/06-0815

456. Thomson, A.M., Simpson, I.A.
Modeling historic rangeland management and grazing pressures in landscapes of settlement
(2007) Human Ecology, 35 (2), pp. 151-168. Cited 6 times.

DOI: 10.1007/s10745-006-9055-8

457. Bowman, D.M.J.S., Dingle, J.K., Johnston, F.H., Parry, D., Foley, M.
Seasonal patterns in biomass smoke pollution and the mid 20th-century transition from Aboriginal to European fire management in northern Australia
(2007) Global Ecology and Biogeography, 16 (2), pp. 246-256. Cited 19 times.

DOI: 10.1111/j.1466-8238.2006.00271.x

458. Woollett, J.
Labrador inuit subsistence in the context of environmental change: An initial landscape history perspective
(2007) American Anthropologist, 109 (1), pp. 69-84. Cited 15 times.

DOI: 10.1525/aa.2007.109.1.69

459. McGovern, T.H., Vésteinsson, O., Fridriksson, A., Church, M., Lawson, I., Simpson, I.A., Einarsson, A., Dugmore, A., Cook, G., Perdikaris, S., Edwards, K.J., Thomson, A.M., Adderley, W.P., Newton, A., Lucas, G., Edvarsson, R., Aldred, O., Dunbar, E.
Landscapes of settlement in Northern Iceland: Historical ecology of human impact and climate fluctuation on the Millennial scale
(2007) American Anthropologist, 109 (1), pp. 27-51. Cited 61 times.

DOI: 10.1525/aa.2007.109.1.27

460. Heckenberger, M.J., Russell, J.C., Toney, J.R., Schmidt, M.J.
The legacy of cultural landscapes in the Brazilian Amazon: Implications for biodiversity
(2007) Philosophical Transactions of the Royal Society B: Biological Sciences, 362 (1478), pp. 197-208. Cited 80 times.

DOI: 10.1098/rstb.2006.1979

2006

461. Erlandson, J. M., & Fitzpatrick, S. M. (2006). Oceans, islands, and coasts: Current perspectives on the role of the sea in human prehistory. Journal of Island & Coastal Archaeology, 1(1), 5-32.

DOI: 10.1080/15564890600639504

462. Moss, M.L., Yang, D.Y., Newsome, S.D., Speller, C.F., McKechnie, I., McMillan, A.D., Losey, R.J. and Koch, P.L.,
Historical ecology and biogeography of North Pacific pinnipeds: Isotopes and ancient DNA from three archaeological assemblages.
(2006) Journal of Island & Coastal Archaeology, 1(2), pp.165-190.

463. Abbott, I.
Mammalian faunal collapse in Western Australia, 1875-1925: The hypothesised role of epizootic disease and a conceptual model of its origin, introduction, transmission, and spread
(2006) Australian Zoologist, 33 (4), pp. 530-561. Cited 26 times.

464. Boyd, M., Hamilton, S., Running IV, G.L.
Reconstructing a prairie-woodland mosaic on the northern Great Plains: Risk, resilience, and resource management
(2006) Plains Anthropologist, 51 (199), pp. 235-252. Cited 3 times.

465. Smirnov, N.G.
Dynamics of species and species complexes as a field of research in historical ecology
(2006) Russian Journal of Ecology, 37 (6), pp. 414-418.

DOI: 10.1134/S1067413606060087

466. Storm, L., Shebitz, D.
Evaluating the purpose, extent, and ecological restoration applications of indigenous burning practices in Southwestern Washington
(2006) Ecological Restoration, 24 (4), pp. 256-268. Cited 23 times.

467. Brook, B.W., Bowman, D.M.J.S.
Postcards from the past: Charting the landscape-scale conversion of tropical Australian savanna to closed forest during the 20th century
(2006) Landscape Ecology, 21 (8), pp. 1253-1266. Cited 45 times.

DOI: 10.1007/s10980-006-0018-7

468. Piessens, K., Hermy, M.
Does the heathland flora in north-western Belgium show an extinction debt?
(2006) Biological Conservation, 132 (3), pp. 382-394. Cited 36 times.

DOI: 10.1016/j.biocon.2006.04.032

469. Sáenz-Arroyo, A., Roberts, C.M., Torre, J., Cariño-Olvera, M., Hawkins, J.P.
The value of evidence about past abundance: Marine fauna of the Gulf of California through the eyes of 16th to 19th century travellers
(2006) Fish and Fisheries, 7 (2), pp. 128-146. Cited 53 times.

DOI: 10.1111/j.1467-2979.2006.00214.x

470. Lunt, I.D., Jones, N., Spooner, P.G., Petrow, M.
Effects of European colonization on indigenous ecosystems: Post-settlement changes in tree stand structures in Eucalyptus-Callitris woodlands in central New South Wales, Australia
(2006) Journal of Biogeography, 33 (6), pp. 1102-1115. Cited 44 times.

DOI: 10.1111/j.1365-2699.2006.01484.x

2005

471. Kirch, P.V.
Archaeology and global change: The Holocene record
(2005) Annual Review of Environment and Resources, 30, pp. 409-440. Cited 91 times.

DOI: 10.1146/annurev.energy.29.102403.140700

472. Brook, B.W., Bowman, D.M.J.S., Bradshaw, C.J.A.
Mapping the future: Spatial models of decadal-scale landscape change in Northern Australia
(2005) MODSIM05 - International Congress on Modelling and Simulation: Advances and Applications for Management and Decision Making, Proceedings, pp. 1361-1367.

473. Thomson, A.M., Simpson, I.A., Brown, J.L.
Sustainable rangeland grazing in Norse Faroe
(2005) Human Ecology, 33 (5), pp. 737-761. Cited 8 times.

DOI: 10.1007/s10745-005-7596-x

474. Adderley, W.P., Simpson, I.A.
Early-norse home-field productivity in the Faroe Islands
(2005) Human Ecology, 33 (5), pp. 711-736. Cited 14 times.

DOI: 10.1007/s10745-005-6423-8

475. Sáenz-Arroyo, A., Roberts, C.M., Torre, J., Cariño-Olvera, M., Enríquez-Andrade, R.R.
Rapidly shifting environmental baselines among fishers of the Gulf of California
(2005) Proceedings of the Royal Society B: Biological Sciences, 272 (1575), pp. 1957-1962. Cited 128 times.

DOI: 10.1098/rspb.2005.3175

476. Iannuzzi, T.J., Ludwig, D.F.
An interdisciplinary investigation of ecological history and environmental restoration objectives in an urban landscape
(2005) Ecological Restoration, 23 (3), pp. 157-166. Cited 3 times.

477. Abrams, M.D.
Prescribing fire in eastern oak forests: Is time running out?
(2005) Northern Journal of Applied Forestry, 22 (3), pp. 190-196. Cited 52 times.

478. Sáenz-Arroyo, A., Roberts, C.M., Torre, J., Cariño-Olvera, M.
Using fishers' anecdotes, naturalists' observations and grey literature to reassess marine species at risk: The case of the Gulf grouper in the Gulf of California, Mexico
(2005) Fish and Fisheries, 6 (2), pp. 121-133. Cited 86 times.

DOI: 10.1111/j.1467-2979.2005.00185.x

479. Rouvinen, S., Rautiainen, A., Kouki, J.
A relation between historical forest use and current dead woody material in a boreal protected old-growth forest in Finland
(2005) Silva Fennica, 39 (1), pp. 21-36. Cited 11 times.

480. Alard, D., Chabrerie, O., Dutoit, T., Roche, P., Langlois, E.
Patterns of secondary succession in calcareous grasslands: Can we distinguish the influence of former land uses from present vegetation data?
(2005) Basic and Applied Ecology, 6 (2), pp. 161-173. Cited 37 times.

DOI: 10.1016/j.baae.2005.01.010

481. Dutoit, T., Forey, E., Römermann, C., Buisson, E., Fadda, S., Saatkamp, A., Gaignard, P., Trivelly, E.
Persistence of former land-uses and conservation management of calcareous grasslands in France [Rémanence des utilisations anciennes et gestion conservatoire des pelouses calcicoles en France]
(2005) Biotechnology, Agronomy and Society and Environment, 9 (2), pp. 125-132. Cited 4 times.

2004

482. Vanwalleghem, T., Verheyen, K., Hermy, M., Poesen, J., Deckers, J.
Legacies of Roman land-use in the present-day vegetation in Meerdaal Forest (Belgium)?
(2004) Belgian Journal of Botany, 137 (2), pp. 181-187. Cited 13 times.

483. Short, J.
Mammal decline in southern Western Australia - Perspectives from Shortridge's collections of mammals in 1904-07
(2004) Australian Zoologist, 32 (4), pp. 605-624. Cited 6 times.

484. Schroeder, S.
Power and place: Agency, ecology, and history in the American Bottom, Illinois
(2004) Antiquity, 78 (302), pp. 812-827. Cited 11 times.

485. Whitney, G.G., Upmeyer, M.M.
Sweet trees, sour circumstances: The long search for sustainability in the North American maple products industry
(2004) Forest Ecology and Management, 200 (1-3), pp. 313-333. Cited 9 times.

DOI: 10.1016/j.foreco.2004.07.006

486. Lotze, H.K., Milewski, I.
Two centuries of multiple human impacts and successive changes in a north Atlantic food web
(2004) Ecological Applications, 14 (5), pp. 1428-1447. Cited 101 times.

487. Pearce, S.A., Grossinger, R.M.
Relative effects of fluvial processes and historical land use on channel morphology in three sub-basins, Napa River basin, California, USA
(2004) IAHS-AISH Publication, (288), pp. 170-178.

488. Bickford, S., Mackey, B.
Reconstructing pre-impact vegetation cover in modified landscapes using environmental modelling, historical surveys and remnant vegetation data: A case study in the Fleurieu Peninsula, South Australia
(2004) Journal of Biogeography, 31 (5), pp. 787-805. Cited 9 times.

489. Swetnam, R.D., Mountford, J.O., Manchester, S.J., Broughton, R.K.
Agri-environmental schemes: Their role in reversing floral decline in the Brue floodplain, Somerset, UK
(2004) Journal of Environmental Management, 71 (1), pp. 79-93. Cited 14 times.

DOI: 10.1016/j.jenvman.2004.01.006

490. Patten, M.A., Smith-Patten, B.D.
Linking the Salton Sea with its past: The history and avifauna of Lake Cahuilla
(2004) Studies in Avian Biology, (27), pp. 12-17. Cited 1 time.

491. Van Der Veken, S., Verheyen, K., Hermy, M.
Plant species loss in an urban area (Turnhout, Belgium) from 1880 to 1999 and its environmental determinants
(2004) Flora, 199 (6), pp. 516-523. Cited 36 times.

DOI: 10.1078/0367-2530-00180

492. Dutoit, T., Buisson, E., Roche, P., Alard, D.
Land use history and botanical changes in the calcareous hillsides of Upper-Normandy (north-western France): New implications for their conservation management
(2004) Biological Conservation, 115 (1), pp. 1-19. Cited 26 times.

DOI: 10.1016/S0006-3207(03)00089-2

493. Buisson, E., & Dutoit, T. (2004). Colonisation by native species of abandoned farmland adjacent to a remnant patch of Mediterranean steppe. Plant Ecology, 174, 371-384.

DOI: 10.1007/BF03543067

2003

494. Pellerin, S., Lavoie, C.
Reconstructing the recent dynamics of mires using a multitechnique approach
(2003) Journal of Ecology, 91 (6), pp. 1008-1021. Cited 44 times.

DOI: 10.1046/j.1365-2745.2003.00834.x

495. Simpson, I.A., Vésteinsson, O., Adderley, W.P., McGovern, T.H.
Fuel resource utilisation in landscapes of settlement
(2003) Journal of Archaeological Science, 30 (11), pp. 1401-1420. Cited 39 times.

DOI: 10.1016/S0305-4403(03)00035-9

496. Verheyen, K., Guntenspergen, G.R., Beisbrouck, B., Hermy, M.
An integrated analysis of the effects of past land use on forest herb colonization at the landscape scale
(2003) Journal of Ecology, 91 (5), pp. 731-742. Cited 100 times.

DOI: 10.1046/j.1365-2745.2003.00807.x

497. Laliberte, A.S., Ripple, W.J.
Wildlife Encounters by Lewis and Clark: A Spatial Analysis of Interactions between Native Americans and Wildlife
(2003) BioScience, 53 (10), pp. 994-1003. Cited 26 times.

498. Loo, J., Ives, N.
The Acadian forest: Historical condition and human impacts
(2003) Forestry Chronicle, 79 (3), pp. 462-474. Cited 50 times.

499. Suffling, R., Evans, M., Perera, A.
Presettlement forest in southern Ontario: Ecosystems measured through a cultural prism
(2003) Forestry Chronicle, 79 (3), pp. 485-501. Cited 9 times.

500. MacDougall, A.
Did Native Americans influence the northward migration of plants during the Holocene?
(2003) Journal of Biogeography, 30 (5), pp. 633-647. Cited 10 times.

DOI: 10.1046/j.1365-2699.2003.00842.x

501. German, L.A.
Historical contingencies in the coevolution of environment and livelihood: Contributions to the debate on Amazonian Black Earth
(2003) Geoderma, 111 (3-4), pp. 307-331. Cited 45 times.

DOI: 10.1016/S0016-7061(02)00270-7

2002

502. Simpson, I.A., Adderley, W.P, Gŭptmundsson, G., Hallsdóttir, M., Sigurgeirsson, M.Á., Snæsdóttir, M.
Soil limitations to agrarian land production in premodern Iceland
(2002) Human Ecology, 30 (4), pp. 423-443. Cited 25 times.

DOI: 10.1023/A:1021161006022

503. Cogbill, C.V., Burk, J., Motzkin, G.
The forests of presettlement New England, USA: Spatial and compositional patterns based on town proprietor surveys
(2002) Journal of Biogeography, 29 (10-11), pp. 1279-1304. Cited 117 times.

DOI: 10.1046/j.1365-2699.2002.00757.x

504. Vera, F.W.M.
The dynamic European forest
(2002) Arboricultural Journal, 26 (3), pp. 179-211. Cited 9 times.

505. Petit, C.C., Lambin, E.F.
Long-term land-cover changes in the Belgian Ardennes (1775-1929): Model-based reconstruction vs. historical maps
(2002) Global Change Biology, 8 (7), pp. 616-630. Cited 46 times.

DOI: 10.1046/j.1365-2486.2002.00500.x

506. Girard, M., Lavoie, C., Thériault, M.
The regeneration of a highly disturbed ecosystem: A mined peatland in southern Québec
(2002) Ecosystems, 5 (3), pp. 274-288. Cited 62 times.

507. Romero, A., Baker, R., Creswell, J.E., Singh, A., McKie, A., Manna, M.
Environmental history of marine mammal exploitation in Trinidad and Tobago, W.I., and its ecological impact
(2002) Environment and History, 8 (3), pp. 255-274. Cited 10 times.

508. Brown, D.
The foulmart: What's in a name?
(2002) Mammal Review, 32 (2), pp. 145-149. Cited 3 times.

DOI: 10.1046/j.1365-2907.2002.00104.x

2001

509. Manlius, N.
The ostrich in Egypt: Past and present
(2001) Journal of Biogeography, 28 (8), pp. 945-953. Cited 8 times.

DOI: 10.1046/j.1365-2699.2001.00599.x

510. Jackson, J.B.C., Sala, E.
Unnatural Oceans
(2001) Scientia Marina, 65 (SUPPLEMENT 2), pp. 273-281. Cited 43 times.

511. Verheyen, K., Hermy, M.
The relative importance of dispersal limitation of vascular plants in secondary forest succession in Muizen Forest, Belgium
(2001) Journal of Ecology, 89 (5), pp. 829-840. Cited 95 times.

DOI: 10.1046/j.0022-0477.2001.00596.x

512. Main, B.Y.
Historical ecology, responses to current ecological changes and conservation of Australian spiders
(2001) Journal of Insect Conservation, 5 (1), pp. 9-25. Cited 12 times.

DOI: 10.1023/A:1011337914457

513. Marlin, J.C., LaBerge, W.E.
The native bee fauna of carlinville, Illinois, revisited after 75 years: A case for persistence
(2001) Conservation Ecology, 5 (1), . Cited 19 times.

514. Simpson, I.A., Dugmore, A.J., Thomson, A., Vésteinsson, O.
Crossing the thresholds: Human ecology and historical patterns of landscape degradation
(2001) Catena, 42 (2-4), pp. 175-192. Cited 53 times.

DOI: 10.1016/S0341-8162(00)00137-5

515.Manlius, N.
Historical ecology and biogeography of the Nubian Ibex in Egypt [Biogéographie et Ecologie historique du bouquetin de Nubie en Egypte]
(2001) Belgian Journal of Zoology, 131 (2), pp. 159-172. Cited 1 time.

516. Verheyen, K., Hermy, M.
An integrated analysis of the spatio-temporal colonization patterns of forest plant species
(2001) Journal of Vegetation Science, 12 (4), pp. 567-578. Cited 50 times.

2000

517. Kettle, W.D., Rich, P.M., Kindscher, K., Pittman, G.L., Fu, P.

Land-use history in ecosystem restoration: A 40-year study in the prairie-forest ecotone
(2000) Restoration Ecology, 8 (3), pp. 307-317. Cited 29 times.

DOI: 10.1046/j.1526-100X.2000.80043.x

518. Cogbill, C.V.
Vegetation of the presettlement forests of northern New England and New York
(2000) Rhodora, 102 (911), pp. 250-276. Cited 28 times.

519. Witt, G.B., Berghammer, L.J., Beeton, R.J.S., Moll, E.J.
Retrospective monitoring of rangeland vegetation change: Ecohistory from deposits of sheep dung associated with shearing sheds
(2000) Austral Ecology, 25 (3), pp. 260-267. Cited 10 times.

DOI: 10.1046/j.1442-9993.2000.01027.x

520. Manlius, N.
Historical ecology and biogeography of the hippopotamus in Egypt [Biogeographie et Ecologie historique de l'hippopotame en Egypte]
(2000) Belgian Journal of Zoology, 130 (1), pp. 59-66. Cited 6 times.

1999

521. Sheail, J.
Creating landscapes from the old - an English perspective on nature conservation
(1999) Norsk Geografisk Tidsskrift, 53 (3), pp. 71-76.

522. Hermy, M., Honnay, O., Firbank, L., Grashof-Bokdam, C., Lawesson, J.E.
An ecological comparison between ancient and other forest plant species of Europe, and the implications for forest conservation
(1999) Biological Conservation, 91 (1), pp. 9-22. Cited 331 times.

DOI: 10.1016/S0006-3207(99)00045-2

523. Verheyen, K., Bossuyt, B., Hermy, M., Tack, G.
The land use history (1278-1990) of a mixed hardwood forest in western Belgium and its relationship with chemical soil characteristics
(1999) Journal of Biogeography, 26 (5), pp. 1115-1128. Cited 145 times.

DOI: 10.1046/j.1365-2699.1999.00340.x

524. Manlius, N., Gautier, A.
The wild boar in Egypt [Le sanglier en Egypte]
(1999) Comptes Rendus de l'Academie des Sciences - Serie III, 322 (7), pp. 573-577. Cited 7 times.

DOI: 10.1016/S0764-4469(00)88527-3

525. Motzkin, G., Patterson III, W.A., Foster, D.R.
A historical perspective on pitch pine - Scrub oak communities in the Connecticut Valley of Massachusetts
(1999) Ecosystems, 2 (3), pp. 255-273. Cited 50 times.

DOI: 10.1007/s100219900073

526. Honnay, O., Hermy, M., Coppin, P.
Impact of habitat quality on forest plant species colonization
(1999) Forest Ecology and Management, 115 (2-3), pp. 157-170. Cited 141 times.

DOI: 10.1016/S0378-1127(98)00396-X

527. Parsons, D.J., Swetnam, T.W., Christensen, N.L.
Uses and limitations of historical variability concepts in managing ecosystems
(1999) Ecological Applications, 9 (4), pp. 1177-1178. Cited 25 times.

528. Stockwell, K.D.
Structure and history of the Atlantic white-cedar stands at Appleton Bog, Knox County, Maine, USA
(1999) Natural Areas Journal, 19 (1), pp. 47-56. Cited 5 times.

529. Desender, K., Ervynck, A., Tack, G.
Beetle diversity and historical ecology of woodlands in Flanders
(1999) Belgian Journal of Zoology, 129 (1), pp. 139-155. Cited 74 times.

530. Schjellerup, I.
Wayko - Lamas: A Quechua community in the Selva Alta of North Peru under change
(1999) Geografisk Tidsskrift, (SPEC. ISS. 1), pp. 199-207.

531. Bossuyt, B., Hermy, M., Deckers, J.
Migration of herbaceous plant species across ancient-recent forest ecotones in central Belgium
(1999) Journal of Ecology, 87 (4), pp. 628-638. Cited 182 times.

532. Swetnam TW, Allen CD, Betancourt JL
Applied Historical Ecology: Using the Past to Manage for the Future
(1999) Ecological Applications, Vol. 9, No. 4, pp. 1189-1206

1997

533. Amorosi, T., Buckland, P., Dugmore, A., Ingimundarson, J.H., McGovern, T.H.
Raiding the Landscape: Human Impact in the Scandinavian North Atlantic
(1997) Human Ecology, 25 (3), pp. 491-518. Cited 31 times.

534. Wulf, M.
Plant species as indicators of ancient woodland in northwestern Germany
(1997) Journal of Vegetation Science, 8 (5), pp. 635-642. Cited 113 times.

535. Dinsdale, J., Dale, P., Kent, M.
The biogeography and historical ecology of Lobelia urens L. (the heath lobelia) in southern England
(1997) Journal of Biogeography, 24 (2), pp. 153-175. Cited 6 times.

1996

536. Andersen, O., Crow, T.R., Lietz, S.M., Stearns, F.

Transformation of a landscape in the upper mid-west, USA: The history of the lower St. Croix river valley, 1830 to present
(1996) Landscape and Urban Planning, 35 (4), pp. 247-267. Cited 38 times.

DOI: 10.1016/S0169-2046(96)00304-0

537. Amorosi, T., Woollett, J., Perdikaris, S., McGovern, T.
Regional zooarchaeology and global change: Problems and potentials
(1996) World Archaeology, 28 (1), pp. 126-157. Cited 25 times.

1995

538. Pimm, S.L., Askins, R.A.
Forest losses predict bird extinctions in eastern North America
(1995) Proceedings of the National Academy of Sciences of the United States of America, 92 (20), pp. 9343-9347. Cited 133 times.

DOI: 10.1073/pnas.92.20.9343

539. Dutoit, T., Alard, D.
Mechanism of a secondary plant succession in chalk grassland: An historical approach [MECANISME D'UNE SUCCESSION VEGETALE SECONDAIRE EN PELOUSE CALCICOLE: UNE APPROCHE HISTORIQUE]
(1995) Comptes Rendus de l'Academie des Sciences - Serie III, 318 (8), pp. 897-907. Cited 17 times.

1994

540. Gunn, J.D.
Introduction: A perspective from the humanities-science boundary
(1994) Human Ecology, 22 (1), pp. 1-22. Cited 4 times.

DOI: 10.1007/BF02168760

1993

541. van Dam, H., Buskens, R.F.M.
Ecology and management of moorland pools: balancing acidification and eutrophication
(1993) Hydrobiologia, 265 (1-3), pp. 225-263. Cited 29 times.

DOI: 10.1007/BF00007271

1992

542. Hammett, J.E.
The shapes of adaptation: Historical ecology of anthropogenic landscapes in the southeastern United States
(1992) Landscape Ecology, 7 (2), pp. 121-135. Cited 34 times.

DOI: 10.1007/BF02418943

1986

543. Smart, P. J., Wheeler, B. D., & Willis, A. J. (1986). Plants and peat cuttings: historical ecology of a much exploited peatland□thorne waste, yorkshire, uk. New Phytologist, 104(4), 731-748.

1981

544. Hermy, M., Stieperaere, H.
An indirect gradient analysis of the ecological relationships between ancient and recent reiverine woodlands to the south of Bruges (Flanders, Belgium)
(1981) Vegetatio, 44 (1), pp. 43-49. Cited 50 times.

DOI: 10.1007/BF00119802
